# ApoE4 disrupts intracellular trafficking and iron homeostasis in a reproducible iPSC-based model of human brain endothelial cells

**DOI:** 10.1016/j.stemcr.2025.102607

**Published:** 2025-08-21

**Authors:** Luisa Bell, Shane Clerkin, Sila Rizalar, Antoine Rizkallah, Nadine Stokar-Regenscheit, Xandor M. Spijkers, Nienke R. Wevers, Claire Simonneau, Angélique Augustin, Barbara Höllbacher, Lia D’Abate, Joanna Ficek-Pascual, Kim Schneider, Desiree Von Tell, Thomas Maurissen, Chiara Zanini, Christelle Zundel, Sabrina Golling, Christine Becker, Alex Odermatt, Lynette C. Foo, Martina Pigoni, Roberto Villaseñor

**Affiliations:** 1 Roche Pharma Research and Early Development (pRED), Roche Innovation Center Basel, Basel, Switzerland; 2 Department of Pharmaceutical Sciences, University of Basel, Basel, Switzerland; 3 MIMETAS BV, Oegstgeest, the Netherlands

**Keywords:** blood-brain barrier, neuroscience, APOE4, endothelial cells

## Abstract

Transferrin receptor in brain endothelial cells can deliver therapeutic antibodies to the brain via transcytosis across the blood-brain barrier (BBB). Whether receptor transport remains intact in Alzheimer disease is still a major open question. Here, we investigated whether apolipoprotein E4 (ApoE4), the major genetic risk factor for Alzheimer disease, altered intracellular transport in human brain endothelial cells. To achieve this, we first developed a reproducible protocol for induced pluripotent stem cells based on a defined chemical cocktail and extracellular matrix support to differentiate brain endothelial cells (iCE-BECs). Multi-omics profiling and functional transport assays showed that iCE-BECs have a brain endothelial gene signature and recapitulate receptor-mediated transcytosis of a clinically validated Brainshuttle antibody against transferrin receptor. Engineered iCE-BECs homozygous for ApoE4 had impaired endosome maturation, increased transferrin receptor expression, and reduced cytoplasmic iron. Our data revealed that ApoE4 can impact intracellular transport and iron homeostasis at the BBB in a cell-autonomous manner.

## Highlights

• New protocol for differentiation of brain endothelial cells (iCE-BECs) from iPSC
• iCE-BECs recapitulate receptor-mediated transcytosis of anti-TfR1 antibodies
• ApoE4 impairs endosome maturation in iCE-BECs
• ApoE4 cell-autonomously regulates iron homeostasis in iCE-BECs

## Teaser

Bell and colleagues develop a new protocol to differentiate brain endothelial cells from iPSC to investigate the regulation of intracellular transport across the blood-brain barrier. They find that ApoE4 impairs endosomal maturation and increases endosomal pH in brain endothelial cells. This leads to reduced cytosolic iron, which alters the expression of iron transporters, including TfR1.

## Introduction

The blood-brain barrier (BBB) is formed by brain endothelial cells, pericytes, and astrocytes organized into a neurovascular unit that regulates the exchange of proteins between blood circulation and brain parenchyma via receptor-mediated transcytosis (Abbott 2013). The transferrin receptor (TfR1) is one of the best characterized receptors involved in transcytosis and is validated as a target to deliver therapeutic antibodies to the brain parenchyma (Bien-Ly et al., 2014; Grimm et al., 2023). Intracellular sorting in endosomes determines whether a receptor undergoes transcytosis or is instead transported to lysosomes for degradation (Villaseñor et al., 2019). Whether intracellular trafficking in brain endothelial cells is altered in disease conditions and to what extent this impacts transcytosis across the BBB remains largely unknown.

There is a wealth of evidence that documents the impact of disease conditions on BBB paracellular permeability. For example, it is well established that ApoE4, the major genetic risk factor for sporadic Alzheimer disease (AD), increases paracellular permeability of the BBB *in vivo* (Halliday et al., 2016; Yamazaki et al., 2020; Montagne et al., 2021). On the other hand, disease-specific changes to intracellular transport and/or transcytosis across the BBB are still relatively unexplored. A recent transcriptomic analysis of the vasculature of AD brain tissue showed substantial changes in gene expression levels associated with intracellular trafficking, including downregulation of TfR1 in brain capillaries (Yang et al., 2022). However, disease subgroups or risk factor genotypes could further affect TfR1 expression and trafficking. For instance, it is known that ApoE4 expression in mice led to upregulation of TfR1 expression in brain endothelial cells (Barisano et al., 2022). This highlights the need for a systematic approach to evaluate how specific risk factors affect transport across the BBB.

Human stem-cell-based models using brain endothelial cells are a powerful tool to investigate how disease-related conditions might affect BBB integrity (Blanchard et al., 2020). However, earlier studies used models with an overt epithelial signature that lacked expression of genes required for endothelial function (Lu et al., 2021). Since the mechanisms of intracellular transport in endothelial and epithelial cells are different (Villaseñor et al., 2019), induced pluripotent stem cells (iPSCs)-based models with epithelial identity are not suitable to investigate the regulation of transcytosis across the BBB. To address this, we developed a protocol to differentiate endothelial cells with brain-specific identity from iPSCs. We show that these cells express key markers of brain endothelial cells and recapitulate receptor-mediated transcytosis of a clinically validated TfR1-dependent Brainshuttle antibody. Using this differentiation protocol on genetically engineered iPSC lines, we found that ApoE4 regulates the maturation of early endosomes in a cell-autonomous manner in brain endothelial cells. Finally, we show that ApoE4 also dysregulates iron homeostasis, triggering upregulation of TfR1 in brain endothelial cells. Our data highlight the utility of relevant human *in vitro* models of brain endothelial cells to investigate how disease risk factors affect intracellular transport and reveal a new role for ApoE4 in the regulation of iron metabolism at the BBB.

## Results

### Characterization and validation of a novel protocol for differentiation of brain endothelial cells from iPSC to investigate protein transport

**Figure 1:**
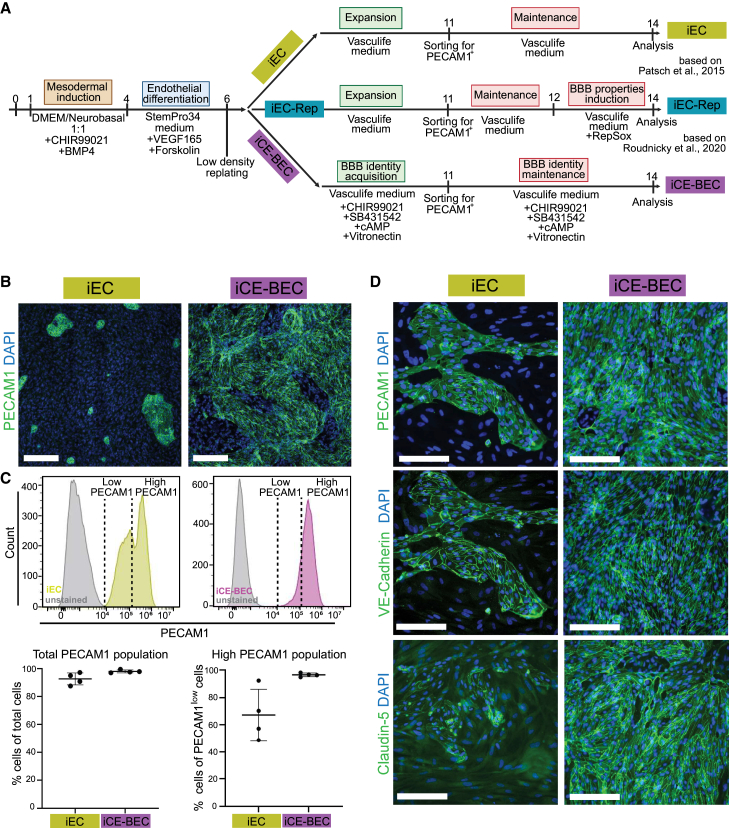
Differentiation of brain endothelial cells from induced pluripotent stem cells by a chemical cocktail and ECM support (iCE-BECs) (A) Schematic of the protocols used for iPSC differentiation as described in the experimental procedures. (B) Representative fluorescence images after immunostaining with the endothelial-specific marker PECAM1 of iEC and iCE-BECs before PECAM1 sorting at day 11. Cells are pseudo-colored showing PECAM1 in green and DAPI-stained nuclei in blue. Scale bars, 250 μm. (C) Representative flow cytometry histograms showing fluorescence intensity of PECAM1 on the *x* axis and the number of cells on the *y* axis from live iEC (yellow) and iCE-BECs (purple) and respective unstained controls (gray) after sorting at day 14. Vertical lines illustrate gating for low PECAM1 and high PECAM1 expression. Graphs show the mean ± SD percentage of PECAM1-positive cells of total cells and highly expressing PECAM1 cells in PECAM1-positive population after sorting at day 14 across four independent differentiations. (D) Representative fluorescence images after immunostaining with endothelial-specific markers of iEC and iCE-BECs at day 14. PECAM1, VE-Cadherin, or Claudin-5 are pseudo-colored in green and DAPI-stained nuclei in blue. Scale bars, 200 μm.

To generate human brain endothelial cells from iPSC, we first triggered mesoderm induction in the cells, as previously described (Patsch et al., 2015). Mesoderm-committed cells were next differentiated toward an endothelial identity using VEGF165 and forskolin. We then treated these cells with a defined chemical cocktail that modulates key pathways associated with induction of BBB properties during development: Wnt, cAMP, and transforming growth factor beta (TGF-β) (Figure 1A; methods for details). In addition to this chemical cocktail, we replated the cells on vitronectin-coated plates, as this extracellular matrix (ECM) glycoprotein has a key role in both maintaining barrier properties and regulating transcytosis in mice (Ayloo et al., 2022). We termed the cells generated by this protocol iCE-BECs (inducible differentiation via chemical cocktail and extracellular matrix support for brain endothelial cells). We compared iCE-BECs to human iPSC-derived endothelial cells obtained via mesoderm induction, referred here as iECs. The iCE-BEC protocol resulted in a higher number of PECAM1-positive cells at day 11 before cell sorting by MACS (Figures 1B and S1). After sorting by PECAM1 and culturing for 3 days in their respective maintenance media, the percentage of PECAM1-positive cells was close to 100% for both protocols (Figure 1C). However, iECs showed two populations expressing different levels of PECAM1 with high heterogeneity across experiments (Figures 1C and 1D). In contrast, the iCE-BEC protocol resulted in a homogeneous population of PECAM1-positive cells reproducibly across experiments (Figures 1C and 1D). Similarly, the expression of VE-Cadherin and Claudin-5 was heterogeneous in iECs and more homogeneous in iCE-BECs (Figure 1D). These data suggest that the iCE-BEC protocol leads to an endothelial cell population that is more homogeneous and reproducible compared to iECs.

**Figure 2:**
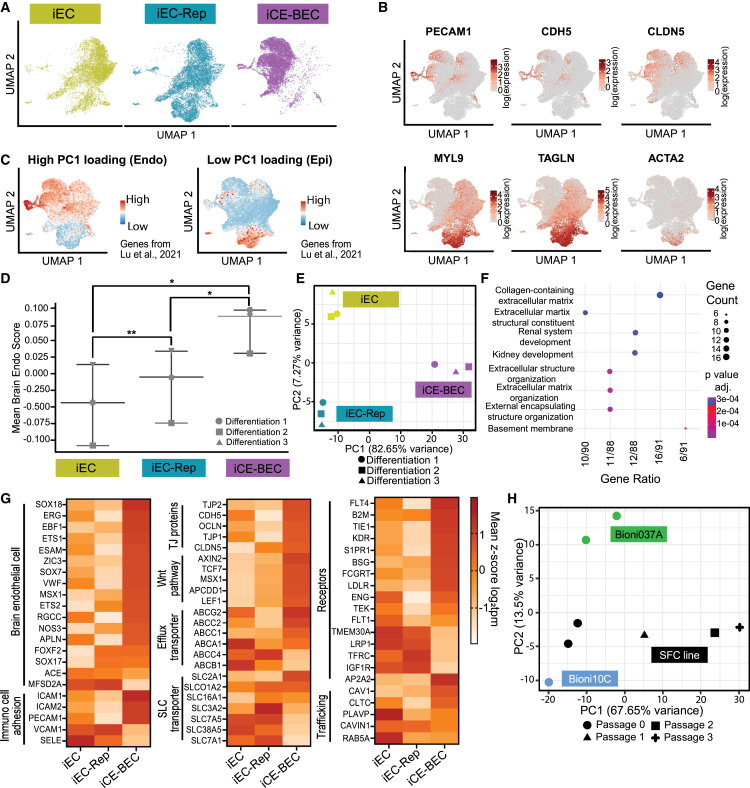
Transcriptomic signature of iCE-BECs (A) Integrated UMAP plot of scRNA-seq analysis, performed on the endothelial cells generated using the protocols summarized in Figure 1A. Data are generated from three independent differentiations per condition. (B) Selected feature plots showing normalized expression of marker genes of endothelial (*PECAM1*, *CDH5*, and *CLDN5*) and mural (*MYL9*, *TAGLN*, and *ACTA2*) markers, plotted on the UMAP from Figure 2A. (C) Feature plots showing expression of endothelial modules (Endo) “high PC1 loading” and epithelial module (Epi) “low PC1 loading” using the gene list described in (Lu et al., 2021) (see Table S1), plotted on the UMAP from 1A. (D) Mean ± range Brain Endo Score levels in cells expressing endothelial markers (see supplemental information for details) across the three protocols described in Figure 1A. ^∗∗^*p* < 0.01 and ^∗^*p* < 0.05 by one-way ANOVA followed by paired t test comparisons with FDR correction for multiple comparisons. (E) Principal component analysis (PCA) of bulk RNA-seq comparing iEC, iEC-Rep, and iCE-BECs from three independent differentiations each. (F) Gene ontology terms enriched in the principal component 1 from the PCA performed in Figure 2E. (G) Bulk RNA-seq heatmaps showing expression of genes associated with blood-brain barrier properties comparing iECs, iEC-Rep, and iCE-BECs. Values are expressed as mean *Z* score log2tpm, with three independent differentiations per condition. (H) PCA of iCE-BECs generated from three different iPSC lines (SFC086_03_03, Bioni037A, and Bioni10C) across four passages after differentiation.

Previous work showed that inhibition of TGF-β signaling by RepSox enhanced barrier properties in iECs (Roudnicky et al., 2020). We therefore asked whether iCE-BECs improved barrier properties beyond those triggered by RepSox alone. To this end, we compared iCE-BECs and iECs with iECs treated with RepSox (iEC-Rep; Figures 1A and S2A) using single-cell RNA sequencing (RNA-seq) and bulk RNA-seq. Analysis was performed on day 14 in culture, and data were integrated to assess the composition of the cell populations obtained with each protocol (Figure 2A).

Single-cell RNA-seq analysis showed that endothelial markers (e.g., *PECAM1*, *CDH5*, and *CLDN5*) were enriched in the cluster associated with iCE-BECs, whereas cells expressing mural cell markers (e.g., *MYL9*, *TAGLN*, and *ACTA2*) were enriched in iECs and iEC-Rep cells (Figures 2B and S2B). To characterize the cell identity of iCE-BECs, we evaluated the expression of a recently described list of genes (Lu et al., 2021) (see Table S1) associated either with an endothelial transcriptomic signature (high PC1 loading) or an epithelial transcriptomic identity (low PC1 loading). Endothelial signature genes were expressed across protocols and highly enriched in iCE-BECs, whereas epithelial signature genes were mildly expressed across the three protocols (Figure 2C). This transcriptomic signature is consistent with the highly heterogeneous expression of PECAM1 in iECs and could be explained by the presence of non-endothelial cells in the iEC protocol.

To specifically assess the brain identity in endothelial cells across protocols (i.e., independently of the potential presence of contaminating non-endothelial cells), we re-analyzed the data in cells expressing genes associated with endothelial identity (*PECAM1*, *KDR*, *VWF*, *ENG*, *CDH5*, *FLT4*, and *FCGRT*; see supplemental information for details). Within this population, we defined a brain endothelial score based on the expression of genes known to be enriched in brain endothelial cells (Yang et al., 2022) (*CLDN5*, *MFSD2A*, *SLC16A1*, *SLC3A2*, *SLC38A5*, *SLC7A5*, and *SLC2A1*; see supplemental information for details). We found that iCE-BECs had a higher brain endothelial score compared to iEC and iEC-Rep, suggesting that the iCE-BEC protocol improves the acquisition of a brain-like signature specifically in endothelial cells (Figure 2D).

We next performed bulk RNA-seq analysis to do a quantitative comparison of differentially expressed genes across protocols. Principal-component analysis showed a clear separation of the three different protocols, with PC1 accounting to 82.65% of the total variance (Figure 2E). Loading genes in PC1 were shown to be enriched for ECM-related pathways (Figure 2F). In agreement with the single-cell dataset, endothelial and brain endothelial cell markers were upregulated in iCE-BECs compared to iECs and iEC-Rep (Figure 2G), while epithelial and mural cell markers were not detected or strongly downregulated (Figure S2C). iCE-BECs showed an upregulation of *MSX1*, *ZIC3*, *EBF1*, and *APLN*, which are genes specifically enriched in brain endothelial cells in mice (Sabbagh et al., 2018). Additionally, iCE-BECs had an upregulation of genes associated with BBB function, including tight junction genes (*CLDN5*, *TJP1*, *TJP2*, *OCLN*, and *CDH5*), solute carriers (*SLC2A1*, *SLCO1A2*, and *SLC16A1*), efflux transporters (*ABCG2* and *ABCC2*), genes involved in immune cell adhesion (*ICAM1*, *ICAM2*, and *PECAM1*) and downstream effectors of Wnt signaling (*LEF1*, *TCF7*, *AXIN2*, and *APCDD1*) (Figure 2G). These data confirm that iCE-BECs exhibit a brain identity signature and express key genes essential for brain endothelial function. To confirm the reproducibility of the iCE-BEC protocol, we performed bulk RNA-seq in three different iPSC lines and 4 different iCE-BEC passage numbers (see methods for details). Principal component analysis shows that PC1 accounts for almost 68% of the variance and is driven by passage number after differentiation. In contrast, PC2, which is driven by the effects from the different parental lines, accounts for less than 15% of the total variance (Figure 2H). Moreover, just one of the genes associated with the BBB score defined above (*SLC38A5*) is among the top 100 loading genes in PC2 and none in PC1 (Table S2). This analysis confirms that the iCE-BEC protocol generates cells with brain endothelial-like identity across different parental iPSC lines.

**Figure 3:**
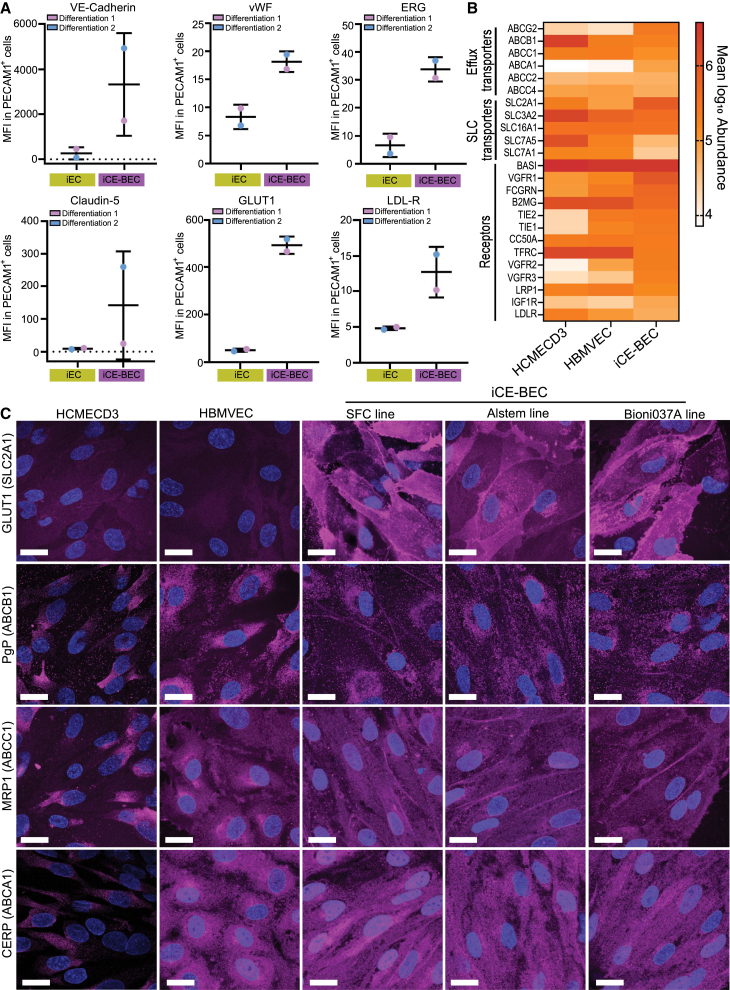
Proteomic signature of iCE-BECs (A) Flow cytometry measurements of protein expression in PECAM1-positive iEC and iCE-BECs. Median fluorescent intensity (MFI) of each marker was extracted and normalized to the MFI of their respective unstained control in each condition and differentiation. Data come from two differentiations; graphs show mean ± SD, each data point represents an average of a duplicate. (B) Whole-cell proteomics heatmap showing expression of brain endothelial receptors and transporters comparing immortalized human brain endothelial cells (HCMEC/D3, *n* = 3), primary human microvascular brain endothelial cells (HBMVEC, *n* = 6), and iCE-BECs (*n* = 4 independent differentiations). Values are expressed as mean log10 abundance. (C) Representative fluorescence images after immunostaining for SLC2A1, ABCB1, ABCC1, or ABCA1 in HCMEC/D3, HBMBVECs, and iCE-BECs from three different iPSC lines. Transporters are pseudo-colored in magenta and DAPI-stained nuclei in blue. Scale bars, 20 μm.

To validate the transcriptional changes observed in iCE-BECs, we performed flow cytometry analysis of selected proteins in PECAM1-positive cells (Figures 3A and S2D). We found that VE-Cadherin, vWF, ERG, Claudin-5, GLUT1, and LDL-R expression were upregulated in iCE-BECs compared to iECs. This result confirms the transcriptional data and shows that the iCE-BEC protocol increases the expression of proteins associated with brain identity.

To investigate the applicability of iCE-BECs for transport assays, we measured the expression of transporters and receptors with whole-cell proteomics. We compared these data to the proteome of both primary human brain microvascular endothelial cells (HBMVECs) and the widely used immortalized brain endothelial cell line, HCMEC/D3. We observed small changes in specific receptors that were upregulated (SLC2A1, ABCA1, and ABCG2) or downregulated (SLC7A1 and SLC7A5) in iCE-BECs. However, the proteomics analysis showed an overall similar expression of transporters and receptors between the three cell lines (Figure 3B). We confirmed the expression of SLC2A1, ABCB1, ABCC1, and ABCA1 by immunofluorescence (Figure 3C). SLC2A1 is clearly increased in iCE-BECs using three different parental iPSC lines compared to immortalized and primary brain endothelial cells (Figure 3C). Taken together, transcriptomics and proteomics data demonstrated that iCE-BECs are a homogeneous population of cells with human brain endothelial identity and express proteins relevant for the *in vitro* assessment of transcytosis across the BBB.

### iCE-BECs restrict the transport of large molecules and recapitulate receptor-mediated transcytosis

Next, we assessed the permeability of iCE-BECs. First, iCE-BECs showed a 50% increase in TEER values compared to iECs (Figure S3A). Second, permeability measurements on dextrans of different molecular weights (3, 40, and 70 kDa) in transwell chambers was decreased by 75% compared to both iECs and iEC-Rep (Figure S3B). Importantly, since iCE-BECs grew as a continuous monolayer in the transwell filter (Figure S3C), their observed decreased permeability is not due to the formation of multiple pseudo-stratified cell layers in the filter.

**Figure 4:**
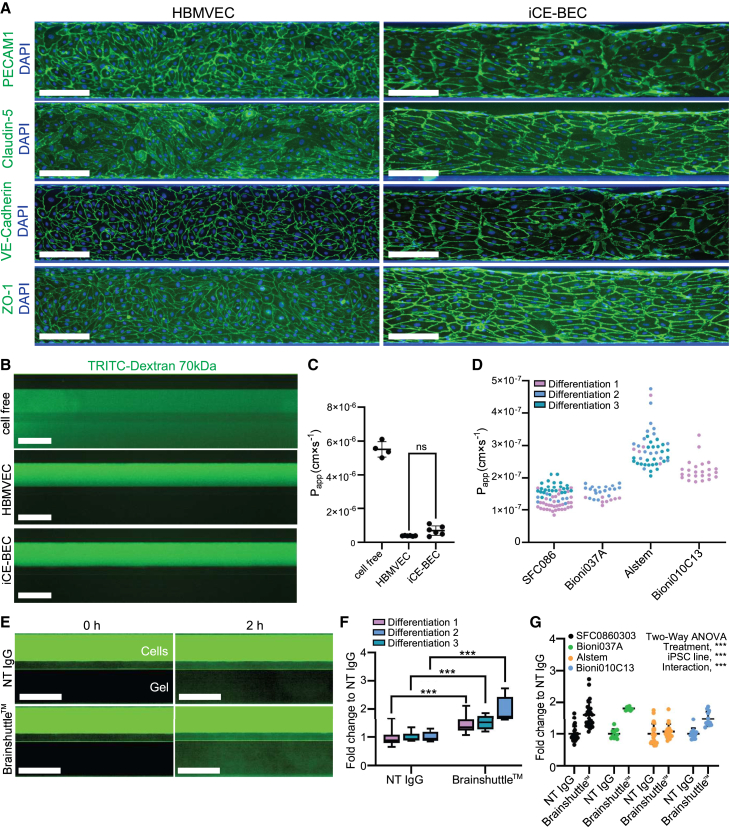
iCE-BECs show low permeability to large molecules and recapitulate receptor-mediated transcytosis of Brainshuttle antibodies (A) Representative fluorescence images after immunostaining with PECAM1, Claudin-5, VE-Cadherin, and ZO-1 (pseudo-colored in green) of HBMVECs and iCE-BECs grown in a MIMETAS OrganoPlate 3-lane 96. Scale bars, 100 μm. (B) Representative fluorescence images of MIMETAS OrganoPlate 2-lane chambers comparing cell-free (top), HBMVECs (middle), and iCE-BECs (bottom) after incubation with 70 kDa TRITC-dextran for 40 min. Scale bars, 100 μm. (C) Quantification of apparent permeability (P_app_) of cell-free, HBMVECs, and iCE-BECs to 70 kDa TRITC-dextran. Graph shows mean ± SD. ns, not statistically significant by Kruskal-Wallis test with Dunn’s multiple comparisons test; *p* = 0.146 with *n* = 4 independent chips from one lot of primary cells (HBMVECs) and one differentiation (iCE-BECs). (D) Quantification of P_app_ of iCE-BECs to 40 kDa dextran using MIMETAS OrganoPlate 2-lane 96 in iCE-BECs generated with different parental iPSC lines. Graph shows data from three differentiations; each data point represents one chip. (E) Representative fluorescence images of iCE-BECs grown on MIMETAS OrganoPlate 2-lane chambers comparing iEC (top) and iCE-BECs (bottom) after incubation with 200 nM non-targeting IgG (NT IgG) or a Brainshuttle antibody. Images were acquired immediately after incubation (0 h) or after 2 h. Scale bars, 500 μm. (F) Quantification of relative antibody transcytosis across iCE-BECs. Individual values are normalized to the mean of the NT-IgG condition. Graph shows boxplots with interquartile ranges and medians. Lines show the 5th and 95th percentiles. ^∗∗∗^*p* < 0.001, no significant effect of differentiation on transcytosis, significant interaction of treatment and differentiation; ^∗∗^*p* < 0.01 by a mixed effect analysis with Sidak’s multiple comparisons with *n* = 3 independent differentiations with at least seven independent chips per condition. (G) Quantification of relative antibody transcytosis across iCE-BECs generated with different parental iPSC lines after incubation with 200 nM non-targeting IgG (NT IgG) or Brainshuttle antibody. Graph shows mean ± SD of multiple differentiations. Each data point represents one chamber. Significant main effect of treatment on transcytosis, ^∗∗∗^*p* < 0.001; significant main effect of iPSC line on transcytosis, ^∗∗∗^*p* < 0.001; and significant interaction between iPSC line and treatment, ^∗∗∗^*p* < 0.001 by two-way ANOVA with *n* = 3 (SFC086), *n* = 2 (Bioni037A), *n* = 3 (Alstem), and *n* = 1 (Bioni010C13) independent differentiations with at least eight independent chips per condition.

The apparent reduction in permeability of iCE-BECs compared to iECs could be explained by heterogeneous iECs with lack of barrier properties. Therefore, to further characterize iCE-BEC permeability, we compared these cells to primary HBMVECs using a microfluidic platform (MIMETAS OrganoPlate) well established to investigate BBB transport (Wevers et al., 2018). As expected, both HBMVECs and iCE-BECs grown on the MIMETAS platform homogeneously expressed endothelial markers and tight junction proteins (Figure 4A). To evaluate paracellular permeability, we measured the flux of fluorescently labeled dextran over time (Figure 4B). We found that the apparent permeability of iCE-BECs to 70 kDa dextran was similar to HBMVECs and in the order of 10^−7^ cm⋅s^−1^ (Figures 4C and 4D; see supplemental information for details). Treatment of iCE-BECs with vascular endothelial growth factor (VEGF) increased the permeability to dextran, further confirming the endothelial identity of the cells (Figure S3D). We observed similar values in the order of 10^−7^ cm⋅s^−1^ across four different iPSC lines (Figure 4D). However, the Alstem iPSC line consistently showed higher P_app_ values. These results demonstrate that the low paracellular permeability of iCE-BECs can be recapitulated across multiple iPSC lines.

We next evaluated receptor-mediated transcytosis in iCE-BECs using the Brainshuttle (BrS), a validated monovalent bispecific antibody against TfR1 that effectively crosses the BBB (Grimm et al., 2023). In agreement with previously reported data for the same antibody in BBB organoids (Simonneau et al., 2021), we found that the BrS showed an almost 2-fold higher transport rate compared to a non-targeting immunoglobulin G (IgG) (Figures 4E, 4F, and S3E). This higher transport rate is not due to cell death or disruption of barrier integrity as incubation with the BrS antibody did not increase the permeability of 40 kDa dextran (Figure S3F). The extent of BrS transcytosis was reproducible across three different iPSC lines differentiated with the iCE-BEC protocol (Figure 4G). The lack of observed transcytosis of BrS in the Alstem iPSC line could be due to its higher basal paracellular permeability (Figure 4D). These data show that iCE-BECs recapitulate key features of the BBB, including low permeability to large molecules and receptor-mediated transcytosis. Together, our data show that iCE-BECs (1) have endothelial identity, (2) show a transcriptomic and proteomic brain-endothelial signature, and (3) recapitulate receptor-mediated transcytosis of antibodies. Therefore, we conclude that iCE-BECs are a suitable human *in vitro* system to investigate the regulation of intracellular transport across brain endothelial cells.

### ApoE4 impairs endosome maturation in iCE-BECs

ApoE4 is the major risk factor for sporadic AD (Armstrong 2019). Multiple studies show that ApoE4 can affect BBB function, likely via non-cell autonomous mechanisms (Montagne et al., 2020; Barisano et al., 2022). Nevertheless, it is unclear whether ApoE4 affects brain endothelial cells in a cell-autonomous manner (Blumenfeld et al., 2024). To address this, we used two pairs of isogenic iPSC lines with either a homozygous ApoE3 (Bioni037-A, Alstem iPS26) or a homozygous ApoE4 (Bioni037-A4, Alstem iPS16) gene variant and differentiated these into iCE-BECs. We refer here to the Bioni037 lines as isogenic Pair 1 and the Alstem lines as isogenic Pair 2. Importantly, ApoE4 did not change the proliferation rate of iPSCs (Figure S4) and did not impair the differentiation process as evidenced by expression of endothelial and tight junction genes (Figure S4). Both isogenic pairs expressed ApoE with an apparent mild mRNA and protein reduction in the ApoE4 lines (Figures S4E–S4G). These data suggest that ApoE4 iCE-BECs can be used to investigate cell-autonomous effects of ApoE4.

**Figure 5:**
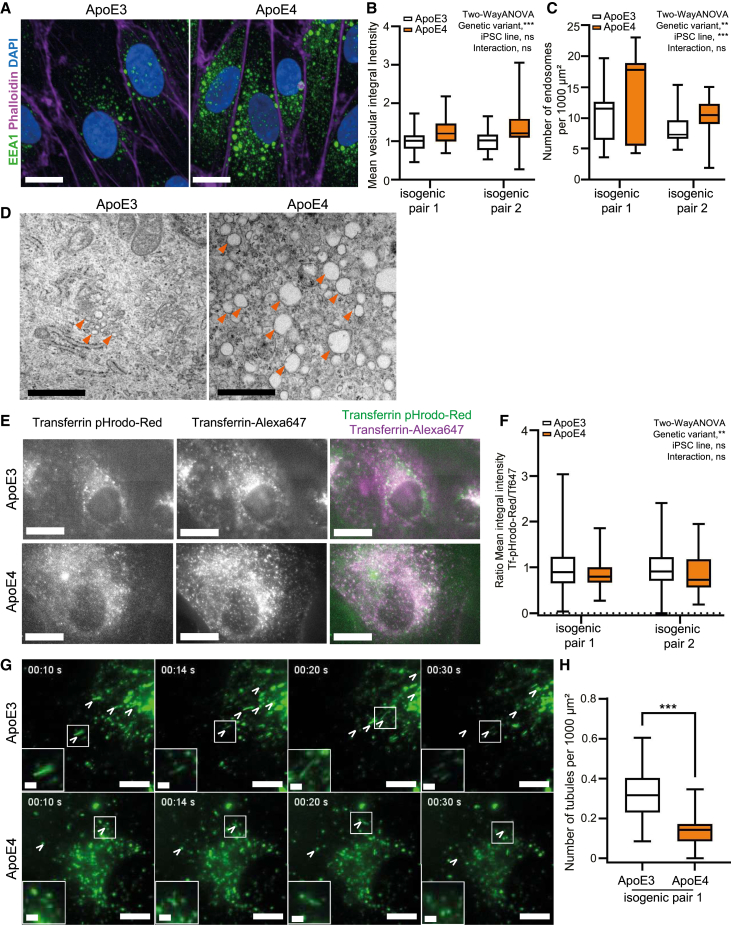
ApoE4 alters early endosome maturation in iCE-BECs (A) Representative maximum intensity projections images of iCE-BECs with ApoE3 or ApoE4 genetic variant showing EEA1 pseudo-colored in green, Phalloidin in magenta, and DAPI-stained nuclei in blue. Scale bars, 10 μm. (B and C) Quantification of mean integral vesicular intensity of EEA1 normalized to Phalloidin-positive area (B) or number of EEA1-positive endosomes per 1,000 μm^2^ Phalloidin-positive area in both isogenic pairs (C). Individual values in (B) were normalized to the mean of ApoE3 conditions for each experiment. Significant main effect of genetic variant on EEA1 intensity, ^∗∗∗^*p* < 0.001, no significant main effect of iPSC line on EEA1 intensity and no significant interaction between iPSC line and genetic variant. Significant main effect of genetic variant on endosome number, ^∗∗^*p* < 0.01; significant main effect of iPSC line on number of endosomes, ^∗∗∗^*p* < 0.001; no significant interaction between iPSC line and genetic variant by two-way ANOVA with *n* = 3 independent differentiations with approximately 200 cells per condition. (D) Representative transmission electron microscopy images of ApoE3 or ApoE4 iCE-BECs in isogenic pair 1. Arrowheads point to endosomes. Scale bars, 1 μm. (E) Representative images of transferrin in live iCE-BECs with ApoE gene variants after incubation with 25 μg/mL pH-sensitive Transferrin pHrodo Red and 25 μg/mL Transferrin-Alexafluor647 for 10 min. Scale bars, 20 μm. (F) Quantification of the ratio between Transferrin pHrodo Red (Tf-pHrodo-Red) and Transferrin-Alexafluor647 (Tf647) per endosome. Significant main effect of genetic variant on the ratio, ^∗∗^*p* < 0.01; no significant main effect of iPSC line on the ration; and no significant interaction between iPSC line and genetic variant on the ratio by two-way ANOVA with *n* = 3 independent differentiations with 30 cells per experiment for each isogenic pair. (G) Representative images of Tf (green) intracellular transport in live iCE-BECs with ApoE gene variants in isogenic pair 1 after incubation with 25 μg/mL of Tf-Alexafluor488 for 3 h. The time stamp shows the elapsed time in seconds after the acquisition of the first image. Arrowheads point to individual sorting tubules across time frames. Inserts depict a zoom-in on selected sorting tubules. Scale bars, 10 μm, inserts 2 μm. (H) Quantification of the mean number of sorting tubules per 1,000 μm^2^ occurring in one minute. ^∗∗∗^*p* < 0.001 by Student’s t test with *n* = 3 independent differentiations with 20 videos per experiment from isogenic pair 1. All graphs show boxplots with interquartile ranges and medians. Lines show the 5^th^ and 95^th^ percentiles.

Recent work showed that ApoE4 alters multiple endocytosis mechanisms *in vivo* (Nuriel et al., 2017; Barisano et al., 2022). Therefore, we analyzed the organization of endosomes in ApoE4 iCE-BECs in both isogenic pairs. We found that the number of early endosomes (labeled by EEA1) was higher in ApoE4 compared to ApoE3 iCE-BECs (Figures 5A and 5C). In addition, the total amount of EEA1 per endosome was also higher in ApoE4 compared to ApoE3 iCE-BECs (Figure 5B). We confirmed by transmission electron microscopy that ApoE4 iCE-BECs had an increase in endosome number and size compared to ApoE3 cells (Figure 5D). The changes in endosome number, size, and EEA1 amount in ApoE4 iCE-BECs could reflect impaired endosome maturation. To test this hypothesis, we used live-cell imaging to measure both endosomal pH and endosome sorting tubule biogenesis. We assessed endosomal pH by measuring the fluorescence intensity of transferrin (Tf) conjugated with a pH-sensitive dye in individual endosomes. The intensity per endosome was normalized to the fluorescence intensity from Tf conjugated to a non-pH sensitive dye. We found that ApoE4 iCE-BECs had increased signal for each of the Tf-fluorescent conjugates but a lower fluorescence ratio (Figures 5E and 5F). This result points to a higher uptake of Tf and to a higher endosomal pH (i.e., less acidic) in ApoE4 iCE-BECs. Next, we visualized the dynamics of fluorescent Tf to evaluate the formation of sorting tubules. In agreement with previous data on mouse brain endothelial cells (Villaseñor et al., 2017), we observed frequent events of sorting tubule biogenesis and vesicle fission in ApoE3 iCE-BECs (Figure 5G; Videos S1 and S2). In contrast, the number of sorting tubules was substantially reduced in ApoE4 iCE-BECs (Figure 5H). Together, these data show that ApoE4 impairs endosomal maturation, which is reflected by enlarged, less acidic endosomes with reduced sorting.

**Figure 6:**
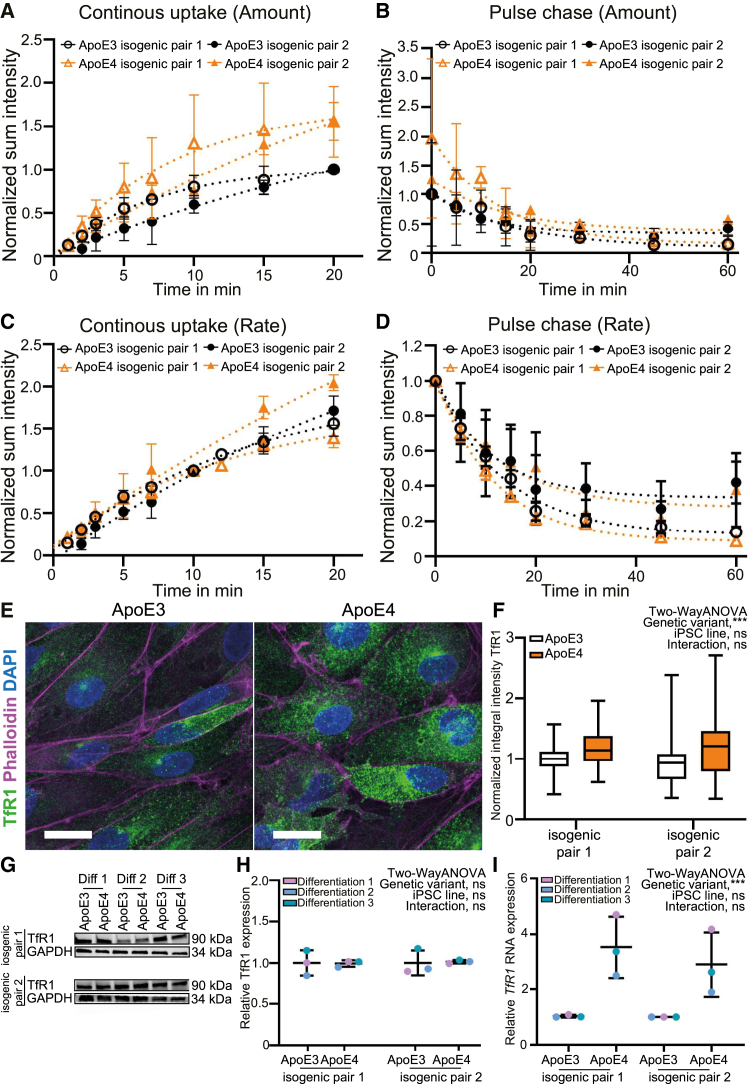
ApoE4 iCE-BECs have increased TfR1 expression but no changes to trafficking kinetics (A–D) Time courses of continuous uptake (A) or pulse-chase (B) assays showing total vesicular intensity of Tf in ApoE3 or ApoE4 iCE-BECs in both isogenic pairs. Intensity values were divided by the mean of the transferrin intensity at 20 min (A) or immediately after the pulse (B) in ApoE3 iCE-BECs, in each isogenic pair. Points show the average, and error bars show the SEM from 50 images per experiment in four independent differentiations for isogenic pair 1 and three independent differentiations for isogenic pair 2 (A) or six independent differentiations for isogenic pair 1 and three independent differentiations for isogenic pair 2 (B). Lines show the best exponential fit for the experimental data. Graphs in (C and D) were normalized to the transferrin intensity at 10 min in each condition in (C) and immediately after the pulse in each condition in (D). (E) Representative maximum intensity projection images of iCE-BECs with ApoE3 or ApoE4 genetic variant showing TfR1 pseudo-colored in green, Phalloidin in magenta, and DAPI-stained nuclei in blue. Scale bars, 20 μm. (F) Quantification of TfR1 integrated intensity in Phalloidin-positive area in both isogenic pairs. Intensity values for each experiment were normalized to ApoE3 conditions. Graph shows boxplots with interquartile ranges and median. Lines show the 5th and 95th percentiles. Significant main effect of genetic variant on TfR1 intensity, ^∗∗∗^*p* < 0.001; no significant main effect of iPSC line on TfR1 intensity; and no significant interaction between iPSC line and genetic variant by two-way ANOVA with *n* = 3 independent differentiations with approximately 200 cells per experiment. (G) Representative immunoblot detecting TfR1 with GAPDH as a loading control showing three independent differentiations (Diff) for the two isogenic pairs. (H) Quantification of relative TfR1 protein expression of immunoblot in (G). Graphs show mean ± SD. Points represent independent differentiations. No significant main effect of genetic variant or iPSC line on TfR1 expression and no significant interaction between iPSC line and genetic variant by two-Way ANOVA with *n* = 3 independent differentiations. (I) Quantification of relative *TfR1* mRNA expression by quantitative PCR. Graph shows mean ± SD. Points represent independent differentiations. ^∗∗∗^*p* < 0.001, no significant main effect of iPSC line on TfR1 intensity and no significant interaction between iPSC line and genetic variant by two-Way ANOVA with *n* = 3 independent differentiations with three technical replicates per experiment from both isogenic pairs.

Video S1. Representative video of transferrin (green) intracellular transport in live iCE-BECs (isogenic pair 1) with ApoE3 gene variant Cells were incubated with fluorescently labeled transferrin for three hours and then videos of one minute were acquired at 100× using a Widefield microscope. Representative image frames of those videos are shown in Figure 5G, related to Figure 5.

Video S2. Representative video of transferrin (green) intracellular transport in live iCE-BECs (isogenic pair 1) with ApoE4 gene variant Cells were incubated with fluorescently labeled transferrin for three hours and then videos of one minute were acquired at 100× using a Widefield microscope. Representative image frames of those videos are shown in Figure 5G, related to Figure 5.

### ApoE4 increases TfR1 expression without altering its transport rate in iCE-BECs

We next asked whether changes in endosome maturation led to functional consequences in intracellular transport rates. To evaluate this, we followed the uptake and recycling of fluorescently labeled Tf in iCE-BECs from both isogenic pairs using a continuous pulse or a pulse-chase experimental design (Figures S5A and S5B). ApoE4 iCE-BECs had a 50% higher total amount of internalized Tf compared to ApoE3 iCE-BECs (Figure 6A). This increase in total internalized Tf was confirmed by flow cytometry (Figure S5C). However, the rate of Tf internalization was similar between ApoE4 and ApoE3 iCE-BECs (Figure 6C). Similarly, the total amount of Tf after a 20-min pulse was higher in ApoE4 compared to ApoE3 iCE-BECs (Figure 6B), but the recycling rate was similar between the two genetic variants (Figure 6D). An increased Tf uptake capacity without changes to the internalization and recycling rates could be explained by the upregulation of TfR1 expression. Indeed, we found that both mRNA and protein TfR1 expression were upregulated in ApoE4 compared to ApoE3 iCE-BECs (Figures 6E–6I). Note that western blots did not reflect increased TfR expression in ApoE4 cells. These data show that ApoE4 leads to TfR1 upregulation but does not alter intracellular transport rates in iCE-BECs.

### ApoE4 alters iron metabolism homeostasis in iCE-BECs

**Figure 7:**
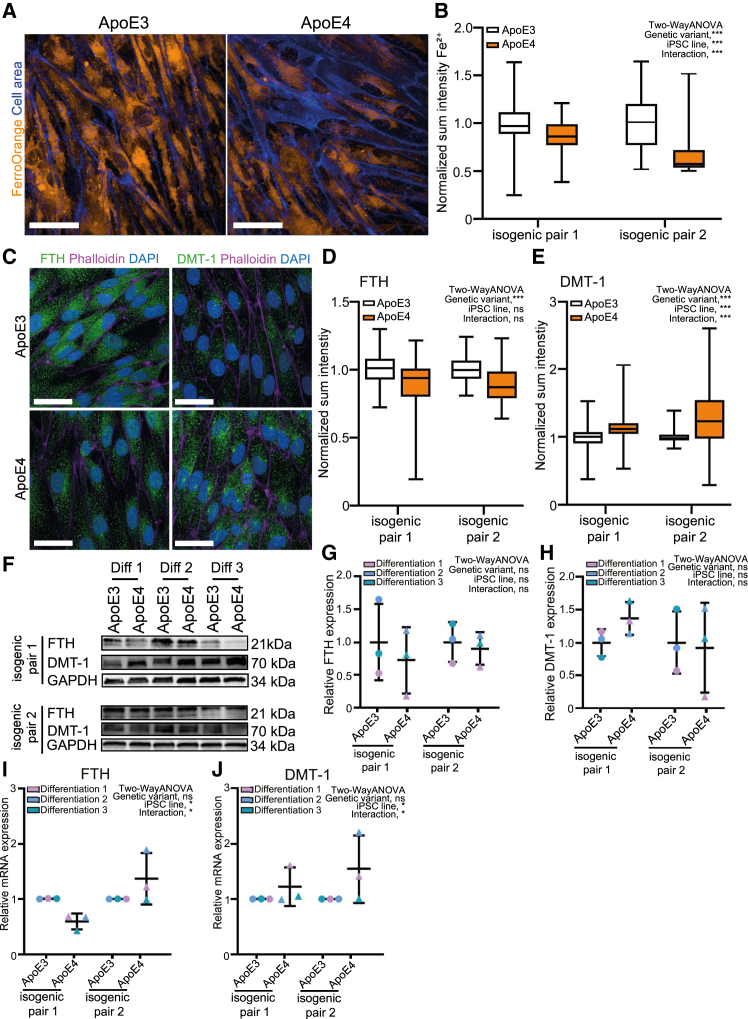
ApoE4 reduces intracellular iron levels and alters iron transport pathways in iCE-BECs (A) Representative maximum intensity projection images of iCE-BECs with ApoE3 or ApoE4 genetic variants after labeling labile iron (II) ions (Fe^2+^) with FerroOrange pseudo-colored in orange and cell area labeled by CytoTrace Green in blue. Scale bars, 50 μm. (B) Quantification of sum intensity of FerroOrange intensity normalized to the total cell area per image. Intensity values were normalized to data from ApoE3 iCE-BECs per experiment. Significant main effect of genetic variant on FerroOrange intensity, ^∗∗∗^*p* < 0.001; significant main effect of iPSC line on FerroOrange intensity, *p* < 0.001; and significant interaction between iPSC line and genetic variant, ^∗∗∗^*p* < 0.001 by two-way ANOVA with *n* = 3 independent differentiations with 40 images per experiment from both isogenic pairs. (C) Representative maximum intensity projection images of iCE-BECs with ApoE3 or ApoE4 genetic variant after immunostaining for FTH and DMT-1 (green) and actin (phalloidin, magenta). DAPI-stained nuclei are shown in blue. Scale bars, 100 μm. (D and E) Quantification of FTH (D) and DMT-1 (E) sum intensity in Phalloidin-positive area. Intensity values were normalized to data from ApoE3 iCE-BECs for each experiment. Significant main effect of genetic variant on FTH intensity, *p* < 0.001; no significant main effect of iPSC line on FTH intensity; and no significant interaction between iPSC line and genetic variant. Significant main effect of genetic variant on DMT-1 intensity, *p* < 0.001; significant main effect of iPSC line on DMT-1 intensity, *p* < 0.001; and significant interaction between iPSC line and genetic variant, *p* < 0.001 by two-way ANOVA with *n* = 3 independent differentiations with approximately 800 cells per experiment from both isogenic pairs. Graphs in (B, D, and E) show boxplots with interquartile ranges and medians. Lines show the 5th and 95th percentiles. (F) Representative immunoblot detecting FTH and DMT-1 with GAPDH as a loading control showing three independent differentiations (Diff) for the two isogenic pairs. (G and H) Quantification of relative FTH (G) and DMT-1 (H) protein expression of immunoblot in (F). Graphs show mean ± SD. Points represent independent differentiations. No significant main effect of genetic variant or iPSC line on FTH or DMT-1 expression and no significant interaction between iPSC line and genetic variant by two-way ANOVA with *n* = 3 independent differentiations. (I and J) Quantification of relative mRNA expression by quantitative PCR of ferritin heavy chain (FTH) and Divalent metal transporter 1 (DMT-1). Graph shows mean ± SD. Points represent independent differentiations. No significant main effect of genetic variant on FTH expression; significant main effect of iPSC line on FTH expression, ^∗^*p* < 0.05; and significant interaction between iPSC line and genetic variant, ^∗^*p* < 0.05; no significant main effects of genetic variant or iPSC line on DMT-1 expression; no interaction between iPSC line and genetic variant by two-way ANOVA with *n* = 3 independent differentiations with three technical replicates per experiment.

TfR1 expression is regulated by iron responsive proteins that sense iron levels in the cytoplasm (Anderson and Frazer 2017). In iron-deficient cells, iron-responsive proteins upregulate TfR1 mRNA by binding to its 3′UTR, thus preventing its degradation. We therefore tested whether ApoE4 iCE-BECs had lower cytosolic iron levels in both isogenic pairs. To measure the intracellular labile iron pool, we used FerroOrange, a fluorescent probe that specifically detects labile iron (II) ions (Fe2^+^) in live cells (Figure S6A). With this method, we found that ApoE4 iCE-BECs had a reduced labile iron pool compared to ApoE3 cells (Figures 7A and 7B). We confirmed this result with an orthogonal method that measures the intracellular iron pool with the metal-sensitive calcein-acetoxymethyl ester (Calcein-AM). This method confirmed a reduction in the labile iron pool in ApoE4 compared to ApoE3 iCE-BECs (Figures S6B–S6D).

The changes in the iron labile pool in iCE-BECs suggest that the effect of ApoE4 broadly impact iron metabolism. Therefore, we evaluated proteins regulated by iron-responsive elements: ferritin (ferritin heavy chain, FTH; iron storage) and divalent metal transporter 1 (DMT-1; iron import). It is well documented that low cytosolic iron downregulates FTH while upregulating DMT-1 (Wallander et al., 2006). We found that ferritin protein and mRNA expression (Figures 7C–7G and 7I) were downregulated, whereas DMT-1 protein and mRNA (Figures 7C–7F, 7H, and 7J) were upregulated in ApoE4 compared to ApoE3 iCE-BECs. Note, however, that the effect size for mRNA and protein via western blot analysis was highly variable between isogenic pairs. This points to a potential effect of the genetic background on intracellular iron levels. Altogether, our data show that ApoE4 alters iron cytoplasmic levels and leads to expression changes across the iron transport pathway in iCE-BECs.

In conclusion, ApoE4 iCE-BECs exhibited impaired maturation of endosomes and disrupted iron homeostasis, characterized by lower intracellular iron levels and altered expression of iron-related genes. These findings highlight the importance of the ApoE4 gene variant in modulating intracellular trafficking and iron metabolism in brain endothelial cells.

## Discussion

Here, we describe a protocol to generate iCE-BECs that shows (1) a brain endothelial transcriptomic and proteomic signature, (2) functional barrier properties, and (3) recapitulate receptor-mediated transcytosis. Importantly, these three features were reproduced across multiple iPSC parental lines. The iCE-BEC protocol shares similarities with the recently described cARLA differentiation method (Porkoláb et al., 2024) and thus confirms the importance of Wnt/cAMP activation and TGF-β inhibition to robustly induce brain endothelial identity and barrier properties of endothelial cells *in vitro*. In comparison with the cARLA differentiation method, iCE-BECs show increased expression of ZIC3 and FOXF2, two transcription factors specifically enriched in brain endothelial cells *in vivo* and important to induce barrier properties in endothelial cells (Patel et al., 2024). Importantly, iCE-BECs showed the same extent of BrS antibody transcytosis observed in a BBB organoid model (Simonneau et al., 2021). We therefore consider iCE-BECs as a suitable model to investigate receptor-mediated transcytosis across the BBB.

The ApoE4 genetic variant is the major risk factor for sporadic AD heavily affecting the onset of the disease (Armstrong 2019). Extensive evidence documents the impact of ApoE4 on BBB permeability and function (Halliday et al., 2016; Montagne et al. 2020, 2021; Yamazaki et al., 2020). However, a recent study using iPSC-derived brain endothelial-like cells found that the effect of ApoE4 on BBB permeability is likely not cell-autonomous (Ding et al., 2024). In this manuscript, we show that ApoE4 expression in brain endothelial cells leads to both impaired endosome maturation and altered iron homeostasis. This finding expands the role of ApoE4 at the BBB beyond paracellular permeability to include cell-autonomous regulation of intracellular transport.

Previous data using ApoE4 knockin mice showed an increase in TfR1 expression in brain endothelial cells (Barisano et al., 2022), similar to the effect that we see in iCE-BECs. Our data suggest that TfR1 upregulation is a consequence of lower cytoplasmic iron levels in ApoE4 cells. We propose a model to explain how impaired endosomal maturation observed in ApoE4 BECs leads to lower cytosolic iron levels (Figure S7). First, ApoE4 triggers increased endosomal pH. Second, increased endosomal pH leads to reduced dissociation of ferric iron from Tf. Third, sustained ferric iron binding to Tf prevents DMT-1-mediated transport into the cytosol. Together, these events would lead to an iron-depleted cytosol and trigger expression changes of iron-related genes, including TfR1.

How ApoE4 leads to changes in early endosome maturation remains to be clarified. Transcriptomic analysis performed on brains of ApoE4 mice revealed a significant upregulation of genes involved in the regulation of endosomal-lysosomal pathway, including Rab5b, Rab7, Snx3, Snx15, Vps4a, Vps24, and Vps29, and suggested an ApoE4-specific trafficking and sorting dysregulation (Nuriel et al., 2017). Interestingly, our data demonstrating increased endosomal pH is opposite to observations in iPSC-derived astrocytes, where the ApoE4 gene variant showed enlarged early endosomes but decreased endosomal pH (Prasad and Rao 2018). The same study found that NHE6 activity could normalize endosomal pH in iPSC-derived astrocytes. Follow-up studies with iCE-BECs could help to dissect the role of NHE6 or other molecular mechanisms on endosomal maturation in the context of ApoE4. Overall, our findings on the influence of ApoE4 on endosomes and TfR1 expression at the BBB will be instrumental in refining strategies that leverage intracellular transport mechanisms for the delivery of therapeutic antibodies to the brain.

### Limitations of the study

Model development and characterization was performed using four iPSC lines from different donors, and the findings on ApoE4 were observed in two isogenic iPSC pairs. Validation of the findings would be strengthened by replicating the findings in iPSC lines from a larger and diverse pool of donors. Ultimately, however, the translatability of the phenotypes described in iCE-BECs requires confirmation in patients.

## Methods

### hiPSC lines

Unless indicated differently in each figure, all experiments were performed differentiating endothelial cells from hiPS_SFC086_03_03 line. hiPS_SFC086_03_03 were established by reprogramming skin fibroblasts with Sendai virus (CytoTune v II kit) at STEMBancc from a female donor. Karyotype analysis was performed by WiCell, and no abnormalities were detected. To assess the reproducibility of the protocol, BIONi010-C13 (European Bank for induced pluripotent stem cells, ebisc.org), Alstem iPS26 (see below), and BIONi037-A (see below) were used. For ApoE experiments, Bioni037-A (16423, homozygous ApoE3 gene variant) and Bioni037-A4 (I40-53, homozygous ApoE4 gene variant), referred as isogenic pair 1, and Alstem line iPS16 (homozygous ApoE4 gene variant) and iPS26 (homozygous ApoE3 gene variant), referred as isogenic pair 2, were used. All cell lines were tested for mycoplasma contamination.

### hiPSC differentiation into iCE-BECs

Human induced pluripotent stem cells (hiPSCs) were maintained under standard culturing conditions using plates coated with Geltrex (A1413301, Thermo Fisher) and mTeSR Plus Media (100-0276, Stemcell). Passaging was performed using Gentle Cell Dissociation Reagent (100-0485, Stemcell). To differentiate iCE-BECs, 2 million hiPSCs were seeded in a 10-cm dish coated with Geltrex in 10 mL of mTeSR Plus Media supplemented with ROCK inhibitor, Y-27632 10 μM (SCM075, EMD Millipore). Twenty-four hours later, the media was replaced with mesodermal induction media composed of DMEM/F12 (31331-028, Gibco) and Neurobasal medium (21103-049, LifeTechnologies) 1:1, 2-Mercaptoethanol (31350-10, Thermo Fisher), B27 (17504044, Gibco), N2 (17502048, Gibco) supplemented with fresh CHIR-99021 8 μM (13122, Cayman), and BMP4 25 ng/mL (120-05ET, PeproTech). On days 4 and 5, media was replaced with endothelial differentiation medium consisting of StemPro-34 SFM Media (10639011, Life Technologies) with StemPro-34 supplement, Glutamax (35050061, Gibco), and Penicillin-Streptomycin (15070063, Gibco) and freshly supplemented with VEGF165 50 ng/mL (293-VE-010, R&D) and Forskolin 2 μM (ab120058, Abcam). On day 6 of culture, cells were replated in 10 cm dishes (1.2 million per dish) coated with vitronectin 2.5 μg/mL (SRP3186, Sigma) and fibronectin 7.5 μg/mL (F0895, Sigma) in BBB Identity Acquisition media consisting of VascuLife VEGF Endothelial Medium (LL-0003, Lifeline Cell Technology) supplemented with iCell endothelial cells medium supplement (M1019, FujiFilm Cellular Dynamics) instead of the FBS included in the LL-0003 kit and 10 mL of L-Glutamine LifeFactor instead of the 25 mL included in the kit. The media was freshly supplemented with CHIR-99021 4 μM (13122, Cayman), SB-431542 5 μM (72234, Stemcell), and cAMP 50 nM (ab120424, Abcam).

Cells were cultured in these conditions until day 11, changing media every 2 or 3 days. On day 11 of culture, PECAM1-positive cells were MACS-sorted according to the manufacturer’s protocol using CD31 MicroBead Kit from MACS Miltenyi Biotec (130-091-935, Miltenyi Biotec). After MACS, cells were either frozen in liquid nitrogen or replated in the same conditions with BBB identity maintenance media (same composition of BBB Identity Acquisition media). For all experiments performed with iCE-BECs, plates and dishes were coated with vitronectin and fibronectin prior to cell seeding, and cells were cultured in BBB identity maintenance media.

For iEC and iEC-Rep comparison (schematic in Figure 1A), on day 6, progenitor cells were replated on fibronectin-coated dishes and cultured in expansion media. Expansion media consisted of VascuLife VEGF Endothelial Medium (LL-0003, Lifeline Cell Technology) supplemented with iCell endothelial cells medium supplement (M1019, FujiFilm Cellular Dynamics) instead of the FBS included in the kit and 10 mL of L-Glutamine LifeFactor instead of the 25 mL included in the kit (without CHIR-99021, SB-431542, and cAMP). After MACS on day 11, iECs were cultured in the same conditions (maintenance media). For the iEC-Rep condition, the media was supplemented with RepSox 10 μM (73794, Stemcell) 48 h before performing the analysis.

Experiments were carried out on day 14 (3 days after MACS sorting) and with iCE-BECs differentiated from the hiPS_SFC086_03_03 parental line, if not indicated differently.

### Statistical analysis

Statistical analyses were performed using GraphPad Prism 10.2.2 software. Normality of the data was evaluated by the Shapiro-Wilk test. For normally distributed numeric data, two-way ANOVA was used to evaluate the effects of genotype and parental cell line in experimental readouts, one-way ANOVA to compare across multiple groups, and Student’s t test for comparisons between two groups. For datasets not distributed normally, non-parametric Kruskal-Wallis test was used to compare between multiple groups or Mann-Whitney U test was used for comparisons between two groups. The details for each statistical test are indicated in each figure legend. Data are reported as mean ± SD.

## Resource availability

### Lead contact

Requests for further information or more detailed protocols should be directed to and will be fulfilled by the corresponding author, Roberto Villaseñor (roberto.villasenor_solorio@roche.com).

### Material availability

This study did not generate new unique reagents.

### Data and code availability

• The mass spectrometry proteomics data have been deposited to the ProteomeXchange Consortium via the PRIDE partner repository with the dataset identifier PXD062808.
• The data discussed in this publication have been deposited in NCBI’s Gene Expression Omnibus and are accessible through GEO Series accession number GSE280214 (scRNA seq across conditions), GSE296358 (bulk RNA-seq across conditions), and GSE296377 (bulk RNA-seq across parental lines and passages).

## Acknowledgments

We thank Dr. Sybille Seiler, Dr. Urs Langen, and Dr. Colette Bichsel for scientific discussions and suggestions. We would like to thank Giacomo Valsecchi, Lena Jutz, Petra Stäuble, Telma Lopes, Lorena Fabella, Dr. Heloise Ragelle, Alena Spielmann, and Pamela Strassburger for excellent technical support. We would like to thank Dr. Udo Hetzel and Barbara Prähauser for their support with the transmission electron microscopy. This collaboration project is co-funded by the PPP Allowance made available by Health-Holland and Top Sector Life Sciences & Health through the PPP program Brains (by 10.13039/501100010969Alzheimer Nederland, EpilepsieNL, 10.13039/501100008358Hersenstichting Nederland, and MS Research), to stimulate public-private partnerships (IMM-BBB, PPS-BR-2023-01).

## Author contributions

L.B. contributed to project administration, conceptualization, data curation, methodology, investigation, visualization, writing—original draft, and writing—review & editing.

N.S.-R. contributed to conceptualization, supervision, and writing—review & editing.

C.S. contributed to conceptualization, supervision, data curation, methodology, investigation, and writing—review & editing. S.C., S.R., A.R., T.M., A.A., B.H., L.D.A., K.S., D.V.T., J.F.-P., N.R.W., X.M.S., C.Zanini., C.Zundel., and S.G. contributed to data curation, methodology, investigation, and writing—review & editing.

L.F. contributed to conceptualization, writing—review & editing, A.O. contributed to writing—review & editing, M.P. contributed to project administration, supervision, conceptualization, data curation, methodology, investigation, visualization, writing—original draft, and writing—review & editing and decision to submit, R.V. contributed to project administration, supervision, conceptualization, data curation, methodology, visualization, writing—original draft, and writing—review & editing and decision to submit.

## Declaration of interests

L.B., N.S.-R., C.S., A.A., S.C., S.R., A.R., T.M., B.H., L.D.A., J.F.-P., K.S., D.V.T., C.Zanini., C.Zundel., L.F., S.G., M.P., and R.V. were employees and shareholders of F. Hoffmann-La Roche Ltd at the time the work was completed. X.M.S. and N.R.W. are employees of MIMETAS BV. OrganoPlate, OrganoReady, OrganoFlow, and OrganoTEER are registered trademarks of MIMETAS BV.

## Supplemental information

Document S1. Figures S1–S7, Tables S1 and S3–S5, and supplemental methods

Table S2. The top 100 most variable genes contributing to each Principal Component in the analysis shown in Figure 2H iCE-BECs generated from three different iPS lines (SFC086_03_03, Bioni037A, Bioni10C) and across passages were compared. The top 100 most variable genes contributing to each Principal Component in the analysis shown in Figure 2H. iCE-BECs generated from three different iPS lines (SFC086_03_03 line, Bioni037A, Bioni10C) and across passages were compared.

Document S2. Article plus supplemental information

## References

[bib1] Abbott N.J. (2013). Blood-brain barrier structure and function and the challenges for CNS drug delivery. J. Inherit. Metab. Dis..

[bib2] Anderson G.J., Frazer D.M. (2017). Current understanding of iron homeostasis. Am. J. Clin. Nutr..

[bib3] Armstrong A.R. (2019). Risk factors for Alzheimer's disease. Folia Neuropathol..

[bib4] Ayloo S., Lazo C.G., Sun S., Zhang W., Cui B., Gu C. (2022). Pericyte-to-endothelial cell signaling via vitronectin-integrin regulates blood-CNS barrier. Neuron.

[bib5] Barisano G., Kisler K., Wilkinson B., Nikolakopoulou A.M., Sagare A.P., Wang Y., Gilliam W., Huuskonen M.T., Hung S.-T., Ichida J.K. (2022). A “multi-omics” analysis of blood–brain barrier and synaptic dysfunction in APOE4 mice. J. Exp. Med..

[bib6] Bien-Ly N., Yu Y.J., Bumbaca D., Elstrott J., Boswell C.A., Zhang Y., Luk W., Lu Y., Dennis M.S., Weimer R.M. (2014). Transferrin receptor (TfR) trafficking determines brain uptake of TfR antibody affinity variants. J. Exp. Med..

[bib7] Blanchard J.W., Bula M., Davila-Velderrain J., Akay L.A., Zhu L., Frank A., Victor M.B., Bonner J.M., Mathys H., Lin Y.-T. (2020). Reconstruction of the human blood–brain barrier in vitro reveals a pathogenic mechanism of APOE4 in pericytes. Nat. Med..

[bib8] Blumenfeld J., Yip O., Kim M.J., Huang Y. (2024). Cell type-specific roles of APOE4 in Alzheimer disease. Nat. Rev. Neurosci..

[bib9] Ding Y., Palecek S.P., Shusta E.V. (2024). iPSC-derived blood-brain barrier modeling reveals APOE isoform-dependent interactions with amyloid beta. Fluids Barriers CNS.

[bib10] Grimm H.P., Schumacher V., Schäfer M., Imhof-Jung S., Freskgård P.O., Brady K., Hofmann C., Rüger P., Schlothauer T., Göpfert U. (2023). Delivery of the Brainshuttle™ amyloid-beta antibody fusion trontinemab to non-human primate brain and projected efficacious dose regimens in humans. mAbs.

[bib11] Halliday M.R., Rege S.V., Ma Q., Zhao Z., Miller C.A., Winkler E.A., Zlokovic B.V. (2016). Accelerated pericyte degeneration and blood–brain barrier breakdown in apolipoprotein E4 carriers with Alzheimer’s disease. J. Cereb. Blood Flow Metab..

[bib12] Lu T.M., Houghton S., Magdeldin T., Durán J.G.B., Minotti A.P., Snead A., Sproul A., Nguyen D.-H.T., Xiang J., Fine H.A. (2021). Pluripotent stem cell-derived epithelium misidentified as brain microvascular endothelium requires ETS factors to acquire vascular fate. Proc. Natl. Acad. Sci. USA.

[bib13] Montagne A., Nation D.A., Sagare A.P., Barisano G., Sweeney M.D., Chakhoyan A., Pachicano M., Joe E., Nelson A.R., D'Orazio L.M. (2020). APOE4 leads to blood-brain barrier dysfunction predicting cognitive decline. Nature.

[bib14] Montagne A., Nikolakopoulou A.M., Huuskonen M.T., Sagare A.P., Lawson E.J., Lazic D., Rege S.V., Grond A., Zuniga E., Barnes S.R. (2021). APOE4 accelerates advanced-stage vascular and neurodegenerative disorder in old Alzheimer’s mice via cyclophilin A independently of amyloid-β. Nat. Aging.

[bib15] Nuriel T., Peng K.Y., Ashok A., Dillman A.A., Figueroa H.Y., Apuzzo J., Ambat J., Levy E., Cookson M.R., Mathews P.M., Duff K.E. (2017). The Endosomal–Lysosomal Pathway Is Dysregulated by APOE4 Expression in Vivo. Front. Neurosci..

[bib16] Patel R., Cui A., Bosco P., Akcan U., Richters E., Delgado P.B., Agalliu D., Sproul A.A. (2024). Generation of hiPSC-derived brain microvascular endothelial cells using a combination of directed differentiation and transcriptional reprogramming strategies. bioRxiv.

[bib17] Patsch C., Challet-Meylan L., Thoma E.C., Urich E., Heckel T., O'Sullivan J.F., Grainger S.J., Kapp F.G., Sun L., Christensen K. (2015). Generation of vascular endothelial and smooth muscle cells from human pluripotent stem cells. Nat. Cell Biol..

[bib18] Porkoláb G., Mészáros M., Szecskó A., Vigh J.P., Walter F.R., Figueiredo R., Kálomista I., Hoyk Z., Vizsnyiczai G., Gróf I. (2024). Synergistic induction of blood–brain barrier properties. Proc. Natl. Acad. Sci. USA.

[bib19] Prasad H., Rao R. (2018). Amyloid clearance defect in ApoE4 astrocytes is reversed by epigenetic correction of endosomal pH. Proc. Natl. Acad. Sci. USA.

[bib20] Roudnicky F., Zhang J.D., Kim B.K., Pandya N.J., Lan Y., Sach-Peltason L., Ragelle H., Strassburger P., Gruener S., Lazendic M. (2020). Inducers of the endothelial cell barrier identified through chemogenomic screening in genome-edited hPSC-endothelial cells. Proc. Natl. Acad. Sci. USA.

[bib21] Sabbagh M.F., Heng J.S., Luo C., Castanon R.G., Nery J.R., Rattner A., Goff L.A., Ecker J.R., Nathans J. (2018). Transcriptional and epigenomic landscapes of CNS and non-CNS vascular endothelial cells. eLife.

[bib22] Simonneau C., Duschmalé M., Gavrilov A., Brandenberg N., Hoehnel S., Ceroni C., Lassalle E., Kassianidou E., Knoetgen H., Niewoehner J., Villaseñor R. (2021). Investigating receptor-mediated antibody transcytosis using blood-brain barrier organoid arrays. Fluids Barriers CNS.

[bib23] Villaseñor R., Lampe J., Schwaninger M., Collin L. (2019). Intracellular transport and regulation of transcytosis across the blood–brain barrier. Cell. Mol. Life Sci..

[bib24] Villaseñor R., Schilling M., Sundaresan J., Lutz Y., Collin L. (2017). Sorting Tubules Regulate Blood-Brain Barrier Transcytosis. Cell Rep..

[bib25] Wallander M.L., Leibold E.A., Eisenstein R.S. (2006). Molecular control of vertebrate iron homeostasis by iron regulatory proteins. Biochim. Biophys. Acta.

[bib26] Wevers N.R., Kasi D.G., Gray T., Wilschut K.J., Smith B., van Vught R., Shimizu F., Sano Y., Kanda T., Marsh G. (2018). A perfused human blood-brain barrier on-a-chip for high-throughput assessment of barrier function and antibody transport. Fluids Barriers CNS.

[bib27] Yamazaki Y., Shinohara M., Yamazaki A., Ren Y., Asmann Y.W., Kanekiyo T., Bu G. (2020). ApoE (Apolipoprotein E) in Brain Pericytes Regulates Endothelial Function in an Isoform-Dependent Manner by Modulating Basement Membrane Components. Arterioscler. Thromb. Vasc. Biol..

[bib28] Yang A.C., Vest R.T., Kern F., Lee D.P., Agam M., Maat C.A., Losada P.M., Chen M.B., Schaum N., Khoury N. (2022). A human brain vascular atlas reveals diverse mediators of Alzheimer’s risk. Nature.

