# Supplementary material for: ApoE4 disrupts intracellular trafficking and iron homeostasis in a reproducible iPSC-based model of human brain endothelial cells
Source: Stem Cell Reports. 2025 Aug 21;20(9):102607. doi: 10.1016/j.stemcr.2025.102607 (PMC12447338; doi:10.1016/j.stemcr.2025.102607)
Supplement: Document S1. Figures S1–S7, Tables S1 and S3–S5, and supplemental methods [file mmc1.pdf]

Stem Cell Reports, Volume *20*

Supplemental Information

ApoE4 disrupts intracellular trafficking and iron homeostasis in a reproducible iPSC-based model of human brain endothelial cells

Luisa Bell, Shane Clerkin, Sila Rizalar, Antoine Rizkallah, Nadine Stokar-Regenscheit, Xandor M. Spijkers, Nienke R. Wevers, Claire Simonneau, Angélique Augustin, Barbara Höllbacher, Lia D'Abate, Joanna Ficek-Pascual, Kim Schneider, Desiree Von Tell, Thomas Maurissen, Chiara Zanini, Christelle Zundel, Sabrina Golling, Christine Becker, Alex Odermatt, Lynette C. Foo, Martina Pigoni, and Roberto Villaseñor

Supplementary Material

Supplementary Table 1. List of genes for benchmarking iCE-BECs towards endothelial transcriptomic signature. List adapted from Lu, Houghton, Magdeldin, Durán, Minotti, Snead, Sproul, Nguyen, Xiang, Fine, Rosenwaks, Studer, Rafii, Agalliu, Redmond, Lis [1].

<table>
<tr><th colspan="9">High PC1 Loading</th></tr>
<tr>
<td>PECAM1
CD93
MMP1
MMRN1
CLEC14A
ROBO4
SRGN
GIMAP4
GIMAP6
DIPK2B
ERG
VWF
LAMA4
CLDN5
CAVIN2
STAB1
GIMAP8
EMCN
CD34
ICAM2
BCL6B
BMX
FAM124B
LYVE1
ACVRL1
ESAM
TIE1
APLN
PPP1R16B
SHE
ANPEP
ARHGEF15
HHEX
TAL1
PTX3
RHOJ
LINC01235
SOX18
MFNG
GPR4
ADGRF5
PCAT19
GIMAP7
IL1RL1</td>
<td>FABP4
PALMD
ZEB1
MMRN2
GIMAP1
CNRIP1
ESM1
TM4SF18
GIMAP2
HHIP
PLVAP
ADGRL4
ABI3
TNFRSF14
APOL3
LAPTM5
PCDH12
ECSCR
ENG
SOX17
TNFSF10
GNG11
CALCRL
TM4SF1
S1PR1
LYL1
BGN
TMEM173
CLEC2B
ANGPT2
SH2D3C
DOCK10
LINC01013
PTPRB
KLHL6
FAM107A
TLR4
MEG3
EVA1C
SELP
TNFAIP8L3
NOVA2
KDR
CARD6</td>
<td>MYRIP
CXCL8
ITGB3
MANCR
CXCR4
DLL4
PDE4B
FAM43A
PDE7B
RASGRP3
PIK3CG
CLDN11
LNCOG
NRN1
IL33
IFI44
KLF2
CARD16
MGP
INSYN2B
SGIP1
FERMT3
HCLS1
TMEM204
BMP6
SPAAR
GMFG
LDB2
NOTCH4
TMEM255B
CDH5
RASIP1
CHST1
SCARF1
CYTL1
FGD5
FOXC2
TMEM273
P4HA3
FLI1
SH3TC1
LGALS1
TDRD10
RAC2</td>
<td>WDFY4
FAM78A
NPAS2
GBP4
NRGN
ADAMTSL1
C2CD4B
MAPK11
HLX
HOXD8
GRAP
NFIB
CPNE5
GRASP
ARHGEF28
MALL
LY96
GJA4
DKK1
STEAP1B
THSD1
LIX1L
CDYL2
FLT4
GGT5
C22orf34
NFIA
PREX1
HOXA9
MLKL
COL8A1
SEMA6B
SYNPO
TFEC
COL13A1
PLCL1
CASP1
HTR2B
ERAP2
CCRL2
TOX2
LINC01116
THBS1
PLSCR4</td>
<td>IL15RA
CRIP2
PDE2A
IRAK3
ITGA10
GAB3
ADCY4
LINC01358
ACE
CD163L1
FRMD3
SEMA3G
LONRF3
CFAP54
MYCT1
THBD
CARD8-AS1
CHCHD2
CXCL1
SHANK3
FLT1
NLRC5
CASP4
NOS3
EGFL7
SAMD9
VAMP5
PLXNA4
MILR1
CPT1A
CAV1
KANK3
ZNF366
LRRC70
SLFN11
SERPIND1
SAMSN1
KCTD12
ANXA2R
VEPH1
LINC02454
CTSS
LOX
TBX18</td>
<td>STK32B
MEOX2
SPOCK1
RNASE1
HOXA10
BACE2
TCTEX1D1
TNS2
LMO2
NEGR1
SERPINE1
SNED1
RGCC
HOXA11
TNFSF4
FAM241A
CNTNAP3B
LHX6
AFAP1L1
MPP4
MLIP
SENCR
GNGT2
ZFYVE28
NFIC
EMP1
PARVB
NTSR1
IFI27
HAGLR
ANKRD55
SH2B3
CD109
CEACAM21
ZEB2
NTN4
EVI2B
PREX2
LINC00520
TNFRSF11A
NFIA-AS2
ABLIM3</td>
<td>SYNE3
EFEMP1
WSCD1
EDN1
PKD1L1
PALD1
IL18R1
VIM
PROCR
COX7A1
IL3RA
CLEC1A
PCSK1
NAV3
INKA1
CCL2
HIC1
RAMP2
NT5E
TMEM156
HHIP-AS1
TM6SF1
MIR137HG
NPR1
HOXD9
DDR2
STX11
PTPRE
SH3RF3
MEF2C
DMTN
STC1
MCTP1
LGALS9
GBP1
LRRC8C
MIR217HG
ARHGAP20
KIAA1549L
GBP2
MT2A
UBA7
TGFBR2</td>
<td>TSPO
AHNAK2
NRG3
SLC9A3R2
ALDH1A2
ULBP2
PAPSS2
IFIT2
TNFRSF1B
MTSS1
VASH1
SORBS2
A2M
FAM124A
KLF9
NRG1
SSTR1
TLE2
MEDAG
MIR155HG
SLC17A9
PLA2G4C
GIPC3
IL7R
SLC43A1
PCDH10
APOLD1
SYT11
ZNF469
TLL1
LINC01094
FOXF1
ST8SIA4
UAP1L1
ENTPD1
FAM155A
USHBP1
ARHGAP22
ITGA5
RFLNB
LPAR6
CUBN
ARAP3</td>
<td>PGF
NID1
PARP12
PMP22
ST6GALNAC4
LINC01197
TEK
SP100
PLXND1
IFIT3
NRIP3
HRH1
ECE1
SH3TC2
DYRK3
SYNM
LTBP2
DPYD
CDKN2C
HOXD1
SPESP1
STEAP1
CLDN14
ADGRA2
EBF1
ITGA11
MSRB3
CCNA1
RFTN2
ARHGEF6
MAMLD1
TBXA2R
IL4I1
GAPLINC
HSD17B2
RAPGEF5
IL6R
OAF
ARHGAP24
NOX4</td>
</tr>
</table>

					KCNN3<br>DYSF	F2RL2	CSGALNACT1	
**Low PC1 loading**								
**CLDN6**<br>**GPC3**<br>**LIN28A**<br>**PTN**<br>**DSG2**<br>**IGDCC3**<br>**CRABP2**<br>**DSC2**<br>**AP1M2**<br>**EPCAM**<br>**DMKN**<br>**RBM47**<br>**ACTC1**<br>**QPRT**<br>**SPP1**<br>**GPC6**<br>**SLC1A3**<br>**TRIM71**<br>**LIN28B**<br>**MSX2**<br>**ENPEP**<br>**COL1A1**	SERPING1<br>ZFP42<br>MMP9<br>NKAIN4<br>WFDC2<br>LCP1<br>ROR2<br>ID4<br>CRYBG2<br>HPGD<br>WDR86<br>SOX9<br>LYPD6B<br>DSP<br>APOE<br>DPPA4<br>EFS<br>H2AFY2<br>CD24<br>H19<br>FBLN1<br>EMB	NPPB<br>GPC4<br>MARVELD3<br>TINCR<br>TNC<br>SYTL1<br>KRT8<br>PDPN<br>NLGN4X<br>IGFBP5<br>SHANK2<br>GPR87<br>MFAP5<br>ARSI<br>TRIM55<br>LRRN4<br>RUBCNL<br>PKIB<br>PRSS16<br>GYG2<br>PURPL<br>CHPF	ACSS3<br>ADAMTS19<br>CTSV<br>RARRES2<br>SPINT2<br>CDH8<br>TFAP2A<br>ERVH48-1<br>SLC27A6<br>IGF2<br>MLPH<br>MYO5B<br>WDR86-AS1<br>TENM3-AS1<br>NSUN7<br>MEIS3<br>MYL7<br>PARP8<br>HAPLN1<br>WNT5B<br>OVOL2<br>CCDC144NL-AS1	NEO1<br>PCDH11X<br>SORL1<br>CPVL<br>MXRA8<br>C4orf19<br>LINC02381<br>HAS2<br>PROM1<br>LEF1<br>CADM1<br>GPRC5C<br>SALL4<br>FAM169A<br>MOB3B<br>CCDC8<br>UNC5C<br>SH2D4A<br>GRIP1<br>RIPOR2<br>DACT1<br>ALPK2	SEMA5A<br>RAMP1<br>GLI2<br>NRK<br>CAMK1G<br>CDX2<br>LUM<br>ALPL<br>PCDHA12<br>JPH2<br>IGFBP3<br>SOX11<br>PCSK1N<br>PKP2<br>TMEM92<br>XKR4<br>PPP2R2B<br>LINC01224<br>CNN1<br>SLC4A4<br>TEAD3<br>TRMT9B	DSC3<br>GALNT17<br>MPPED2<br>IGF2-AS<br>ADGRV1<br>CA2<br>DIO3OS<br>TNFRSF19<br>GAS7<br>GALNT3<br>ERP27<br>KRT19<br>EPS8L2<br>NR6A1<br>NTRK2<br>WWC1<br>PLBD1<br>PDGFRB<br>LMOD1<br>RGS16<br>RIMS2<br>SSC4D	PRKCZ<br>FERMT1<br>ST6GAL2<br>OXTR<br>NSG1<br>PCAT14<br>LRRTM4<br>DCDC2<br>STXBP6<br>CA3<br>SERPINF1<br>PLD5<br>IRS1<br>SRGAP3<br>CHMP4C<br>LSR<br>AFAP1L2<br>ADAMTS2<br>CLDN10<br>BOC<br>LINGO1<br>SCG3	COL4A6<br>COL1A2<br>FUT9<br>ADCY10<br>NPFFR2<br>L1CAM<br>PKDCC<br>TBX3<br>FLRT3<br>LINC00648<br>IGDCC4<br>DLK1<br>CORO2A<br>EDNRA<br>PLPPR3<br>PATJ<br>SALL1

Extraction of the top 500 genes with strongest positive and 500 genes with strongest negative contribution to PC1 from sheet "Fig.1C PC1 Loading Genes" from dataset S02 of the meta-analysis from Lu, Houghton, Magdeldin, Durán, Minotti, Snead, Sproul, Nguyen, Xiang, Fine, Rosenwaks, Studer, Rafii, Agalliu, Redmond, Lis [1]. Subset of extracted genes that were expressed in our cells (392 for high PC1 loading, 193 for low PC1 loading) are listed below. Genes with high PC1 loading are associated with endothelial signature, while low PC1 loading are related to epithelial identity, see Figure 1D.

Supplementary Table 2. The top 100 most variable genes contributing to each Principal Component in the analysis shown in Figure 2H. iCE-BECs generated from three different iPS lines (SFC086_03_03 line, Bioni037A, Bioni10C) and across passages were compared.

The top 100 most variable genes contributing to each Principal Component in the analysis shown in Figure 2H. iCE-BECs generated from three different iPS lines (SFC086_03_03 line, Bioni037A, Bioni10C) and across passages were compared.

Supplementary Materials and Methods

Generation details for ApoE4 lines

For the Bioni037 line, the parental homozygous ApoE3 line was generated from the fibroblasts of a healthy individual with homozygous ApoE3 genetic variant .The isogenic ApoE4 homozygous line was derived from the parental line by editing of the ApoE allele from T/T to C/C at rs429358, which changes aa from Cys112 to Arg112; the genetic variant of base position described by rs7412 in both parent and subclone is C/C, which is an Arg. Together, these define the ApoE4/E4 genetic variant in this subclone. Editing was confirmed and cells were characterized (sequencing, expression of pluripotency markers, morphology) by the European Bank for induced pluripotent Stem Cells (ebisc.org). The parental Alstem line iPS16 was reprogrammed from one single iPSC clone of human bone marrow CD34-positive mononuclear cells. The isogenic control line carrying ApoE3 (iPS26, Alstem) was derived from the parental Alstem line by changing Arg112 to Cys112 in the ApoE allele. Editing was confirmed and cells were characterized (sequencing, expression of pluripotency markers, morphology) by Alstem (alstembio.com).

Single cell RNAseq sequencing and analysis

300,000 cells differentiated with the iEC, iEC-Rep and iCE-BEC protocol (see Figure 1A) were plated in 6 well plates at day 11 and grown at confluence to day 14. On day 14, cells were detached and resuspended into a single cell suspension concentrated $1x10^6$ cells per mL. Cells were then processed following the Chromium Next GEM Single Cell 3' v3.1 protocol for GEM generation and barcoding followed by gene expression library construction according to the manufacturer's instructions. Targeted cell recovery of 10'000 per sample was performed. Dual indexed libraries were sequenced on the Novaseq 6000 with a target sequencing depth of 50'000 single reads per cell. Raw reads were processed with Cell Ranger software (version 7.1.0) and aligned to hg38 reference transcriptome. Count matrices generated by Cell Ranger were corrected for ambient RNA using CellBender 0.2.0 with the expected cells parsed from the Cell Ranger web summary. The number of total droplets to

include was set to 25000, the false positive rate set to 0.01 and the algorithm was trained for 150 epochs. CellBender corrected count matrices were then concatenated and further processed with scanpy (v1.9.3). Barcodes were filtered for observations that CellBender had assigned a latent cell probability greater than 0.5. Furthermore, we excluded low quality cells and potential duplicates by retaining cells with percentage of mitochondrial counts < 5% and with a number of genes between 200 and 2500. Genes detected in less than 30 cells were removed from downstream analysis. Counts were normalized by library size and log1p transformed before determining highly variable genes and regressing out the effect of library size and percentage of mitochondrial counts. Top 10 principal components were used as input for the neighborhood graph which in turn was used to create the UMAP representation.
We utilized the meta-analysis published by Liu and colleagues [1] to generate gene sets associated with an endothelial transcriptomic signature (high PC1 loading) or an epithelial transcriptomic identity (low PC1 loading). In detail, we extracted the top 500 genes with strongest positive and 500 genes with strongest negative contribution to PC1 from sheet "Fig.2C PC1 Loading Genes" from dataset S02 provided as supporting information to their publication (see Supplementary Table 1). We subset their genes to those expressed in our data (392 for high PC1 loading, 193 for low PC1 loading) and visualized the score for the two gene sets through the Seurat (v5.0.1) function AddModuleScore.
We used the scanpy score_genes function to compute per cell scores of the brain endothelial gene set [*CLDN5, MFSD2A, SLC16A1, SLC3A2, SLC38A5, SLC7A5, SLC2A1*]. We then focused on endothelial cells (defined as transcript expression > 0 for at least one of the general endothelial markers [*KDR, VWF, PECAM1, ENG, CDH5, FLT4, FCGR*] and investigated the brain endothelial scores grouped by differentiation protocol.

Bulk RNA sequencing

300,000 cells differentiated with the iEC, iEC-Rep or iCE-BEC protocol (see Figure 1A) were plated at day 11 and grown at confluence to day 14. Differentiation protocols were tested in triplicate, yielding a total of 9 samples (three independent differentiations per condition) for

sequencing. On day 14, cells were collected, and pellets were snap frozen. RNA was extracted and preparation of Illumina stranded TruSeq RNA libraries, including poly(A) enrichment, was performed (2*100 bp paired-end reads). Samples were sequenced on the Illumina NovaSeq and NextSeq by Microsynth. Base calling was conducted using the BCL to FASTQ file converter bcl2fastq2 version 2.20.0 (Illumina). Quality assessment of FASTQ files was performed with FastQC version 0.12.1 (Andrews et al. 2010). Paired-end reads were aligned to the human genome (build "hg38") using the STAR read aligner version 2.7.11b with default mapping parameters (Dobin et al. 2013). Alignment metrics were determined using Picard version 3.1.1 (Broad Institute). Quality of read sequences and alignments was assessed with MultiQC version 1.21 [2]. The number of reads mapped to all RefSeq transcript variants of a gene were combined into a single count value (i.e., read count) assuming a reverse-stranded library, using featureCounts version 2.0.6 [3]. Read counts for 43,294 RefSeq transcripts were generated for all 9 samples. All samples passed quality control checks. Read count normalization, principal component analysis (PCA) and gene ontology (GO) term enrichment were subsequently conducted in R (version 4.3.0). Read count normalization was performed using the edgeR package (version 4.0.5; [4]). Library size and composition were adjusted for using Trimmed mean of M (TMM)-normalized CPM values. Only samples with >=1 CPM and >10 read counts in at least 3 samples were retained. Following gene filtering, a total of 13,536 of 43,294 transcripts were identified as expressed (31%). The PCA was computed based on the top 500 most variable expressed genes using TMM-normalized log2(CPM+1) values. Eigenvalues were extracted and the cumulative percentage of each principal component's (PC) variance explained. PC1 contributed most to sample variance (82.65%), with PC2-4 individually contributing <7.3% each. GO term enrichment was performed on the top 100 loading genes for PC1 using the clusterProfiler package in R (version 4.10.0). Multiple testing correction was performed using the Benjamini-Hochberg method.

Bulk RNA sequencing across lines and passages

iCE-BECs were differentiated using the protocol aforehand described (Figure 1A) starting from 3 parental iPSC lines (hiPS_SFC086_03_03, BIONi010-C13 and Bioni037-A). At least 300,000 cells were collected on day 14 (“Passage 0”). To evaluate the effect of cell passages on cell identity, hiPS_SFC086_03_03 at day 14 were frozen, thawed and subsequently passaged for 3 consecutive times (“Passage 1-3”). The preprocessing was performed in the same way as described above, for the 8 samples. Following gene filtering, a total of 12,887 of 43,294 transcripts were identified as expressed (30%). The PCA was computed based on the top 500 most variable expressed genes using TMM-normalized log2(CPM+1) values. Eigenvalues were extracted and the cumulative percentage of each principal component’s (PC) variance explained. PC1 contributed most to sample variance (67.65%), with PC2-4 individually contributing <15% each. Loadings for all genes were extracted, and the top 100 genes based on absolute loading scores contributing to PC1 and PC2 were examined for the presence of general and brain endothelial markers.

FITC-dextran Permeability in Transwell

50,000 cells were plated at day 11 on the insert of transwell chambers (734-4072, Avantor). Media was changed at day 12 and FITC-dextran permeability experiments were run at day 14 in culture. Briefly, the inserts were moved to a new Receiver Tray supplemented with 600 µL of fresh media. A FITC-dextran (3.3 kDa, D3305; 40 kDa, D1845; 70 kDa D1822, all purchased from ThermoFisher) dilution of 50 µg/ml was added in the insert compartment and plates were incubated for 30 minutes at 37 °C. After incubation, 100 µL of the media from each Receiver Tray were transferred to wells of a black 96-well opaque plate (PBK96G-1.5-F, MatTek USA) for fluorescence measurement. Fluorescence was read at 485 nm and 535 nm excitation and emission, respectively and apparent permeability (P_{app}) calculated using the formula:

$$P\ app = (dQ/dt) \times 1/AC$$

where dQ/dt is change in concentration / change in time, A is the growth area in the insert and C is the initial concentration in the insert chamber.

Histology of transwell

iCE-BECs were seeded on a transwell (734-4072, Avantor) as described above and fixed at day 14 in 4% PFA for

30 min, subsequently washed with PBS for three times. Cells including the transwell mesh were embedded in 2% Agarose (V3121, Promega), dehydrated overnight (TissueTek VIP5, Sakura) and ultimately embedded vertically in paraffin using an embedding console (Tissue-Tek® TEC™5, Sakura). Sagittal microtome sections at 4 um were prepared on Superforst Plus glass slides (J1800AMNZ, ThermoFisher) and an automated Haematoxylin & Eosin staining was performed (Ventana HE600, Roche). Slides were imaged with a whole slide scanner at 40 × (Hamamatsu, NanoZoomer S360, standard HE).

Transendothelial electrical resistance (TEER) assessment

iCE-BECs and iECs were seeded into transwells (3470, Corning) on day 11 at a density of 120,000 cells per 6.5 mm insert and maintained in their respective maintenance media to form monolayers. On day 14, inserts were transferred to the wells of a cellZscope+ device (nanoAnalytics) in 1:5 diluted media (1:5 BBB Identity Maintenance media : basal Vasculife without media supplements for iCE-BECs, or 1:5 Vasculife Maintenance media : basal Vasculife without media supplements for iECs). Diluted media was used to reduce the concentration of VEGF which is known to alter cell permeability. Impedance was continuously measured every 1 h, and TEER and capacitance (Ccl) were automatically calculated as readout parameters. TEER values for iCE-BECs at 18 hours were normalized to the iEC condition and data was presented as a fold change comparison.

Permeability assessment in microphysiological system

Barrier function of iCE-BECs and HBMVECs cultured in a microphysiological system were compared by assessing leakage of fluorescent dextran dye. iCE-BECs were generated according to the protocol described above and seeded in an OrganoPlate® 2-lane (9605-400-B, MIMETAS) according to the manufacturer's protocol [5]. Briefly, one day before the cell

seeding, ECM (3447-020-01, Cultrex 3D Collagen I, R&D Systems in 1M Hepes, Gibco and 37 mg/mL $NaHCO_3$) was prepared. Per tube, 35.000 cells were seeded and supplied with complete BBB identity maintenance media. HBMVEC cultures (OrganoReady® BBB HBMEC, MI-OR-HB-01, MIMETAS) were cultured according to the manufacturer's instructions. iCE-BECs and HBMVECs formed tubular structures against the ECM gel upon medium perfusion by placing the OrganoPlates on the OrganoFlow® perfusion rocker (MI-OFPR-S, MIMETAS). After 72 hours, media was replaced with 1:5 diluted media (1:5 BBB Identity Maintenance media : basal Vasculife without media supplements) for iCE-BECs. At day 4 of culture, 65-85 kDa TRITC dextran (0.25 mg/mL, Sigma-Aldrich, T1162) was added to the lumen of iCE-BECs and HBMVEC cultures. Images were taken every 2 minutes for a duration of 12 minutes, and at t=40 min after dye addition using an ImageXpress XLS Micro HCI System (Molecular Devices).

To test the barrier function of iCE-BECs in response to VEGF and across different lines (SFC086_03_03, BIONi010-C13, Alstem iPS26, and BIONi037-A) in a microphysiological system, cells were seeded in an OrganoPlate® 2-lane (9605-400-B, MIMETAS) as described above. After 72 h, media was replaced with 1:5 diluted media (1:5 BBB Identity Maintenance media : basal Vasculife without media supplements for iCE-BECs) for basal conditions or additionally supplemented with 0.2 µg/mL VEGF165 (293-VE-010, R&D). After 24 h incubation, differently sized FITC-dextrans (3.3 kDa, D3305; 40 kDa, D1845; 70 kDa D1822, all purchased from ThermoFisher) were applied at 10 µg/mL and immediately imaged using Opera Phenix High Content Imaging System (PerkinElmer) at 5× magnification, every five minutes for two hours. The ratio between mean intensity of FITC-dextran in the cell channel and the gel channel was calculated for each perfusable tube at each time point.

To calculate the apparent permeability, the slope of the linear regression was multiplied by the volume of the gel (0.0004136 cm^3) and subsequently divided by the surface area (0.01218153 cm^2) adapted from [6] according to the manufacture's protocol.

Brainshuttle™ transcytosis assessment in microphysiological system

To assess transcytosis of Brainshuttle™ molecules across iCE-BECs, cells were seeded in an OrganoPlate® 2-lane (9605-400-B, MIMETAS) as described above. After 72 h, media was replaced with 1:5 diluted media (1:5 BBB Identity Maintenance media : basal Vasculife without media supplements for iCE-BECs). After 24 h, fluorescently labeled Brainshuttle™ antibody or a non-targeting IgG control were applied at 200 nM, and immediately imaged using Opera Phenix High Content Imaging System (PerkinElmer) at 5× magnification, every 15 min for twelve hours. The ratio between mean intensity in the donor cell channel and the gel channel was calculated for each perfusable tube at each time point for both Brainshuttle™ and non-targeting IgG. To assess total transcytosis of Brainshuttle™, the slope of the linear regression was calculated within the time interval 0 to 12 h. Before each experiment, channels with matrix overflow or incomplete filling of matrix channel were excluded from the analysis.

Electron microscopy

To assess the ultrastructural morphology of endosomes, cell pellets of iCE-BECs were generated and fixed in 2.5% Glutaraldehyde (pH 7.4) overnight. After lipid fixation with 1% Osmium-Tetroxid for 1 h, samples were dehydrated with ascending ethanols and finally infiltrated with resin by incubating two times with Propylenoxid for 15 min each and Propylenoxid/epon (1:1 ratio) overnight at RT. Samples were transferred to epon blocks and polymerized at 60°C for 60 hours. Ultrathin sections (98 nm) were prepared on 200 mesh copper grids (EMS, Fort Washington, PA, USA) and afterwards contrasted with lead citrate and uranyl acetate. The sections were examined with a Philips CM10 transmission electron microscope equipped with a charge-coupled-device camera (Ultrascan 1000; Gatan) at an acceleration voltage of 80 kV.

Quantitative PCR

mRNA was extracted from iCE-BECs cell cultures with APOE genetic variants using Total RNA Miniprep Kit (T2010, Monarch) following the manufacturer's instructions. Complementary DNA was synthesized using iScript cDNA Synthesis kit (1708890, BioRad). Quantitative real-time PCR analysis was performed with Lightcycler 480 SYBR Green I Master mix (04887352001, Roche; LightCycler® 96 System, Roche). Ready-to-use primers from Origene for ApoE (HP200028), TFR1 (HP206788), FTH (HP205786), FTL (HP200131), FPN (HP210988), DMT-1 (HP200584), and GAPDH (HP205798) were used. Primer efficiency was determined by titration of cDNA from iCE-BECs ApoE3; all tested primers had an efficiency between 80%-110%. The cycle threshold (Ct) values were used for all experiments and were first normalized to endogenous control (GAPDH) levels by calculating the ΔCt for each sample. Values were then analyzed relative to control, to generate a ΔΔCt value. Fold change was obtained using the equation, expression fold change=$2^{-\Delta\Delta Ct}$.

Immunoblot

Cells were lysed with RIPA buffer (89900, LifeTech) and incubated for 30 min on a rotary shaker at 4°C. Cells were then centrifuged at 12,000 ×g at 4°C for 12 minutes, and the supernatant were retained for protein quantification using the Pierce bicinchoninic acid assay method (23225, ThermoFisher). 5-20 µg of protein was typically loaded per sample in NuPAGE LDS Sample Buffer (4x) (NP0007, Thermo Fisher) containing NuPAGE Sample Reducing Agent (NP0004, Thermo Fisher) as per manufacturer's instructions. Samples were denatured at 95°C for 5 min. Immunoblots were resolved using 4-15% Mini-Protean TGX Stain-Free gels (4568085, Bio-Rad) and transferred on 0.2 µm nitrocellulose membranes (1704159, Bio-Rad) using a Trans-Blot Turbo Transfer System, (BioRad). Membranes were subsequently blocked with 5% Milk in Tris-buffered saline with 0.1% Tween® 20 Detergent (TBS-T) for 1h followed by primary antibody incubation in 5% Bovine Serum Albumin (BSA) in TBS-T overnight (TfR1, 13-6800, ThermoFisher, 1:250; ApoE, Ab947, abcam, 1:500; FTH, 3998S, Cell Signaling Technology, 1:250; DMT-1, 20507-1-AP, ThermoFisher, 1:500; GAPDH-HRP, 8884, 1:25000, Cell Signaling Technology). After three times washing in TBS-

T, appropriate HRP-conjugated secondary antibodies in 5% BSA in TBS-T were applied (donkey-anti-rabbit-HRP, A16035, ThermoFisher, 1:2000; donkey-anti-goat-HRP, A16005, ThermoFisher, 1:2000; donkey-anti-mouse-HRP, A32788, ThermoFisher, 1:2000) for 1h at RT. Immunoblots were washed three times in TBS-T for five minutes each, and protein bands were then visualised using SuperSignal West Pico Plus Chemiluminescent Substrate (34580, ThermoFisher). Image acquisition was performed using the ChemiDoc MP (BioRad). Densitometric quantification of immunoblot bands was performed by measuring the optical density of the signal for each protein of interest and normalizing it to the optical density of the corresponding GAPDH band, followed by further normalization to the ApoE3 signal (ImageJ 1.54).

Whole cell proteomics

To benchmark iCE-BECs, primary brain endothelial cells (HBMVEC, n = 6 from three different batches), immortalized endothelial cells (HCMEC/D3, n = 3 different passages), and induced ECM-supported brain endothelial cells (iCE-BECs, n = 4 differentiations) were analyzed. HCMEC/D3 (SCC066, Merck) cells were cultured in EGM-2 Endothelial Cell Growth Medium-2 BulletKit (CC-3162, Lonza) for three sequential passages and collected when at confluence. Three independent batches of HBMVECs were purchased from AngioProteomie (cAP-0002, AngioProteomie) and cultured in EGM-2 Endothelial Cell Growth Medium-2 BulletKit (CC-3162, Lonza) in flasks coated with quick coating solution (cAP-01, Angioproteomie). Each cell batch was cultured for two subsequent passages and collected when at confluence. iCE-BECs were differentiated as previously described and collected on day 14 in culture. For whole proteomics analysis, culture media were removed and cells were washed with phosphate buffered saline (PBS). Cells were detached using trypsin 0.25%-EDTA (ThermoFisher, 25200056) for HCMEC/D3 or TrypLE^{TM} (12563011, ThermoFisher) for HBMVECs and iCE-BECs, resuspended in culture medium and centrifuged at 180 g at 4°C for 5 min. Cell pellets were then washed with cold PBS and centrifuged. This washing step

was repeated again once before snap freezing the cell pellets on dry ice for about 15 min. Cell pellets were then stored at -80°C.

Samples were reduced, alkylated, digested with trypsin and peptides purified using the PreOmics iST kit according to the supplier's specifications. Peptide samples were resuspended in 2% (v/v) acetonitrile and 0.5% (v/v) formic acid solution and 1 ug of peptides were analyzed by liquid chromatography (nano capillary system, EASY-nLC™ 1200 system, Thermo Scientific) on a C18 reverse-phase nano-high-performance liquid chromatography column connected to a mass spectrometer (Orbitrap Exploris™ 480, Thermo Scientific) via electrospray ionization. The DIA method consisted of one full range MS1 from 340 to 1210 m/z at 120k resolution, with a custom AGC target and 20ms max injection time. Then 28 DIA segments were acquired at 15k resolution with a standard AGC target and 20 ms max injection time. HCD fragmentation was set to normalized collision energy optimized for each segment. The spectra were recorded in profile mode. The default charge state for the MS2 was set to 3. Raw files have been processed with Spectronaut 18, with experiment settings based on BGS Default SNE for a DIA library free search, using global imputation to deal with missing values. Default settings included peptide and protein level false discovery rate control at 1 %. Measurements were normalized separately using local regression normalization. The mass spectrometric data were analyzed using Pulsar search engine as implemented in Spectronaut software, the false discovery rate on peptide and protein level was set to 1 %. A human UniProt fasta database (Homo Sapiens, 2022 07 01) was used for the search engine, allowing for 2 missed cleavages and variable modifications (N term acetylation and methionine oxidation).

Distributions of both raw and normalized data at the protein level were assessed to evaluate sample consistency. Principal Component Analysis (PCA) was conducted to reveal the overall data structure and to identify potential experimental artifacts. Outlier detection was implemented using the Mahalanobis distance, calculated from the first three principal components. No outliers were removed. The differential abundance analysis was performed to identify proteins with significant changes in expression levels between the different

conditions. For this statistical analysis, we utilized the R package “limma”. Unlike standard t-tests, which compare proteins individually, the “limma”; approach accounts for the overall variance observed across all proteins. This typically results in adjusted P-values, especially at the tails of the distribution, and is particularly well-suited for studies with small sample sizes.

Proliferation assay

To assess the proliferation rate between iPSC with ApoE3 and ApoE4 genetic variant, iPSCs were seeded on geltrex (A1413301, ThermoFisher) coated 96-well plate (PBK96G-1.5-F, MatTek USA, 12.000 cells/well) in ROCK inhibitor Y-27632 containing (SCM075, EMD Millipore) mTeSR Plus media (100-0276, Stemcell). Media was changed every 24 hours. Confluency of iPSCs was assessed every 24h for 8 consecutive days using Live/dead staining cell imaging kit (R37601, ThermoFisher) according to manufacturer's protocol. Live imaging of whole wells was performed with Opera Phenix High Content Imaging System (PerkinElmer) at 20× with three wells per condition and time point. Live cell area was measured by absolute threshold and expressed as percentage of total well area (confluency). Non-linear regression (logistic growth) was performed (GraphPad Prism 10.2.2.).

Flow cytometry

iCE-BECs with ApoE3 or ApoE4 genetic variant (80.000/well in 24-well plate) were treated with fluorescently labeled transferrin (T13342, ThermoFisher) at 25 µg/mL for 20 min at 37°C or left untreated, followed by incubation of CD31-AF700 (NB600-562AF700, Novus Biological, 1:100) for 30 min at 4°C. For testing marker expression in cells generated with the iEC or iCE-BEC protocol (100.000/well in a 24-well plate), cells were collected and incubated with primary antibodies in FACS buffer (PBS + 0.5% BSA + 2 mM EDTA) for 30 minutes at 4°C (see Table 3), The LIVE/DEAD™ Fixable Aqua Dead Cell Stain Kit (L34957, ThermoFisher) was used to determine cell viability for FACS analysis. For intracellular staining, cells were

stained with cell viability dye, fixed in 4% PFA for 30 min at RT, and then incubated with anti-vWF or anti-ERG in FACS buffer for 30 min at 4°C (see Table 3). Cells were subsequently washed with FACS buffer, and fluorescence was immediately acquired using a Flow Cytometer (CytoFLEX LX, CytExpert software, Beckman Coulter) in duplicates. Unstained negative controls were used to adjust gain for FITC, PE, or AF700, respectively. Per sample, at least 10,000 live cells (singlets) were acquired. Data was exported as in fcs format and analyzed in FlowJo v10.0.0 (BD Biosciences). After initial cleanup to remove debris, doublets and to select for live cells, we employed a two-step gating strategy for PECAM1-positive cells. The first gate identified PECAM1-positive cells using unstained controls as a baseline. Subsequently, within that positive population, a second more stringent gate was set at a considerably higher fluorescence level of PECAM1 in a way that isolate the iCE-BEC population, aiming for close to 100% positivity within this homogeneous group. Both gates, termed low and high PECAM1-expressing cells were consistently applied to all iEC and iCE-BEC samples. Frequencies of total PECAM1-positive cells of live cells and high PECAM1-positive expression cells of PECAM1-positive cells were extracted. For comparison of marker expressions in cells generated with the iEC or iCE-BEC protocol, cells were gated based on PECAM1-positive signal. Median fluorescence intensities (MFI) of VE-Cadherin, vWF, ERG, Claudin-5, GLUT1 and LDL-R were extracted in PECAM1-positive population and divided by the MFI of the unstained control for each condition and differentiation.

Live imaging of FerroOrange to assess labile iron pool

Intracellular labile iron was measured using BioTracker^{TM} FerroOrange Live Cell Dye, a fluorescent probe that specifically detects labile iron (II) ions (Fe^{2+}) only. Briefly, cells were washed and incubated with 1 µM BioTracker^{TM} FerroOrange Live Cell Dye (SCT210, Sigma) and cell marker (CytoTrace Green, 22017, AAT Bioquest) in HBSS at 37°C for 30min. Live cell imaging was performed using Opera Phenix High Content Imaging System (PerkinElmer) with a 63×/1.2 NA objective, 4 wells per condition, 10 fields per well, 8 z-stacks with 1 µm step size. Sum intensity of FerroOrange was normalized to the total area of the image

covered by cells (calculated using an absolute intensity threshold of CytoTrace Green). As controls, to artificially increase LIP, cells were treated with an iron donor, ferrous ammonium sulfate at 100 µM (FAS, 203505, Sigma), while treatment with iron chelator PIH at 10 µM (ab145871) was used to deplete LIP before FerroOrange Live cell dye was applied.

Calcein-AM assay to assess labile iron pool

Intracellular labile iron was measured using the metal-sensitive probe Calcein acetoxymethyl ester (Calcein-AM), as previously described [7]. This is a non-fluorescent dye that becomes fluorescent after enzymatic modification once it permeates the cell membrane [7-9]. This fluorophore binds iron stoichiometrically, which quenches its green fluorescence. In short, cells were washed, incubated with 0.5 µM of Calcein-AM (C1430, ThermoFisher) in HBSS and whole cell marker (HCS CellMask™ Stain Deep Red, H32721, ThermoFisher) for 20 min at 37°C. Iron chelator PIH at 10 µM (ab145871, abcam) was applied to half of the wells for 10 min. Cellular calcein fluorescence was measured in live cells using Opera Phenix High Content Imaging System (PerkinElmer) at 20×, 5 wells per condition, 16 fields per well, 3 z-stacks à 1 µm. The ratio between the sum intensity of calcein within the cell area (absolute threshold of CellMask) in untreated cells and iron chelator-treated cells was calculated, reflecting the amount of the labile iron pool. Fold changes were calculated by normalization to ApoE3 per each experiment.

Supplementary Table 3. Primary antibodies used for immunostainings

**Target**	**Concentration**	**Catalog No.**	**Vendor**
Early Endosome Antigen 1 (EEA1)	1:200	3288S	Cell signaling
Transferrin Receptor 1 (TfR1)	1:200	13-6800	ThermoFisher
VE-Cadherin	1:200	2500S	Cell signaling
VE-Cadherin (microphysiological system)	1:1000	ab33168	abcam

PECAM1	1:200	NB600-562	Novus Biologicals
PECAM1 (microphysiological system)	1:20	M0823	Dako
Ferritin Heavy chain (FTH)	1:200	ab65080	Abcam
Zonula occludens-1 (ZO-1)	1:200	33-9100	ThermoFisher
Divalent metal transporter 1 (DMT-1)	1:200	ab55735	Abcam
Claudin-5 (required methanol fixation)	1:200	352588	ThermoFisher
SLC2A1 (GLUT-1)	1:100	07-1401-AF1488	SigmaAldrich
ABCB1 (PgP)	1:50	919403	BioLegend
ABCC1 (MRP1)	1:100	PA5-88082	ThermoFisher
ABCA1 (CERP)	1:100	AF7207	R&D Systems

Supplementary Table 4. Secondary antibodies and fluorescent probes for immunostainings

**Class**	**Concentration**	**Origin specie**	**Catalog No**	**Vendor**
anti-rabbit-Alexa488	1:200	Donkey	711-545-152	Jackson ImmunoResearch
anti-mouse-Alexa488	1:200	Donkey	715-545-150	Jackson ImmunoResearch
anti-rabbit-Alexa647	1:200	Donkey	711-605-152	Jackson ImmunoResearch
anti-mouse-Alexa647	1:200	Donkey	715-605-150	Jackson ImmunoResearch
anti-sheep-Alexa647	1:200	Donkey	713-605-147	Jackson ImmunoResearch
Phalloidin-647	1:400	n.a.	65906	Sigma

Supplementary Table 5. Primary antibodies used for flow cytometry

Target	Conjugate	Concentration	Catalog No.	Vendor
Von Willebrand Factor	AF488	1:100	ab195028	abcam
ERG	AF488	1:50	ab196374	abcam
GLUT1	AF488	1:50	ab195359	abcam
PECAM1	AF700	1:100	NB600-562AF700	Novus Biological
PECAM1	PE	1:100	NB600-562PE	Novus Biological
Claudin-5	AF488	1:100	352588	ThermoFisher
VE-Cadherin	PE	1:50	AF938	R&D Systems
LDL Receptor	PE	1:50	LS-C130473	LSBio

Transferrin kinetics (uptake and pulse-chase)

To assess the uptake amount and rate of transferrin (pulse assay) and recycling rate of transferrin (pulse-chase assay), 25.000 cells were seeded in a 96-well glass bottom imaging plate (PBK96G-1.5-F, MatTek USA) in BBB Identity Maintenance media on day 11. On day 14, cells were washed once with PBS and incubated with the assay medium (EGM, cAP-02, Angioproteomie) containing 1% Bovine serum albumin for at least 10 min. Labeled transferrin (T13342, ThermoFisher) at 25 µg/mL was then applied between 2 and 45 min to assess the time course of transferrin uptake. To assess the recycling rate, cells were treated with labeled transferrin (T13342, ThermoFisher) at 25 µg/mL for 20 min, followed by application of 10-fold higher concentration of unlabeled Holo-transferrin (T0665, 250 µg/mL) for different time points (0 min - 60 min). Cells were fixed in 4% PFA for 20 min and washed three times with PBS

before counterstaining with Phalloidin-Atto-647 (65906, Sigma) and DAPI (D9542, Sigma). Images were acquired with Opera Phenix High Content Imaging System (PerkinElmer) using the 40×/1.1 NA water long WD confocal objective, Binning 2x2, Camera ROI 2160x2160, 20 planes à 1 µm. Per time point, minimum 50 images were acquired.

Live imaging of endosomal pH

To estimate endosomal pH, 15.000 cells were seeded in each well of a 96-well glass bottom imaging plate (PBK96G-1.5-F, MatTek USA) in BBB Identity Maintenance media 72 h before the experiment. After washing with PBS, cells were incubated with 25 µg/ml of pHrodo™ Red Transferrin Conjugate (P35376, ThermoFisher) and 25 µg/ml of AF647-conjugated Transferrin (T23366, ThermoFisher) for 10 minutes at 37°C 5% CO_2. Cells were washed twice with BBB Identity Maintenance media, and were subsequently imaged with a DMI8 fluorescence microscope (Leica Microsystems) equipped with a stage incubator controlling at 37 ºC and 5% CO2. Full frame 1024 x 1024 images were acquired with HCX PL APO 100X/1.4 NA oil objectives with a resolution of 0.288 µm, three z-stacks with 0.75 µm step size. Using maximum projection images, vesicles containing both AlexaFluor647-conjugated transferrin and pHrodo™ Red Transferrin were detected (colocalization threshold 0.35). Per endosome, the ratio of integrated vesicular intensity of pHrodo™ Red Transferrin and AlexaFluor647- conjugated transferrin was calculated (MotionTracking 8.97, http://motiontracking.mpi-cbg.de/get/) as previously described [76].

Live imaging of sorting tubule biogenesis

To assess the formation of sorting tubules, 15.000 cells were seeded in a 96-well glass bottom imaging plate (PBK96G-1.5-F, MatTek USA) in BBB Identity Maintenance media 48 h before the experiment. After washing with PBS, cells were incubated with 25 µg/ml of fluorescently labeled transferrin (T13342, ThermoFisher) for at least three hours at 37°C 5% CO_2. After washing with BBB Identity maintenance media, cells were imaged with a DMI-8 TIRF microscope (Leica Microsystems) equipped with a stage incubator controlling at 37 ºC and 5% CO_2.

Full frame 1024 x 1024 images were acquired with HCX PL APO 100X/1.4 NA oil objectives with a resolution of 0.288 µm, two z-stacks with 1 µm step size. Single color images were acquired with a 488 nm TIRF laser for a final rate of 2 frames per second for 1 min each. The number of tubules per cell occurring within 1 min acquisition was quantified manually in the maximum intensity projection of the z-sections for each movie by two independent blinded raters.

Immunostainings

After fixation of the cells with 4% PFA (15710, Electron Microscopy Science), cells were quickly washed with PBS for three times, before permeabilized with 4% gelatin (G7041, Sigma) + 0.1% Saponin (84510, Sigma) in PBS for 10 min at RT. Primary antibodies were incubated overnight at 4 °C (see Table 1). After washing three times with PBS, fluorescently labeled secondary antibodies and Phalloidin-Atto-647 (65906, Sigma) were applied for one hour at room temperature (RT, see Table 2). Nuclei were counterstained with DAPI (Sigma, D9542) for 10 min at RT. For representative images of PECAM1, VE-Cadherin, Claudin-5, SLC2A1 (GLUT-1), ABCB1 (PgP), ABCC1 (MRP1), ABCA1 (CERP), confocal images at SP-8 using 63×/1.2 NA objective or a DMI-8 TIRF microscope at 20×/0.55 NA magnification (Leica Microsystems) were acquired. For quantification of iron-related proteins (FTH and DMT-1), Opera Phenix High Content Imaging System (PerkinElmer) was used at 40× magnification. Bright field images were acquired with an IX83 microscope (Olympus) with a 10×/0.3 NA objective.

Immunostainings (microphysiological system)

The OrganoPlate cultures were fixed with 3.7% formaldehyde (252,549, Sigma) or 100% methanol (494,437, Sigma). Immunostaining was performed as previously reported [5,10]. Briefly, cultures were permeabilized using Triton X-100 (T8787, Sigma) for 10 min followed by a blocking step with a buffer containing FBS (A5670801, Thermo Fisher), bovine serum albumin (BSA, 5217, R&D Systems) and Tween-20 (P9416, Sigma) for 45 min. Primary antibodies (see Table 1) were incubated in the blocking buffer overnight at 4°C after which

secondary antibodies (see Table 2) were incubated for 1 h at RT. Nuclei were stained with Hoechst (H3570, ThermoFisher). Representative images were acquired at 10×/0.45 magnification using ImageXpress Micro XLS and Micro XLS-C HCI Systems (Molecular Devices).

High content imaging and image analysis for transferrin kinetics and immunostainings

The Perkin Elmer's Harmony high-content analysis software 5.1 (HH17000001) was used to set up the plate dimensions enabling the fast and efficient imaging with the Opera Phenix High Content Imaging System (PerkinElmer). For transferrin kinetics and immunostainings for iron-related protein experiments, we used the 40× long WD confocal objective. Per well, 50 images were acquired, 20 planes per image with a section thickness of 1 µm per plane. Appropriate channels were selected (DAPI, Alexa-488, Alexa-647) and exposure time as well as focus height was set accordingly and kept the same between wells. Perkin Elmer's Harmony high-content analysis software 5.1 was used to analyze the images. In brief, basic flat field correction and maximum projection was applied. Cell area was determined by a binary threshold mask using the Phalloidin signal. Sum intensity of the respective markers (transferrin-488, FTH, FPN, DMT-1) was calculated within the cell area and normalized to its area. Linear background reduction was applied. For assessment of the transferrin uptake rate, intensity values were normalized to the 10 min time point, while for transferrin recycling rate, intensity values were normalized to the time point of 20 min pulse, no chase.

Confocal microscopy and image analysis of TfR1 and EEA1

For quantification of TfR1 or EEA1 in iCE-BECs, ten images per condition were acquired using the confocal microscope with a 63×/1.2 NA objective. Pixel size was adjusted to 100 nm at 1024x1024 frame size. Per image, five z-stacks with a step size of 0.5 µm were used. Pinhole was adjusted to an optical thickness of 1 um and sequential acquisition between stacks was selected. Laser power was adjusted according to the marker expression and not changed between experimental groups. Vesicles containing EEA1 or TfR1 were detected

and mean integrated vesicular intensity within cells (absolute threshold for Phalloidin) was calculated, normalized to the cell area (MotionTracking 8.97, http://motiontracking.mpi-cbg.de/get/) as previously described[11].

Supplementary Video S1 - related to Figure 5.

Representative video of transferrin (green) intracellular transport in live iCE-BECs (isogenic pair 1) with ApoE3 gene variant. Cells were incubated with fluorescently labeled transferrin for three hours and then videos of one minute were acquired at 100× using a Widefield microscope. Representative image frames of those videos are shown in Figure 5G.

Supplementary Video S2 - related to Figure 5.

Representative video of transferrin (green) intracellular transport in live iCE-BECs (isogenic pair 1) with ApoE4 gene variant. Cells were incubated with fluorescently labeled transferrin for three hours and then videos of one minute were acquired at 100× using a Widefield microscope. Representative image frames of those videos are shown in Figure 5G.

1. Lu TM, Houghton S, Magdeldin T, et al. Pluripotent stem cell-derived epithelium misidentified as brain microvascular endothelium requires ETS factors to acquire vascular fate. *Proceedings of the National Academy of Sciences.* 2021;118(8):e2016950118.
2. Ewels P, Magnusson M, Lundin S, Käller M. MultiQC: summarize analysis results for multiple tools and samples in a single report. *Bioinformatics.* 2016;32(19):3047-3048.
3. Liao Y, Smyth GK, Shi W. featureCounts: an efficient general purpose program for assigning sequence reads to genomic features. *Bioinformatics.* 2013;30(7):923-930.
4. Robinson MD, McCarthy DJ, Smyth GK. edgeR: a Bioconductor package for differential expression analysis of digital gene expression data. *Bioinformatics.* 2010;26(1):139-140.
5. Wevers NR, Kasi DG, Gray T, et al. A perfused human blood-brain barrier on-a-chip for high-throughput assessment of barrier function and antibody transport. *Fluids Barriers CNS.* 2018;15(1):23.
6. Ragelle H, Dernick K, Khemais S, et al. Human Retinal Microvasculature-on-a-Chip for Drug Discovery. *Advanced Healthcare Materials.* 2020;9(21):2001531.
7. Breuer W, Epsztejn S, Millgram P, Cabantchik IZ. Transport of iron and other transition metals into cells as revealed by a fluorescent probe. *Am J Physiol.* 1995;268(6 Pt 1):C1354-1361.
8. Tenopoulou M, Kurz T, Doulias P-T, Galaris D, Brunk UT. Does the calcein-AM method assay the total cellular 'labile iron pool' or only a fraction of it? *The Biochemical journal.* 2007;403(2):261-266.
9. Thomas F, Serratrice G, Béguin C, et al. Calcein as a Fluorescent Probe for Ferric Iron: APPLICATION TO IRON NUTRITION IN PLANT CELLS *. *J Biol Chem.* 1999;274(19):13375-13383.
10. Nair AL, Groenendijk L, Overdevest R, et al. Human BBB-on-a-chip reveals barrier disruption, endothelial inflammation, and T cell migration under neuroinflammatory conditions. *Front Mol Neurosci.* 2023;16:1250123.
11. Rink J, Ghigo E, Kalaidzidis Y, Zerial M. Rab conversion as a mechanism of progression from early to late endosomes. *Cell.* 2005;122(5):735-749.

Supplementary Figures

Supplementary Figure 1 - related to Figure 1

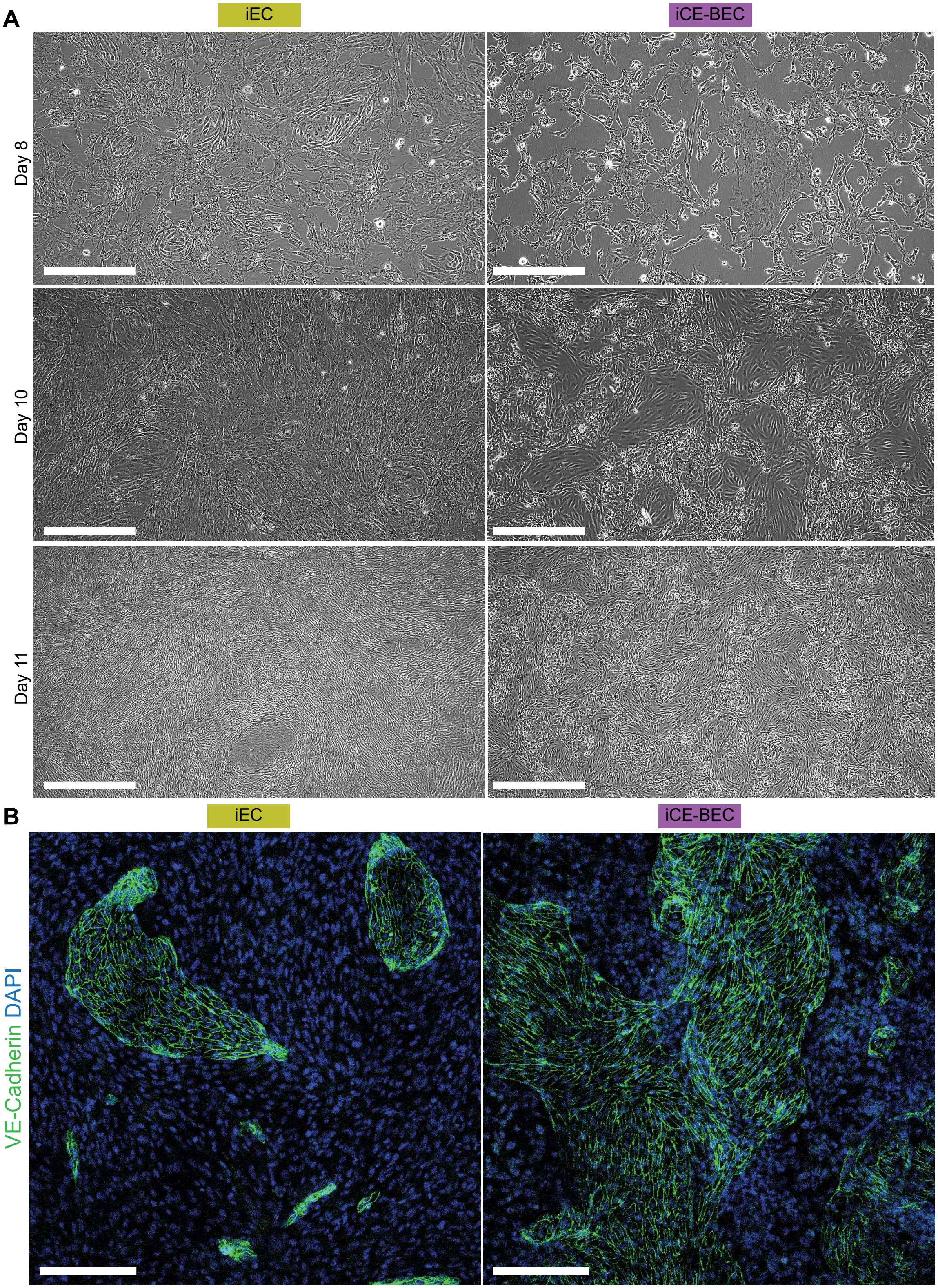

Supplementary Figure 1 - related to Figure 1. A, Representative brightfield images of iEC and iCE-BECs during the differentiation at day 8, day 10, and day 11. Scale bar, 500 µm. **B,** Representative fluorescence images of iEC and iCE-BECs after immunostaining for VE-Cadherin (green) at day 11 before MACS sorting. DAPI-stained nuclei are shown in blue. Scale bar, 250 µm.

Supplementary Figure 2 - related to Figure 2 and 3

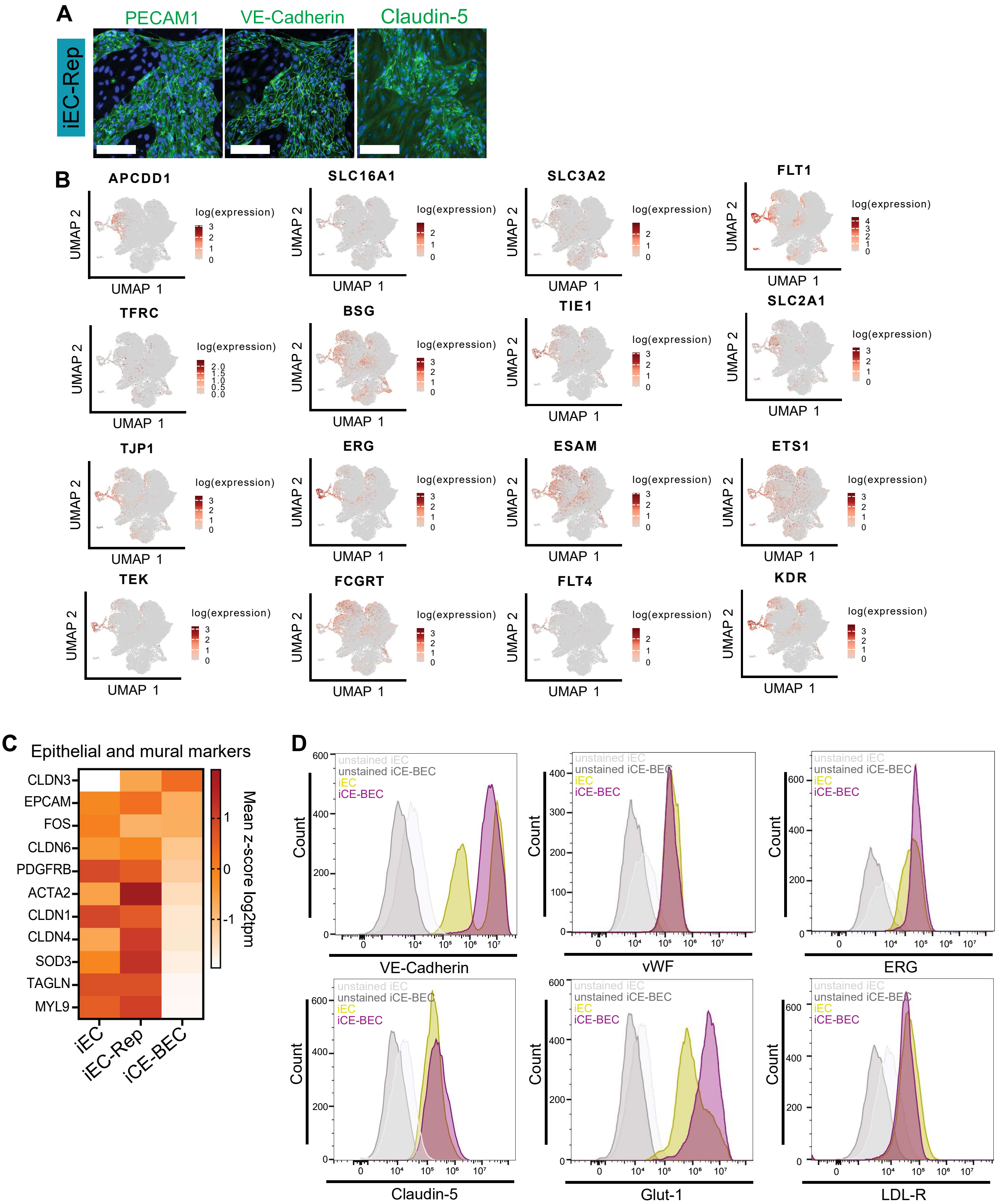

Supplementary Figure 2 - related to Figures 2 and 3.

A, Representative fluorescence images after immunostaining with endothelial-specific markers of iEC-rep at day 14. Cells are pseudo-colored showing PECAM1, VE-Cadherin or Claudin-5 in green, and DAPI-stained nuclei in blue. Scale bar, 200 µm. **B,** Feature plots showing normalized log expression of marker genes of endothelial and mural markers, plotted on the UMAP from Figure 2A. **C,** Bulk RNA-Seq heatmap showing expression of epithelial and mural markers across the three differentiation protocols, iEC, iEC-rep, and iCE-BECs. Values are expressed as mean z-score $\log_2$tpm, with three independent differentiations per condition. **D,** Representative flow cytometry histograms showing fluorescence intensities of endothelial cell marker (ERG, vWF), tight junction proteins (VE-Cadherin, Claudin-5), transporters (GLUT1) and receptors (LDL-R) in PECAM1-positive single (live), iEC (yellow), iCE-BEC (purple) and their respective unstained control (light gray for iEC, dark grey for iCE-BEC). Intensity values are shown on the x-axis, while the y-axis displays the number of cells.

Supplementary Figure 3 - related to Figure 4

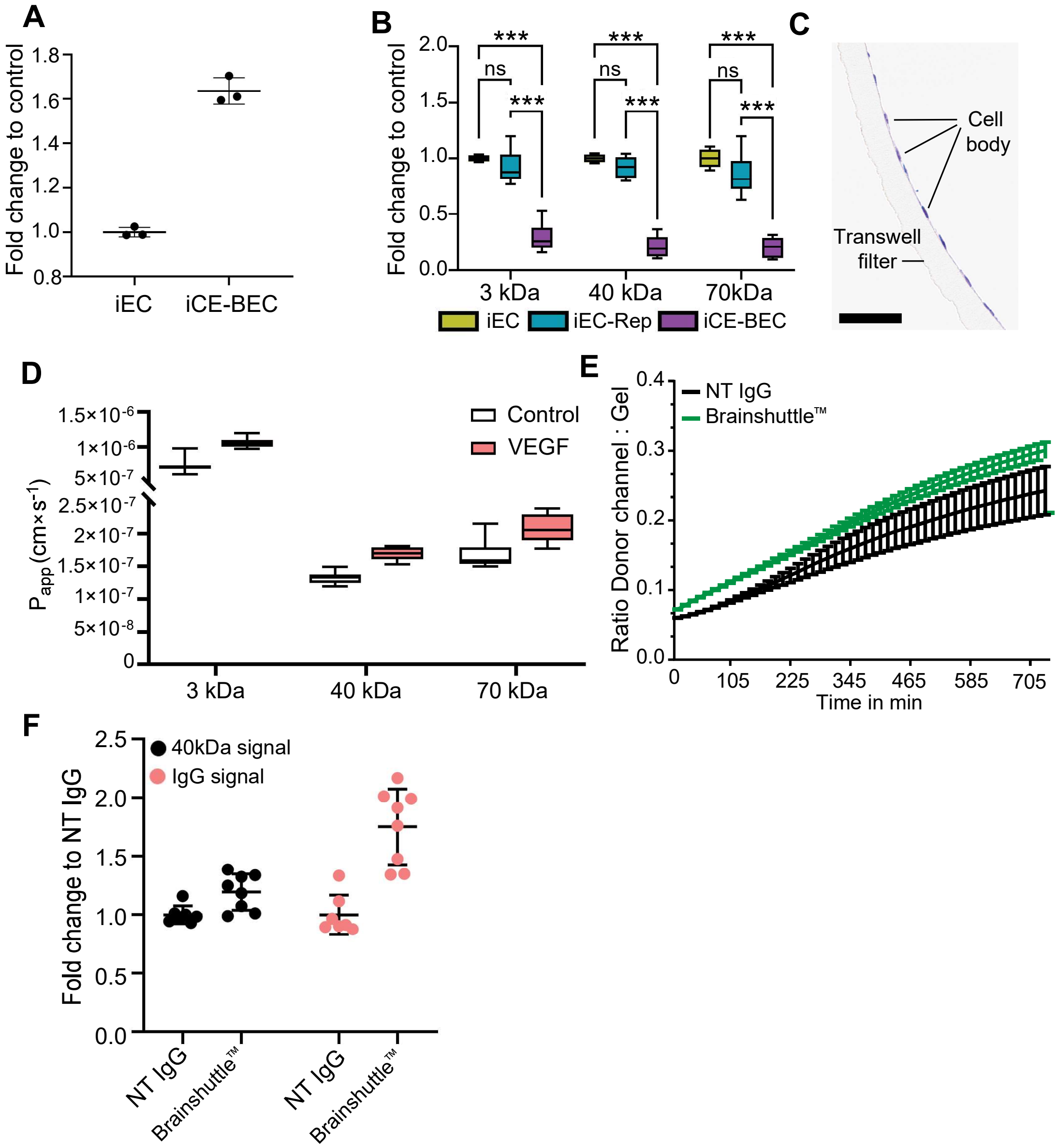

Supplementary Figure 3 - related to figure 4.
A, Normalized TEER values for iCE-BEC and iECs at 18 hours.

Data from 3 technical replicates per condition. TEER values were normalized to iECs for fold change comparison. **B,** Quantification of relative apparent permeability (P_{app}) for dextrans of different molecular weights of cells generated with the protocols described in Fig. 1A using a transwell system. Values were normalized to the apparent permeability of iECs. Graph shows boxplots with interquartile ranges and median. Lines show the 5th and 95th percentiles, data from n = 4 independent differentiations with 8 technical replicates per condition. Differences in apparent permeability are statistically significant as evaluated by Two-way ANOVA with Sidak multiple comparisons. ns, not statistically significant; ***, p <0.001. **C,** Haematoxylin and Eosin staining of iCE-BECs grown on a transwell filter showing a cell monolayer. Scale bar, 50 µm. **D,** Quantification of apparent permeability (Papp) of iCE-BECs to 3, 40 or 70 kDa dextran in basal conditions or after stimulation with 200 ng/mL VEGF-A for 24 hours. Graph shows boxplots with interquartile ranges and median. Lines show the 5th and 95th percentiles, data from one differentiation with at least 7 technical replicates per condition. **E,** Representative antibody transcytosis curves across iCE-BECs after incubation with 200 nM non-targeting IgG (NT IgG) or Brainshuttle™ antibody. Images were acquired immediately after incubation for 12 hours and ratio between donor channel and gel channel signals were plotted against time (see methods for details). Each curve shows mean ± SEM data from 8 chambers. **F,** Quantification of relative IgG transcytosis (IgG signal) and apparent permeability to 40 kDa dextran (40 kDa signal) across iCE-BECs after incubation with 200 nM non-targeting IgG (NT IgG) or a Brainshuttle™ antibody. Antibody and dextran values are measured in the same channels and are normalized relative to the NT IgG condition. Graph shows mean ± SD of one differentiation with 8 technical replicates per condition.

Supplementary Figure 4 - related to Figure 5

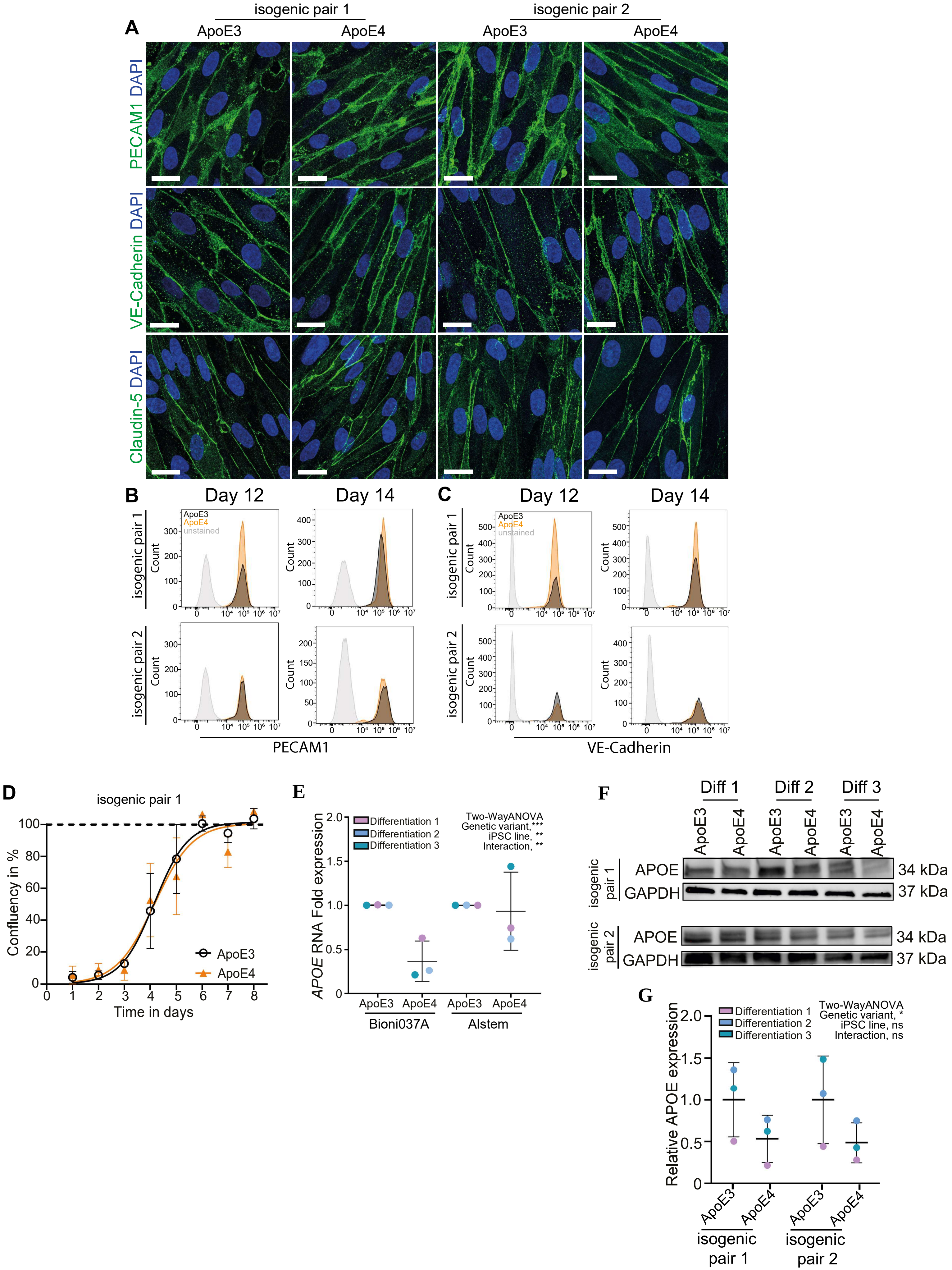

Supplementary Figure 4 - related to Figure 5.

A, Representative fluorescence images after immunostaining with endothelial-specific markers in iCE-BECs of two isogenic pairs (Bion037A, isogenic pair 1; Alstem, isogenic pair 2) with ApoE3 or ApoE4 genetic variant. Cells are pseudo-colored showing PECAM1, VE-Cadherin, ZO-1 or Claudin-5 in green, and DAPI-stained nuclei in blue. Scale bar, 20 µm. **B-C,** Representative flow cytometry histograms showing fluorescence intensities of PECAM1 (B), VE-Cadherin (C) in iCE-BECs with ApoE3 (black) or ApoE4 (orange) genetic variants or unstained control (grey). Intensity values are shown on the x-axis, while the y-axis displays the number of cells at day 12 and day 14.

D, Assessment of proliferation rate of iPSC line (isogenic pair 1) with ApoE3 and ApoE4 genetic variants over eight consecutive days. Live/dead staining and live imaging of whole wells was performed with Opera Phenix High Content Imaging System (PerkinElmer) at 20× magnification with three wells per condition and time point. Live cell area was measured by absolute threshold and expressed as percentage of total well area (confluency) ± SD. Non-linear regression (logistic growth) was performed. **E,** Quantification of relative *ApoE* mRNA expression by quantitative PCR. Graph shows mean ± SD of both isogenic pairs with each n = 3 independent differentiations with 3 technical replicates per experiment. Points represent independent differentiations of both isogenic pairs. Differences in *ApoE* mRNA expression were evaluated by a Two-Way ANOVA: significant main effect of genetic variant on *ApoE* expression, $p < 0.001$, significant main effect of iPS line on *ApoE* expression, $p < 0.01$, and significant interaction between iPS line and genetic variant, $p < 0.01$. **F,** Representative Western Blot image showing ApoE protein expression in iCE-BECs with ApoE3 or ApoE4 genetic variants. Data come both isogenic pairs from three independent differentiations (Diff). **G,** Quantification of relative APOE protein expression from Western Blot in (**F**). Graphs show mean ± SD. Points represent independent differentiations. *, $p < 0.05$, significant main effect of genetic variant on APOE expression, no significant effect of iPSC line on APOE expression and no significant interaction between iPSC line and genetic variant by Two-Way ANOVA with n = 3 independent differentiations.

Supplementary Figure 5 - related to Figure 6

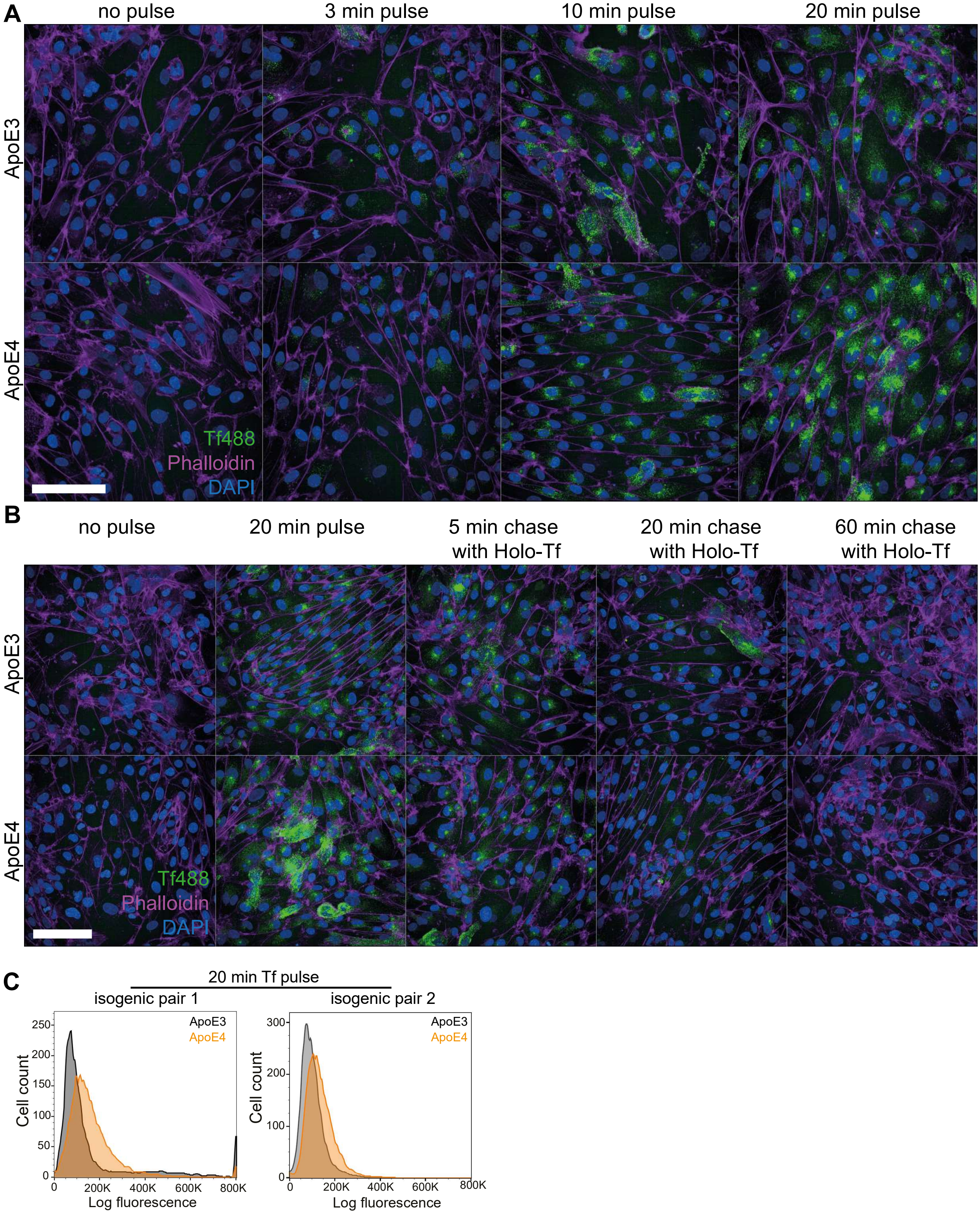

Supplementary Figure 5 - related to Figure 6.

A, Representative maximum projection confocal images of iCE-BECs with ApoE gene variants from pulse assay and **B,** pulse-chase assay assessing transferrin trafficking kinetics. Briefly, cells were incubated with fluorescently labeled transferrin and fixed after different time points for continuous uptake (pulse assay) while for recycling assessment (pulse-chase assays), cells were incubated for 20 min with fluorescently labeled transferrin followed by incubation of 10-fold higher concentration of unlabeled holo-Transferrin for different time points. Cells are pseudo-colored showing Transferrin in green, Phalloidin in magenta, and DAPI-stained nuclei in blue. Scale bar, 100 µm. For each time point, 50 images were acquired at 40× using a high content screening system, maximal projections were used to quantify sum intensity of transferrin in Phalloidin-positive area. **C,** Flow cytometry assessment of transferrin uptake after 20 min incubation in live iCE-BECs of both isogenic pairs with ApoE3 and ApoE4 genetic variants. Histograms show log fluorescence intensities of fluorescently labeled transferrin on the x-axis and the number of cells on the y-axis. ApoE3 iCE-BECs are shown in black, ApoE4 iCE-BECs in orange.

Supplementary Figure 6 - related to Figure 7

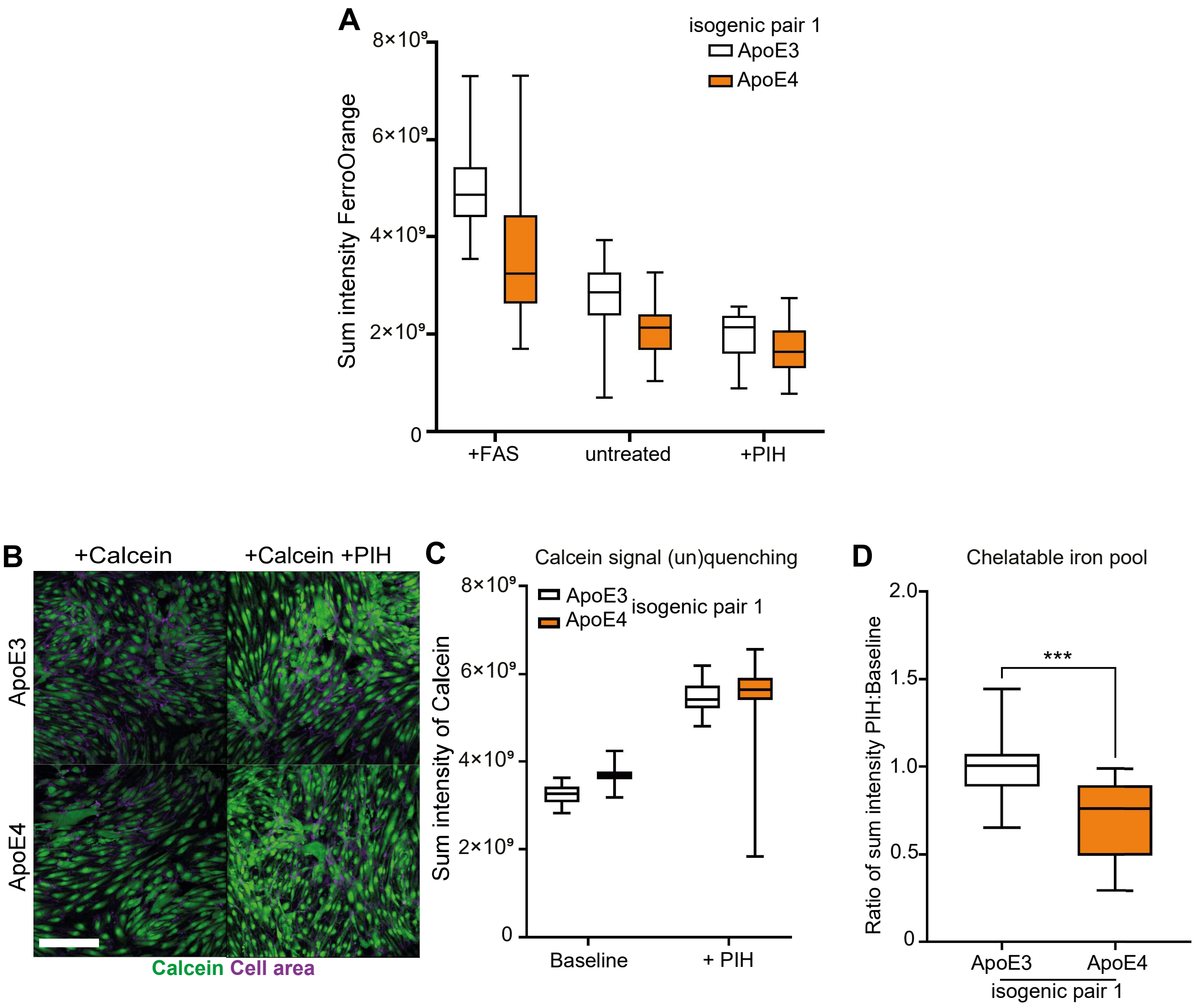

Supplementary Figure 6 - related to Figure 7.
A, Mean intensity of FerroOrange in iCE-BECs with ApoE3 or

ApoE4 gene variant (isogenic pair 1). As controls, cells have been treated with an iron donor ferrous ammonium sulfate (FAS) or iron chelator pyridoxal isonicotinoyl hydrazone (PIH) for 30 min before incubating with FerroOrange, a fluorescent probe that specifically detects labile iron (II) ions (Fe^{2+}) in live cells. Graph shows boxplots with interquartile ranges and median. Lines show the 5th and 95th percentiles, data from one differentiation, 40 images per condition have been acquired at 63× using a high content screening system, maximal projections were used to quantify sum intensity of FerroOrange within cells. **B,** Representative images of iCE-BECs with ApoE gene variants (isogenic pair 1) incubated with the metal-sensitive probe calcein acetoxymethyl ester (calcein-AM), which quenches its green fluorescence when binding to iron and unquenches upon iron chelator treatment with iron chelator pyridoxal isonicotinoyl hydrazone (PIH). Cells were treated with an iron chelator PIH or left untreated. Cellular calcein fluorescence was measured in live cells using high content screening system at 20×. Cells are pseudo-colored showing Calcein in green, plasma membrane in magenta. Scale bar, 200 µm.**C,** Sum intensity of calcein was normalized per cell area shown in a representative experiment illustrating the calcein signal (un)quenching upon iron chelator (+PIH) treatment. **D,** The ratio between the mean intensity of Calcein within the cell area in untreated cells (baseline) and iron chelator-treated cells (+PIH) was calculated, reflecting the amount of the labile iron pool. Graph shows boxplots with interquartile ranges and median. Lines show the 5th and 95th percentiles, data from isogenic pair 1 with n = 3 independent differentiations with 120 images per experiment. Differences in the FerroOrange intensity between ApoE genetic variants are statistically significant as evaluated by the Mann-Whitney-U test ($p < 0.001$).

Supplementary Figure 7 - related to Discussion

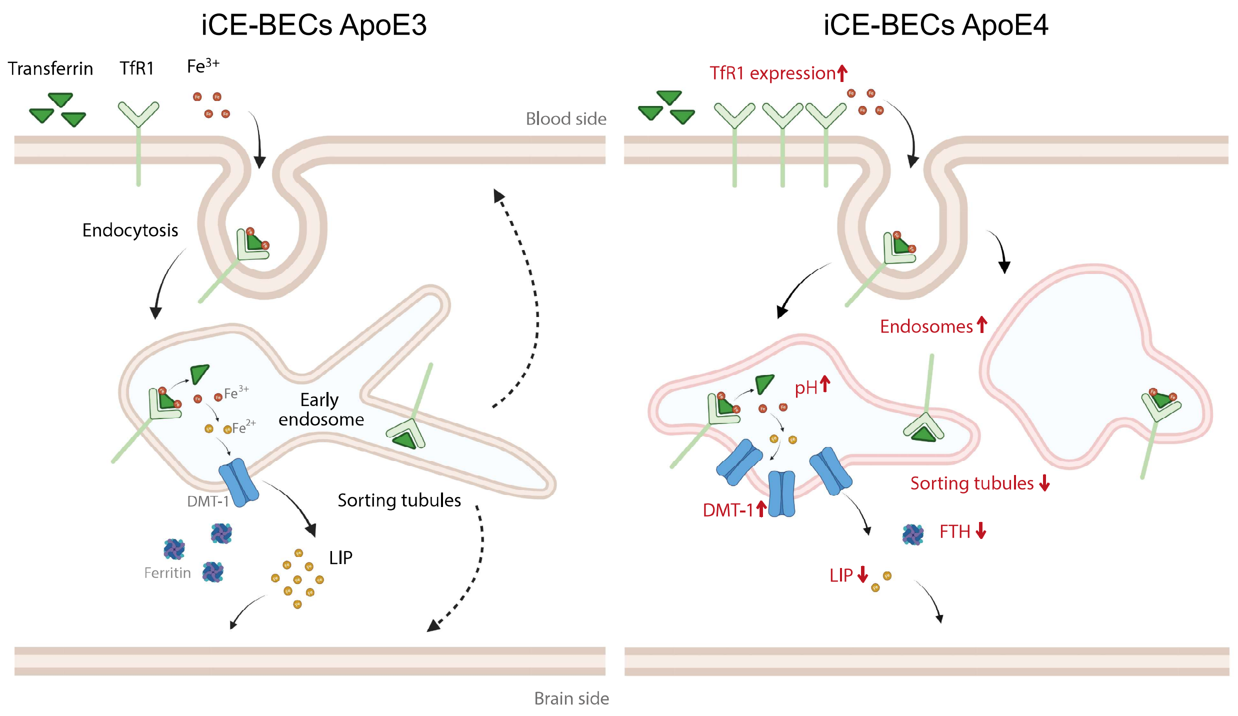

Supplementary Figure 7 - related to discussion.

Schematic summarizing intracellular transport of transferrin and iron in BECs with ApoE3 genetic variant and the key changes occurring in ApoE4 iCE-BECs: a) early endosome enlargement, increased pH and reduced sorting tubule biogenesis. b) Reduced intracellular labile iron pool (LIP), potentially caused by defects in endosomal maturation. c) Changes in expression of proteins regulated by iron-responsive elements, including Divalent metal transporter 1 (DMT-1), transferrin receptor TfR1 (both increased) and Ferritin (FTH, also reduced). d) Increased transferrin uptake driven by higher TfR1 expression. Figure created in biorender.com.
