# Supplementary material for: ApoE4 disrupts intracellular trafficking and iron homeostasis in a reproducible iPSC-based model of human brain endothelial cells
Source: Stem Cell Reports. 2025 Aug 21;20(9):102607. doi: 10.1016/j.stemcr.2025.102607 (PMC12447338; doi:10.1016/j.stemcr.2025.102607)
Supplement: Document S2. Article plus supplemental information [file mmc5.pdf]

Stem Cell Reports

Article

OPEN ACCESS

ApoE4 disrupts intracellular trafficking and iron homeostasis in a reproducible iPSC-based model of human brain endothelial cells

Luisa Bell,[1,2] Shane Clerkin,[1] Sila Rizalar,[1] Antoine Rizkallah,[1] Nadine Stokar-Regenscheit,[1] Xandor M. Spijkers,[3] Nienke R. Wevers,[3] Claire Simonneau,[1] Angélique Augustin,[1] Barbara Höllbacher,[1] Lia D'Abate,[1] Joanna Ficek-Pascual,[1] Kim Schneider,[1] Desiree Von Tell,[1] Thomas Maurissen,[1] Chiara Zanini,[1] Christelle Zundel,[1] Sabrina Golling,[1] Christine Becker,[1] Alex Odermatt,[2] Lynette C. Foo,[1] Martina Pigoni,[1,*] and Roberto Villaseñor[1,4,*]

[1]Roche Pharma Research and Early Development (pRED), Roche Innovation Center Basel, Basel, Switzerland

[2]Department of Pharmaceutical Sciences, University of Basel, Basel, Switzerland

[3]MIMETAS BV, Oegstgeest, the Netherlands

[4]Lead contact

*Correspondence: martina.pigoni@roche.com (M.P.), roberto.villasenor_solorio@roche.com (R.V.)

https://doi.org/10.1016/j.stemcr.2025.102607

SUMMARY

Transferrin receptor in brain endothelial cells can deliver therapeutic antibodies to the brain via transcytosis across the blood-brain barrier (BBB). Whether receptor transport remains intact in Alzheimer disease is still a major open question. Here, we investigated whether apolipoprotein E4 (ApoE4), the major genetic risk factor for Alzheimer disease, altered intracellular transport in human brain endothelial cells. To achieve this, we first developed a reproducible protocol for induced pluripotent stem cells based on a defined chemical cocktail and extracellular matrix support to differentiate brain endothelial cells (iCE-BECs). Multi-omics profiling and functional transport assays showed that iCE-BECs have a brain endothelial gene signature and recapitulate receptor-mediated transcytosis of a clinically validated Brainshuttle antibody against transferrin receptor. Engineered iCE-BECs homozygous for ApoE4 had impaired endosome maturation, increased transferrin receptor expression, and reduced cytoplasmic iron. Our data revealed that ApoE4 can impact intracellular transport and iron homeostasis at the BBB in a cell-autonomous manner.

INTRODUCTION

The blood-brain barrier (BBB) is formed by brain endothelial cells, pericytes, and astrocytes organized into a neurovascular unit that regulates the exchange of proteins between blood circulation and brain parenchyma via receptor-mediated transcytosis (Abbott 2013). The transferrin receptor (TfR1) is one of the best characterized receptors involved in transcytosis and is validated as a target to deliver therapeutic antibodies to the brain parenchyma (Bien-Ly et al., 2014; Grimm et al., 2023). Intracellular sorting in endosomes determines whether a receptor undergoes transcytosis or is instead transported to lysosomes for degradation (Villaseñor et al., 2019). Whether intracellular trafficking in brain endothelial cells is altered in disease conditions and to what extent this impacts transcytosis across the BBB remains largely unknown.

There is a wealth of evidence that documents the impact of disease conditions on BBB paracellular permeability. For example, it is well established that ApoE4, the major genetic risk factor for sporadic Alzheimer disease (AD), increases paracellular permeability of the BBB *in vivo* (Halliday et al., 2016; Yamazaki et al., 2020; Montagne et al., 2021). On the other hand, disease-specific changes to intracellular transport and/or transcytosis across the BBB are still relatively unexplored. A recent transcriptomic analysis of the vasculature of AD brain tissue showed substantial changes in gene expression levels associated with intracellular trafficking, including downregulation of TfR1 in brain capillaries (Yang et al., 2022). However, disease subgroups or risk factor genotypes could further affect TfR1 expression and trafficking. For instance, it is known that ApoE4 expression in mice led to upregulation of TfR1 expression in brain endothelial cells (Barisano et al., 2022). This highlights the need for a systematic approach to evaluate how specific risk factors affect transport across the BBB.

Human stem-cell-based models using brain endothelial cells are a powerful tool to investigate how disease-related conditions might affect BBB integrity (Blanchard et al., 2020). However, earlier studies used models with an overt epithelial signature that lacked expression of genes required for endothelial function (Lu et al., 2021). Since the mechanisms of intracellular transport in endothelial and epithelial cells are different (Villaseñor et al., 2019), induced pluripotent stem cells (iPSCs)-based models with epithelial identity are not suitable to investigate the regulation of transcytosis across the BBB. To address this, we developed a protocol to differentiate endothelial cells with brain-specific identity from iPSCs. We show that these cells express key markers of brain endothelial cells and recapitulate receptor-mediated transcytosis of a clinically validated TfR1-dependent Brainshuttle antibody. Using this differentiation protocol on genetically engineered iPSC

Published by Elsevier Inc. on behalf of International Society for Stem Cell Research.
This is an open access article under the CC BY license (http://creativecommons.org/licenses/by/4.0/).

lines, we found that ApoE4 regulates the maturation of early endosomes in a cell-autonomous manner in brain endothelial cells. Finally, we show that ApoE4 also dysregulates iron homeostasis, triggering upregulation of TfR1 in brain endothelial cells. Our data highlight the utility of relevant human *in vitro* models of brain endothelial cells to investigate how disease risk factors affect intracellular transport and reveal a new role for ApoE4 in the regulation of iron metabolism at the BBB.

RESULTS

Characterization and validation of a novel protocol for differentiation of brain endothelial cells from iPSC to investigate protein transport

To generate human brain endothelial cells from iPSC, we first triggered mesoderm induction in the cells, as previously described (Patsch et al., 2015). Mesoderm-committed cells were next differentiated toward an endothelial identity using VEGF165 and forskolin. We then treated these cells with a defined chemical cocktail that modulates key pathways associated with induction of BBB properties during development: Wnt, cAMP, and transforming growth factor beta (TGF-β) (Figure 1A; methods for details). In addition to this chemical cocktail, we replated the cells on vitronectin-coated plates, as this extracellular matrix (ECM) glycoprotein has a key role in both maintaining barrier properties and regulating transcytosis in mice (Ayloo et al., 2022). We termed the cells generated by this protocol iCE-BECs (inducible differentiation via chemical cocktail and extracellular matrix support for brain endothelial cells). We compared iCE-BECs to human iPSC-derived endothelial cells obtained via mesoderm induction, referred here as iECs. The iCE-BEC protocol resulted in a higher number of PECAM1-positive cells at day 11 before cell sorting by MACS (Figures 1B and S1). After sorting by PECAM1 and culturing for 3 days in their respective maintenance media, the percentage of PECAM1-positive cells was close to 100% for both protocols (Figure 1C). However, iECs showed two populations expressing different levels of PECAM1 with high heterogeneity across experiments (Figures 1C and 1D). In contrast, the iCE-BEC protocol resulted in a homogeneous population of PECAM1-positive cells reproducibly across experiments (Figures 1C and 1D). Similarly, the expression of VE-Cadherin and Claudin-5 was heterogeneous in iECs and more homogeneous in iCE-BECs (Figure 1D). These data suggest that the iCE-BEC protocol leads to an endothelial cell population that is more homogeneous and reproducible compared to iECs.

Previous work showed that inhibition of TGF-β signaling by RepSox enhanced barrier properties in iECs (Roudnicky et al., 2020). We therefore asked whether iCE-BECs improved barrier properties beyond those triggered by RepSox alone. To this end, we compared iCE-BECs and iECs with iECs treated with RepSox (iEC-Rep; Figures 1A and S2A) using single-cell RNA sequencing (RNA-seq) and bulk RNA-seq. Analysis was performed on day 14 in culture, and data were integrated to assess the composition of the cell populations obtained with each protocol (Figure 2A).

Single-cell RNA-seq analysis showed that endothelial markers (e.g., *PECAM1*, *CDH5*, and *CLDN5*) were enriched in the cluster associated with iCE-BECs, whereas cells expressing mural cell markers (e.g., *MYL9*, *TAGLN*, and *ACTA2*) were enriched in iECs and iEC-Rep cells (Figures 2B and S2B). To characterize the cell identity of iCE-BECs, we evaluated the expression of a recently described list of genes (Lu et al., 2021) (see Table S1) associated either with an endothelial transcriptomic signature (high PC1 loading) or an epithelial transcriptomic identity (low PC1 loading). Endothelial signature genes were expressed across protocols and highly enriched in iCE-BECs, whereas epithelial signature genes were mildly expressed across the three protocols (Figure 2C). This transcriptomic signature is consistent with the highly heterogeneous expression of PECAM1 in iECs and could be explained by the presence of non-endothelial cells in the iEC protocol.

To specifically assess the brain identity in endothelial cells across protocols (i.e., independently of the potential presence of contaminating non-endothelial cells), we reanalyzed the data in cells expressing genes associated with endothelial identity (*PECAM1*, *KDR*, *VWF*, *ENG*, *CDH5*, *FLT4*, and *FCGRT*; see supplemental information for details). Within this population, we defined a brain endothelial score based on the expression of genes known to be enriched in brain endothelial cells (Yang et al., 2022) (*CLDN5*, *MFSD2A*, *SLC16A1*, *SLC3A2*, *SLC38A5*, *SLC7A5*, and *SLC2A1*; see supplemental information for details). We found that iCE-BECs had a higher brain endothelial score compared to iEC and iEC-Rep, suggesting that the iCE-BEC protocol improves the acquisition of a brain-like signature specifically in endothelial cells (Figure 2D).

We next performed bulk RNA-seq analysis to do a quantitative comparison of differentially expressed genes across protocols. Principal-component analysis showed a clear separation of the three different protocols, with PC1 accounting to 82.65% of the total variance (Figure 2E). Loading genes in PC1 were shown to be enriched for ECM-related pathways (Figure 2F). In agreement with the single-cell dataset, endothelial and brain endothelial cell markers were upregulated in iCE-BECs compared to iECs and iEC-Rep (Figure 2G), while epithelial and mural cell markers were not detected or strongly downregulated (Figure S2C). iCE-BECs showed an upregulation of *MSX1*, *ZIC3*, *EBF1*, and *APLN*, which are genes specifically

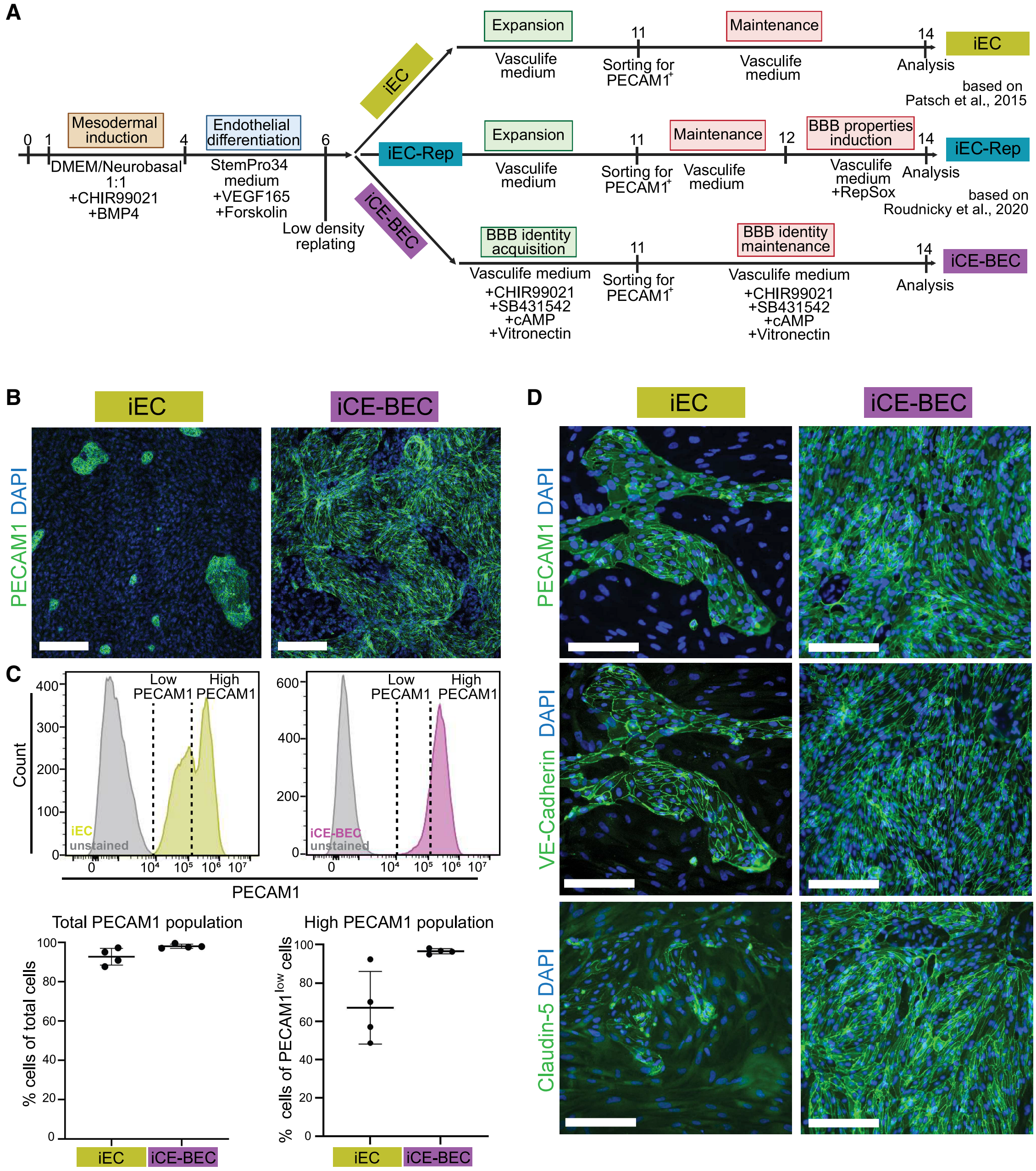

Figure 1. Differentiation of brain endothelial cells from induced pluripotent stem cells by a chemical cocktail and ECM support (iCE-BECs)

(A) Schematic of the protocols used for iPSC differentiation as described in the experimental procedures.

(B) Representative fluorescence images after immunostaining with the endothelial-specific marker PECAM1 of iEC and iCE-BECs before PECAM1 sorting at day 11. Cells are pseudo-colored showing PECAM1 in green and DAPI-stained nuclei in blue. Scale bars, 250 μm.

(C) Representative flow cytometry histograms showing fluorescence intensity of PECAM1 on the *x* axis and the number of cells on the *y* axis from live iEC (yellow) and iCE-BECs (purple) and respective unstained controls (gray) after sorting at day 14. Vertical lines illustrate gating for low PECAM1 and high PECAM1 expression. Graphs show the mean ± SD percentage of PECAM1-positive cells of total cells and highly expressing PECAM1 cells in PECAM1-positive population after sorting at day 14 across four independent differentiations.

(D) Representative fluorescence images after immunostaining with endothelial-specific markers of iEC and iCE-BECs at day 14. PECAM1, VE-Cadherin, or Claudin-5 are pseudo-colored in green and DAPI-stained nuclei in blue. Scale bars, 200 μm.

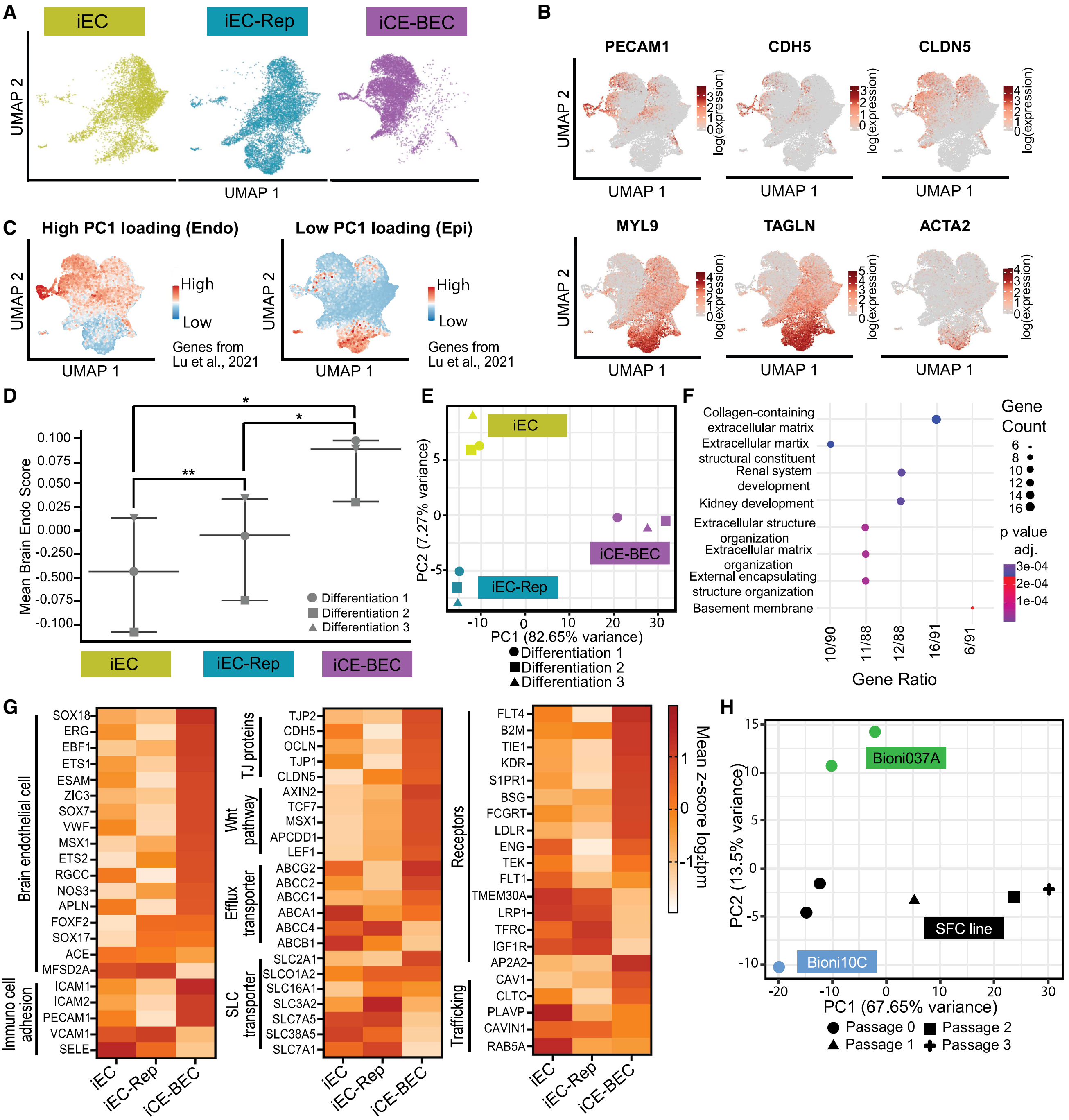

Figure 2. Transcriptomic signature of iCE-BECs

(A) Integrated UMAP plot of scRNA-seq analysis, performed on the endothelial cells generated using the protocols summarized in Figure 1A. Data are generated from three independent differentiations per condition.

(B) Selected feature plots showing normalized expression of marker genes of endothelial (*PECAM1*, *CDH5*, and *CLDN5*) and mural (*MYL9*, *TAGLN*, and *ACTA2*) markers, plotted on the UMAP from Figure 2A.

(C) Feature plots showing expression of endothelial modules (Endo) "high PC1 loading" and epithelial module (Epi) "low PC1 loading" using the gene list described in (Lu et al., 2021) (see Table S1), plotted on the UMAP from 1A.

(legend continued on next page)

enriched in brain endothelial cells in mice (Sabbagh et al., 2018). Additionally, iCE-BECs had an upregulation of genes associated with BBB function, including tight junction genes (*CLDN5*, *TJP1*, *TJP2*, *OCLN*, and *CDH5*), solute carriers (*SLC2A1*, *SLCO1A2*, and *SLC16A1*), efflux transporters (*ABCG2* and *ABCC2*), genes involved in immune cell adhesion (*ICAM1*, *ICAM2*, and *PECAM1*) and downstream effectors of Wnt signaling (*LEF1*, *TCF7*, *AXIN2*, and *APCDD1*) (Figure 2G). These data confirm that iCE-BECs exhibit a brain identity signature and express key genes essential for brain endothelial function. To confirm the reproducibility of the iCE-BEC protocol, we performed bulk RNA-seq in three different iPSC lines and 4 different iCE-BEC passage numbers (see methods for details). Principal component analysis shows that PC1 accounts for almost 68% of the variance and is driven by passage number after differentiation. In contrast, PC2, which is driven by the effects from the different parental lines, accounts for less than 15% of the total variance (Figure 2H). Moreover, just one of the genes associated with the BBB score defined above (*SLC38A5*) is among the top 100 loading genes in PC2 and none in PC1 (Table S2). This analysis confirms that the iCE-BEC protocol generates cells with brain endothelial-like identity across different parental iPSC lines.

To validate the transcriptional changes observed in iCE-BECs, we performed flow cytometry analysis of selected proteins in PECAM1-positive cells (Figures 3A and S2D). We found that VE-Cadherin, vWF, ERG, Claudin-5, GLUT1, and LDL-R expression were upregulated in iCE-BECs compared to iECs. This result confirms the transcriptional data and shows that the iCE-BEC protocol increases the expression of proteins associated with brain identity.

To investigate the applicability of iCE-BECs for transport assays, we measured the expression of transporters and receptors with whole-cell proteomics. We compared these data to the proteome of both primary human brain microvascular endothelial cells (HBMVECs) and the widely used immortalized brain endothelial cell line, HCMEC/D3. We observed small changes in specific receptors that were upregulated (SLC2A1, ABCA1, and ABCG2) or downregulated (SLC7A1 and SLC7A5) in iCE-BECs. However, the proteomics analysis showed an overall similar expression of transporters and receptors between the three cell lines (Figure 3B). We confirmed the expression of SLC2A1, ABCB1, ABCC1, and ABCA1 by immunofluorescence (Figure 3C). SLC2A1 is clearly increased in iCE-BECs using three different parental iPSC lines compared to immortalized and primary brain endothelial cells (Figure 3C). Taken together, transcriptomics and proteomics data demonstrated that iCE-BECs are a homogeneous population of cells with human brain endothelial identity and express proteins relevant for the *in vitro* assessment of transcytosis across the BBB.

iCE-BECs restrict the transport of large molecules and recapitulate receptor-mediated transcytosis

Next, we assessed the permeability of iCE-BECs. First, iCE-BECs showed a 50% increase in TEER values compared to iECs (Figure S3A). Second, permeability measurements on dextrans of different molecular weights (3, 40, and 70 kDa) in transwell chambers was decreased by 75% compared to both iECs and iEC-Rep (Figure S3B). Importantly, since iCE-BECs grew as a continuous monolayer in the transwell filter (Figure S3C), their observed decreased permeability is not due to the formation of multiple pseudo-stratified cell layers in the filter.

The apparent reduction in permeability of iCE-BECs compared to iECs could be explained by heterogeneous iECs with lack of barrier properties. Therefore, to further characterize iCE-BEC permeability, we compared these cells to primary HBMVECs using a microfluidic platform (MIMETAS OrganoPlate) well established to investigate BBB transport (Wevers et al., 2018). As expected, both HBMVECs and iCE-BECs grown on the MIMETAS platform homogeneously expressed endothelial markers and tight junction proteins (Figure 4A). To evaluate paracellular permeability, we measured the flux of fluorescently labeled dextran over time (Figure 4B). We found that the apparent permeability of iCE-BECs to 70 kDa dextran was similar to HBMVECs and in the order of 10^{-7} cm·s^{-1} (Figures 4C and 4D; see supplemental information for details). Treatment of iCE-BECs with vascular endothelial growth factor (VEGF) increased the permeability to dextran, further

(D) Mean ± range Brain Endo Score levels in cells expressing endothelial markers (see supplemental information for details) across the three protocols described in Figure 1A. $**p < 0.01$ and $*p < 0.05$ by one-way ANOVA followed by paired t test comparisons with FDR correction for multiple comparisons.

(E) Principal component analysis (PCA) of bulk RNA-seq comparing iEC, iEC-Rep, and iCE-BECs from three independent differentiations each.

(F) Gene ontology terms enriched in the principal component 1 from the PCA performed in Figure 2E.

(G) Bulk RNA-seq heatmaps showing expression of genes associated with blood-brain barrier properties comparing iECs, iEC-Rep, and iCE-BECs. Values are expressed as mean *Z* score log2tpm, with three independent differentiations per condition.

(H) PCA of iCE-BECs generated from three different iPSC lines (SFC086_03_03, Bioni037A, and Bioni10C) across four passages after differentiation.

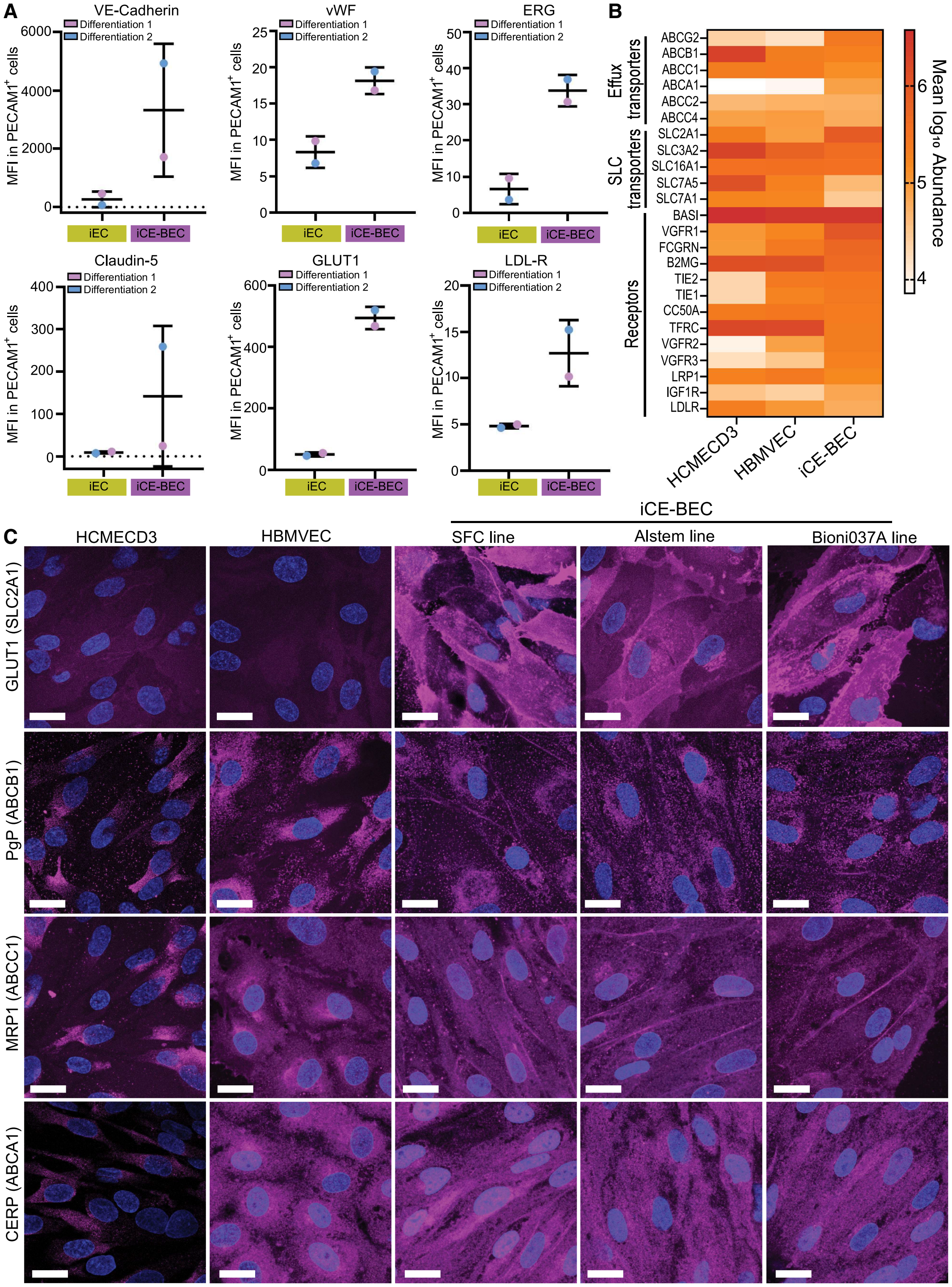

Figure 3. Proteomic signature of iCE-BECs

(A) Flow cytometry measurements of protein expression in PECAM1-positive iEC and iCE-BECs. Median fluorescent intensity (MFI) of each marker was extracted and normalized to the MFI of their respective unstained control in each condition and differentiation. Data come from two differentiations; graphs show mean ± SD, each data point represents an average of a duplicate.

(legend continued on next page)

confirming the endothelial identity of the cells (Figure S3D). We observed similar values in the order of 10^{-7} cm·s^{-1} across four different iPSC lines (Figure 4D). However, the Alstem iPSC line consistently showed higher P_{app} values. These results demonstrate that the low paracellular permeability of iCE-BECs can be recapitulated across multiple iPSC lines.

We next evaluated receptor-mediated transcytosis in iCE-BECs using the Brainshuttle (BrS), a validated monovalent bispecific antibody against TfR1 that effectively crosses the BBB (Grimm et al., 2023). In agreement with previously reported data for the same antibody in BBB organoids (Simonneau et al., 2021), we found that the BrS showed an almost 2-fold higher transport rate compared to a non-targeting immunoglobulin G (IgG) (Figures 4E, 4F, and S3E). This higher transport rate is not due to cell death or disruption of barrier integrity as incubation with the BrS antibody did not increase the permeability of 40 kDa dextran (Figure S3F). The extent of BrS transcytosis was reproducible across three different iPSC lines differentiated with the iCE-BEC protocol (Figure 4G). The lack of observed transcytosis of BrS in the Alstem iPSC line could be due to its higher basal paracellular permeability (Figure 4D). These data show that iCE-BECs recapitulate key features of the BBB, including low permeability to large molecules and receptor-mediated transcytosis. Together, our data show that iCE-BECs (1) have endothelial identity, (2) show a transcriptomic and proteomic brain-endothelial signature, and (3) recapitulate receptor-mediated transcytosis of antibodies. Therefore, we conclude that iCE-BECs are a suitable human *in vitro* system to investigate the regulation of intracellular transport across brain endothelial cells.

ApoE4 impairs endosome maturation in iCE-BECs

ApoE4 is the major risk factor for sporadic AD (Armstrong 2019). Multiple studies show that ApoE4 can affect BBB function, likely via non-cell autonomous mechanisms (Montagne et al., 2020; Barisano et al., 2022). Nevertheless, it is unclear whether ApoE4 affects brain endothelial cells in a cell-autonomous manner (Blumenfeld et al., 2024). To address this, we used two pairs of isogenic iPSC lines with either a homozygous ApoE3 (Bioni037-A, Alstem iPS26) or a homozygous ApoE4 (Bioni037-A4, Alstem iPS16) gene variant and differentiated these into iCE-BECs. We refer here to the Bioni037 lines as isogenic Pair 1 and the Alstem lines as isogenic Pair 2. Importantly, ApoE4 did not change the proliferation rate of iPSCs (Figure S4) and did not impair the differentiation process as evidenced by expression of endothelial and tight junction genes (Figure S4). Both isogenic pairs expressed ApoE with an apparent mild mRNA and protein reduction in the ApoE4 lines (Figures S4E–S4G). These data suggest that ApoE4 iCE-BECs can be used to investigate cell-autonomous effects of ApoE4.

Recent work showed that ApoE4 alters multiple endocytosis mechanisms *in vivo* (Nuriel et al., 2017; Barisano et al., 2022). Therefore, we analyzed the organization of endosomes in ApoE4 iCE-BECs in both isogenic pairs. We found that the number of early endosomes (labeled by EEA1) was higher in ApoE4 compared to ApoE3 iCE-BECs (Figures 5A and 5C). In addition, the total amount of EEA1 per endosome was also higher in ApoE4 compared to ApoE3 iCE-BECs (Figure 5B). We confirmed by transmission electron microscopy that ApoE4 iCE-BECs had an increase in endosome number and size compared to ApoE3 cells (Figure 5D). The changes in endosome number, size, and EEA1 amount in ApoE4 iCE-BECs could reflect impaired endosome maturation. To test this hypothesis, we used live-cell imaging to measure both endosomal pH and endosome sorting tubule biogenesis. We assessed endosomal pH by measuring the fluorescence intensity of transferrin (Tf) conjugated with a pH-sensitive dye in individual endosomes. The intensity per endosome was normalized to the fluorescence intensity from Tf conjugated to a non-pH sensitive dye. We found that ApoE4 iCE-BECs had increased signal for each of the Tf-fluorescent conjugates but a lower fluorescence ratio (Figures 5E and 5F). This result points to a higher uptake of Tf and to a higher endosomal pH (i.e., less acidic) in ApoE4 iCE-BECs. Next, we visualized the dynamics of fluorescent Tf to evaluate the formation of sorting tubules. In agreement with previous data on mouse brain endothelial cells (Villaseñor et al., 2017), we observed frequent events of sorting tubule biogenesis and vesicle fission in ApoE3 iCE-BECs (Figure 5G; Videos S1 and S2). In contrast, the number of sorting tubules was substantially reduced in ApoE4 iCE-BECs (Figure 5H). Together, these data show that ApoE4 impairs endosomal maturation, which is reflected by enlarged, less acidic endosomes with reduced sorting.

ApoE4 increases TfR1 expression without altering its transport rate in iCE-BECs

We next asked whether changes in endosome maturation led to functional consequences in intracellular transport

(B) Whole-cell proteomics heatmap showing expression of brain endothelial receptors and transporters comparing immortalized human brain endothelial cells (HCMEC/D3, *n* = 3), primary human microvascular brain endothelial cells (HBMVEC, *n* = 6), and iCE-BECs (*n* = 4 independent differentiations). Values are expressed as mean log10 abundance.
(C) Representative fluorescence images after immunostaining for SLC2A1, ABCB1, ABCC1, or ABCA1 in HCMEC/D3, HBMBVECs, and iCE-BECs from three different iPSC lines. Transporters are pseudo-colored in magenta and DAPI-stained nuclei in blue. Scale bars, 20 μm.

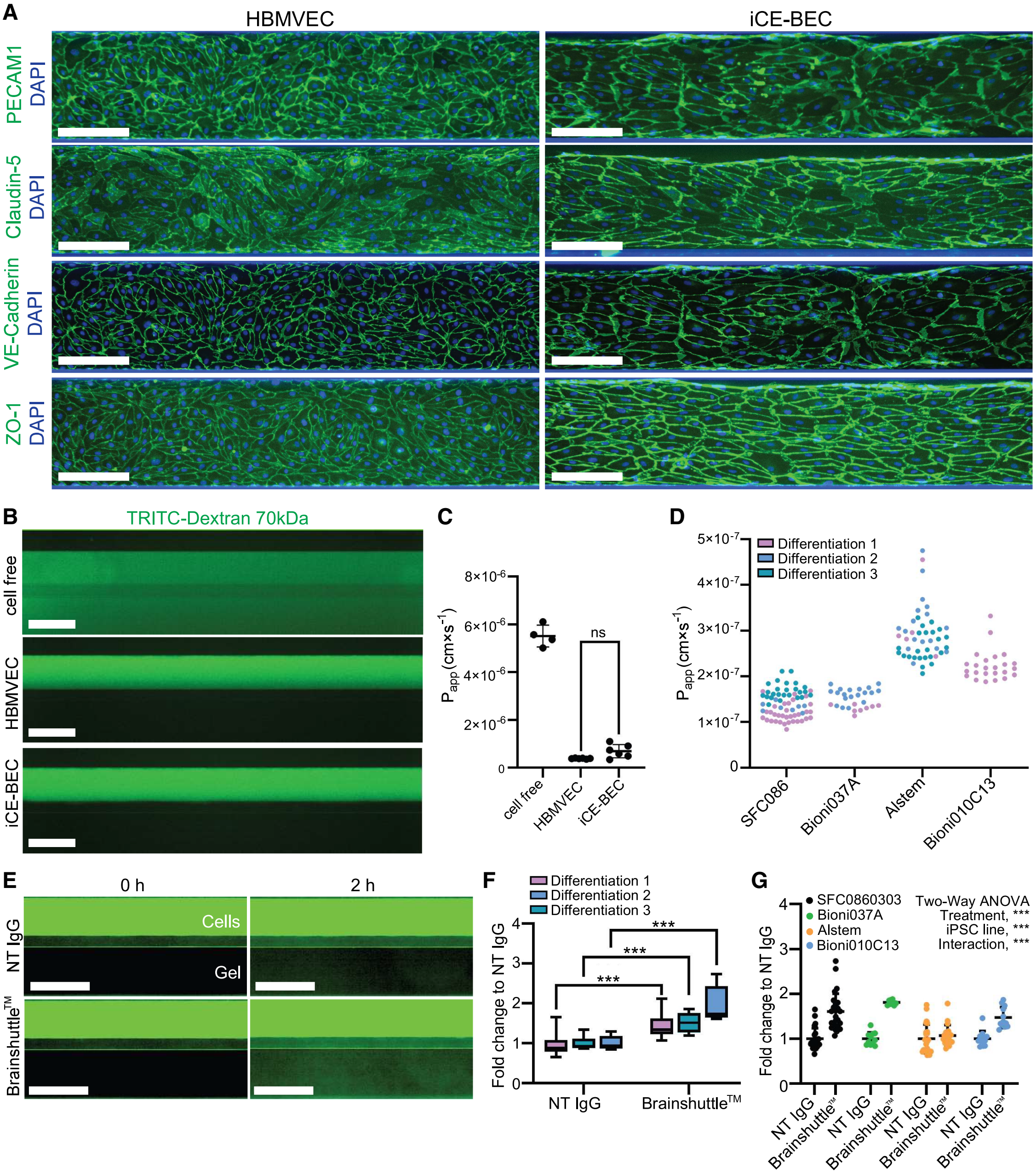

Figure 4. iCE-BECs show low permeability to large molecules and recapitulate receptor-mediated transcytosis of Brainshuttle antibodies

(A) Representative fluorescence images after immunostaining with PECAM1, Claudin-5, VE-Cadherin, and ZO-1 (pseudo-colored in green) of HBMVECs and iCE-BECs grown in a MIMETAS OrganoPlate 3-lane 96. Scale bars, 100 μm.

(B) Representative fluorescence images of MIMETAS OrganoPlate 2-lane chambers comparing cell-free (top), HBMVECs (middle), and iCE-BECs (bottom) after incubation with 70 kDa TRITC-dextran for 40 min. Scale bars, 100 μm.

(C) Quantification of apparent permeability (P_{app}) of cell-free, HBMVECs, and iCE-BECs to 70 kDa TRITC-dextran. Graph shows mean ± SD. ns, not statistically significant by Kruskal-Wallis test with Dunn's multiple comparisons test; $p = 0.146$ with $n = 4$ independent chips from one lot of primary cells (HBMVECs) and one differentiation (iCE-BECs).

(D) Quantification of P_{app} of iCE-BECs to 40 kDa dextran using MIMETAS OrganoPlate 2-lane 96 in iCE-BECs generated with different parental iPSC lines. Graph shows data from three differentiations; each data point represents one chip.

(legend continued on next page)

rates. To evaluate this, we followed the uptake and recycling of fluorescently labeled Tf in iCE-BECs from both isogenic pairs using a continuous pulse or a pulse-chase experimental design (Figures S5A and S5B). ApoE4 iCE-BECs had a 50% higher total amount of internalized Tf compared to ApoE3 iCE-BECs (Figure 6A). This increase in total internalized Tf was confirmed by flow cytometry (Figure S5C). However, the rate of Tf internalization was similar between ApoE4 and ApoE3 iCE-BECs (Figure 6C). Similarly, the total amount of Tf after a 20-min pulse was higher in ApoE4 compared to ApoE3 iCE-BECs (Figure 6B), but the recycling rate was similar between the two genetic variants (Figure 6D). An increased Tf uptake capacity without changes to the internalization and recycling rates could be explained by the upregulation of TfR1 expression. Indeed, we found that both mRNA and protein TfR1 expression were upregulated in ApoE4 compared to ApoE3 iCE-BECs (Figures 6E–6I). Note that western blots did not reflect increased TfR expression in ApoE4 cells. These data show that ApoE4 leads to TfR1 upregulation but does not alter intracellular transport rates in iCE-BECs.

ApoE4 alters iron metabolism homeostasis in iCE-BECs

TfR1 expression is regulated by iron responsive proteins that sense iron levels in the cytoplasm (Anderson and Frazer 2017). In iron-deficient cells, iron-responsive proteins upregulate TfR1 mRNA by binding to its 3′UTR, thus preventing its degradation. We therefore tested whether ApoE4 iCE-BECs had lower cytosolic iron levels in both isogenic pairs. To measure the intracellular labile iron pool, we used FerroOrange, a fluorescent probe that specifically detects labile iron (II) ions ($Fe2^{+}$) in live cells (Figure S6A). With this method, we found that ApoE4 iCE-BECs had a reduced labile iron pool compared to ApoE3 cells (Figures 7A and 7B). We confirmed this result with an orthogonal method that measures the intracellular iron pool with the metal-sensitive calcein-acetoxymethyl ester (Calcein-AM). This method confirmed a reduction in the labile iron pool in ApoE4 compared to ApoE3 iCE-BECs (Figures S6B–S6D).

The changes in the iron labile pool in iCE-BECs suggest that the effect of ApoE4 broadly impact iron metabolism. Therefore, we evaluated proteins regulated by iron-responsive elements: ferritin (ferritin heavy chain, FTH; iron storage) and divalent metal transporter 1 (DMT-1; iron import). It is well documented that low cytosolic iron downregulates FTH while upregulating DMT-1 (Wallander et al., 2006). We found that ferritin protein and mRNA expression (Figures 7C–7G and 7I) were downregulated, whereas DMT-1 protein and mRNA (Figures 7C–7F, 7H, and 7J) were upregulated in ApoE4 compared to ApoE3 iCE-BECs. Note, however, that the effect size for mRNA and protein via western blot analysis was highly variable between isogenic pairs. This points to a potential effect of the genetic background on intracellular iron levels. Altogether, our data show that ApoE4 alters iron cytoplasmic levels and leads to expression changes across the iron transport pathway in iCE-BECs.

In conclusion, ApoE4 iCE-BECs exhibited impaired maturation of endosomes and disrupted iron homeostasis, characterized by lower intracellular iron levels and altered expression of iron-related genes. These findings highlight the importance of the ApoE4 gene variant in modulating intracellular trafficking and iron metabolism in brain endothelial cells.

DISCUSSION

Here, we describe a protocol to generate iCE-BECs that shows (1) a brain endothelial transcriptomic and proteomic signature, (2) functional barrier properties, and (3) recapitulate receptor-mediated transcytosis. Importantly, these three features were reproduced across multiple iPSC parental lines. The iCE-BEC protocol shares similarities with the recently described cARLA differentiation method (Porkoláb et al., 2024) and thus confirms the importance

(E) Representative fluorescence images of iCE-BECs grown on MIMETAS OrganoPlate 2-lane chambers comparing iEC (top) and iCE-BECs (bottom) after incubation with 200 nM non-targeting IgG (NT IgG) or a Brainshuttle antibody. Images were acquired immediately after incubation (0 h) or after 2 h. Scale bars, 500 μm.
(F) Quantification of relative antibody transcytosis across iCE-BECs. Individual values are normalized to the mean of the NT-IgG condition. Graph shows boxplots with interquartile ranges and medians. Lines show the 5th and 95th percentiles. $***p < 0.001$, no significant effect of differentiation on transcytosis, significant interaction of treatment and differentiation; $**p < 0.01$ by a mixed effect analysis with Sidak's multiple comparisons with $n = 3$ independent differentiations with at least seven independent chips per condition.
(G) Quantification of relative antibody transcytosis across iCE-BECs generated with different parental iPSC lines after incubation with 200 nM non-targeting IgG (NT IgG) or Brainshuttle antibody. Graph shows mean ± SD of multiple differentiations. Each data point represents one chamber. Significant main effect of treatment on transcytosis, $***p < 0.001$; significant main effect of iPSC line on transcytosis, $***p < 0.001$; and significant interaction between iPSC line and treatment, $***p < 0.001$ by two-way ANOVA with $n = 3$ (SFC086), $n = 2$ (Bioni037A), $n = 3$ (Alstem), and $n = 1$ (Bioni010C13) independent differentiations with at least eight independent chips per condition.

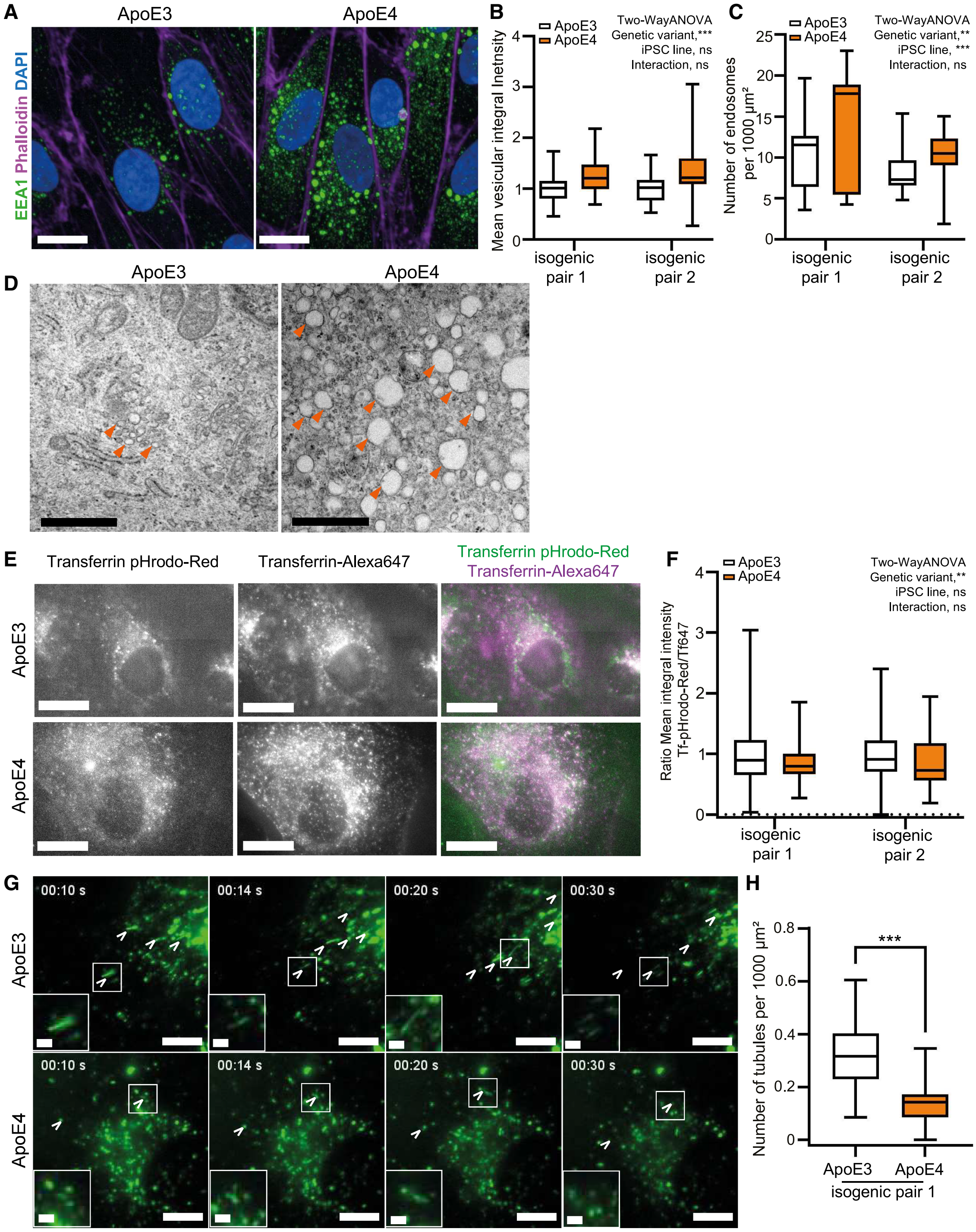

Figure 5. ApoE4 alters early endosome maturation in iCE-BECs

(A) Representative maximum intensity projections images of iCE-BECs with ApoE3 or ApoE4 genetic variant showing EEA1 pseudo-colored in green, Phalloidin in magenta, and DAPI-stained nuclei in blue. Scale bars, 10 μm.

(B and C) Quantification of mean integral vesicular intensity of EEA1 normalized to Phalloidin-positive area (B) or number of EEA1-positive endosomes per 1,000 μm^2 Phalloidin-positive area in both isogenic pairs (C). Individual values in (B) were normalized to the mean of ApoE3 conditions for each experiment. Significant main effect of genetic variant on EEA1 intensity, $***p < 0.001$, no significant main effect of iPSC line on EEA1 intensity and no significant interaction between iPSC line and genetic variant. Significant main effect of genetic

(legend continued on next page)

of Wnt/cAMP activation and TGF-β inhibition to robustly induce brain endothelial identity and barrier properties of endothelial cells *in vitro*. In comparison with the cARLA differentiation method, iCE-BECs show increased expression of ZIC3 and FOXF2, two transcription factors specifically enriched in brain endothelial cells *in vivo* and important to induce barrier properties in endothelial cells (Patel et al., 2024). Importantly, iCE-BECs showed the same extent of BrS antibody transcytosis observed in a BBB organoid model (Simonneau et al., 2021). We therefore consider iCE-BECs as a suitable model to investigate receptor-mediated transcytosis across the BBB.

The ApoE4 genetic variant is the major risk factor for sporadic AD heavily affecting the onset of the disease (Armstrong 2019). Extensive evidence documents the impact of ApoE4 on BBB permeability and function (Halliday et al., 2016; Montagne et al. 2020, 2021; Yamazaki et al., 2020). However, a recent study using iPSC-derived brain endothelial-like cells found that the effect of ApoE4 on BBB permeability is likely not cell-autonomous (Ding et al., 2024). In this manuscript, we show that ApoE4 expression in brain endothelial cells leads to both impaired endosome maturation and altered iron homeostasis. This finding expands the role of ApoE4 at the BBB beyond paracellular permeability to include cell-autonomous regulation of intracellular transport.

Previous data using ApoE4 knockin mice showed an increase in TfR1 expression in brain endothelial cells (Barisano et al., 2022), similar to the effect that we see in iCE-BECs. Our data suggest that TfR1 upregulation is a consequence of lower cytoplasmic iron levels in ApoE4 cells. We propose a model to explain how impaired endosomal maturation observed in ApoE4 BECs leads to lower cytosolic iron levels (Figure S7). First, ApoE4 triggers increased endosomal pH. Second, increased endosomal pH leads to reduced dissociation of ferric iron from Tf. Third, sustained ferric iron binding to Tf prevents DMT-1-mediated transport into the cytosol. Together, these events would lead to an iron-depleted cytosol and trigger expression changes of iron-related genes, including TfR1.

How ApoE4 leads to changes in early endosome maturation remains to be clarified. Transcriptomic analysis performed on brains of ApoE4 mice revealed a significant upregulation of genes involved in the regulation of endosomal-lysosomal pathway, including Rab5b, Rab7, Snx3, Snx15, Vps4a, Vps24, and Vps29, and suggested an ApoE4-specific trafficking and sorting dysregulation (Nuriel et al., 2017). Interestingly, our data demonstrating increased endosomal pH is opposite to observations in iPSC-derived astrocytes, where the ApoE4 gene variant showed enlarged early endosomes but decreased endosomal pH (Prasad and Rao 2018). The same study found that NHE6 activity could normalize endosomal pH in iPSC-derived astrocytes. Follow-up studies with iCE-BECs could help to dissect the role of NHE6 or other molecular mechanisms on endosomal maturation in the context of ApoE4. Overall, our findings on the influence of ApoE4 on endosomes and TfR1 expression at the BBB will be instrumental in refining strategies that leverage intracellular transport mechanisms for the delivery of therapeutic antibodies to the brain.

Limitations of the study

Model development and characterization was performed using four iPSC lines from different donors, and the findings on ApoE4 were observed in two isogenic iPSC pairs. Validation of the findings would be strengthened by replicating the findings in iPSC lines from a larger and diverse pool of donors. Ultimately, however, the translatability of

variant on endosome number, $^{**}p < 0.01$; significant main effect of iPSC line on number of endosomes, $^{***}p < 0.001$; no significant interaction between iPSC line and genetic variant by two-way ANOVA with $n = 3$ independent differentiations with approximately 200 cells per condition.

(D) Representative transmission electron microscopy images of ApoE3 or ApoE4 iCE-BECs in isogenic pair 1. Arrowheads point to endosomes. Scale bars, 1 μm.

(E) Representative images of transferrin in live iCE-BECs with ApoE gene variants after incubation with 25 μg/mL pH-sensitive Transferrin pHrodo Red and 25 μg/mL Transferrin-Alexafluor647 for 10 min. Scale bars, 20 μm.

(F) Quantification of the ratio between Transferrin pHrodo Red (Tf-pHrodo-Red) and Transferrin-Alexafluor647 (Tf647) per endosome. Significant main effect of genetic variant on the ratio, $^{**}p < 0.01$; no significant main effect of iPSC line on the ration; and no significant interaction between iPSC line and genetic variant on the ratio by two-way ANOVA with $n = 3$ independent differentiations with 30 cells per experiment for each isogenic pair.

(G) Representative images of Tf (green) intracellular transport in live iCE-BECs with ApoE gene variants in isogenic pair 1 after incubation with 25 μg/mL of Tf-Alexafluor488 for 3 h. The time stamp shows the elapsed time in seconds after the acquisition of the first image. Arrowheads point to individual sorting tubules across time frames. Inserts depict a zoom-in on selected sorting tubules. Scale bars, 10 μm, inserts 2 μm.

(H) Quantification of the mean number of sorting tubules per 1,000 μm^2 occurring in one minute. $^{***}p < 0.001$ by Student's t test with $n = 3$ independent differentiations with 20 videos per experiment from isogenic pair 1. All graphs show boxplots with interquartile ranges and medians. Lines show the 5th and 95th percentiles.

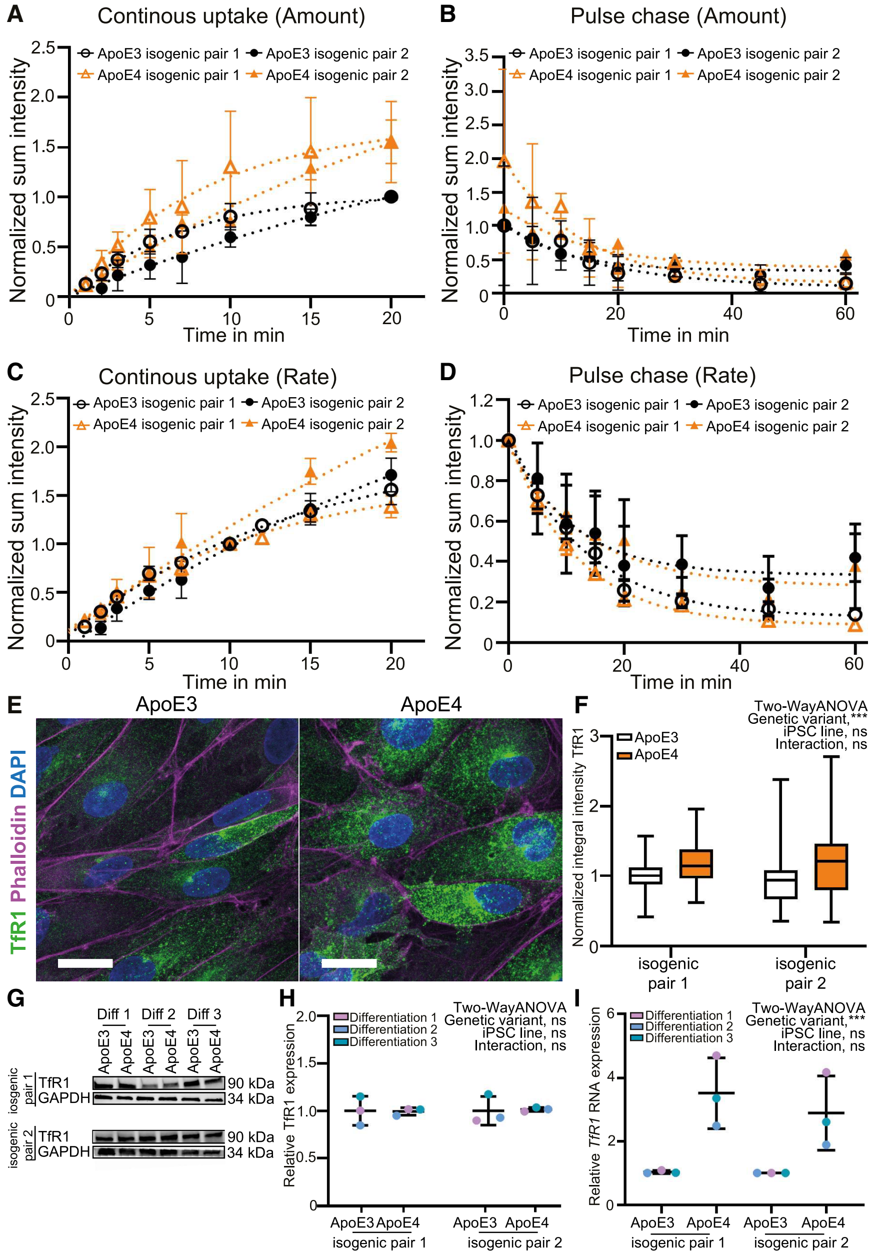

Figure 6. ApoE4 iCE-BECs have increased TfR1 expression but no changes to trafficking kinetics

(A–D) Time courses of continuous uptake (A) or pulse-chase (B) assays showing total vesicular intensity of Tf in ApoE3 or ApoE4 iCE-BECs in both isogenic pairs. Intensity values were divided by the mean of the transferrin intensity at 20 min (A) or immediately after the pulse (B) in ApoE3 iCE-BECs, in each isogenic pair. Points show the average, and error bars show the SEM from 50 images per experiment in four independent differentiations for isogenic pair 1 and three independent differentiations for isogenic pair 2 (A) or six independent differentiations for isogenic pair 1 and three independent differentiations for isogenic pair 2 (B). Lines show the best exponential fit for the experimental data. Graphs in (C and D) were normalized to the transferrin intensity at 10 min in each condition in (C) and immediately after the pulse in each condition in (D).

(E) Representative maximum intensity projection images of iCE-BECs with ApoE3 or ApoE4 genetic variant showing TfR1 pseudo-colored in green, Phalloidin in magenta, and DAPI-stained nuclei in blue. Scale bars, 20 μm.

(F) Quantification of TfR1 integrated intensity in Phalloidin-positive area in both isogenic pairs. Intensity values for each experiment were normalized to ApoE3 conditions. Graph shows boxplots with interquartile ranges and median. Lines show the 5th and 95th percentiles. Significant main effect of genetic variant on TfR1 intensity, $***p < 0.001$; no significant main effect of iPSC line on TfR1 intensity; and no significant interaction between iPSC line and genetic variant by two-way ANOVA with $n = 3$ independent differentiations with approximately 200 cells per experiment.

(G) Representative immunoblot detecting TfR1 with GAPDH as a loading control showing three independent differentiations (Diff) for the two isogenic pairs.

(H) Quantification of relative TfR1 protein expression of immunoblot in (G). Graphs show mean ± SD. Points represent independent differentiations. No significant main effect of genetic variant or iPSC line on TfR1 expression and no significant interaction between iPSC line and genetic variant by two-Way ANOVA with $n = 3$ independent differentiations.

(I) Quantification of relative *TfR1* mRNA expression by quantitative PCR. Graph shows mean ± SD. Points represent independent differentiations. $***p < 0.001$, no significant main effect of iPSC line on TfR1 intensity and no significant interaction between iPSC line and genetic variant by two-Way ANOVA with $n = 3$ independent differentiations with three technical replicates per experiment from both isogenic pairs.

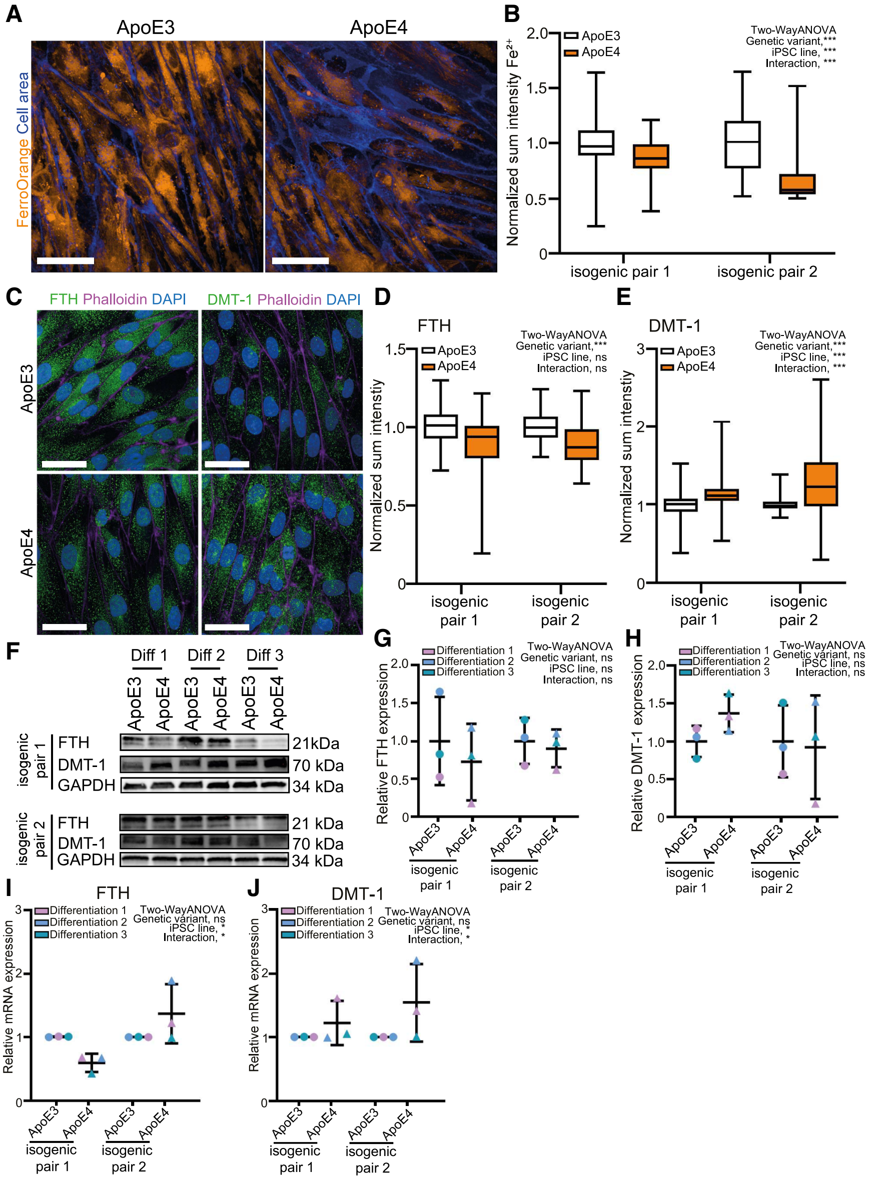

Figure 7. ApoE4 reduces intracellular iron levels and alters iron transport pathways in iCE-BECs

(A) Representative maximum intensity projection images of iCE-BECs with ApoE3 or ApoE4 genetic variants after labeling labile iron (II) ions (Fe^{2+}) with FerroOrange pseudo-colored in orange and cell area labeled by CytoTrace Green in blue. Scale bars, 50 μm.

(B) Quantification of sum intensity of FerroOrange intensity normalized to the total cell area per image. Intensity values were normalized to data from ApoE3 iCE-BECs per experiment. Significant main effect of genetic variant on FerroOrange intensity, $***p < 0.001$; significant main effect of iPSC line on FerroOrange intensity, $p < 0.001$; and significant interaction between iPSC line and genetic variant, $***p < 0.001$ by two-way ANOVA with $n = 3$ independent differentiations with 40 images per experiment from both isogenic pairs.

(C) Representative maximum intensity projection images of iCE-BECs with ApoE3 or ApoE4 genetic variant after immunostaining for FTH and DMT-1 (green) and actin (phalloidin, magenta). DAPI-stained nuclei are shown in blue. Scale bars, 100 μm.

(D and E) Quantification of FTH (D) and DMT-1 (E) sum intensity in Phalloidin-positive area. Intensity values were normalized to data from ApoE3 iCE-BECs for each experiment. Significant main effect of genetic variant on FTH intensity, $p < 0.001$; no significant main effect of iPSC line on FTH intensity; and no significant interaction between iPSC line and genetic variant. Significant main effect of genetic variant on DMT-1 intensity, $p < 0.001$; significant main effect of iPSC line on DMT-1 intensity, $p < 0.001$; and significant interaction between iPSC line and genetic variant, $p < 0.001$ by two-way ANOVA with $n = 3$ independent differentiations with approximately 800 cells per experiment from both isogenic pairs. Graphs in (B, D, and E) show boxplots with interquartile ranges and medians. Lines show the 5th and 95th percentiles.

(F) Representative immunoblot detecting FTH and DMT-1 with GAPDH as a loading control showing three independent differentiations (Diff) for the two isogenic pairs.

(G and H) Quantification of relative FTH (G) and DMT-1 (H) protein expression of immunoblot in (F). Graphs show mean ± SD. Points represent independent differentiations. No significant main effect of genetic variant or iPSC line on FTH or DMT-1 expression and no significant interaction between iPSC line and genetic variant by two-way ANOVA with $n = 3$ independent differentiations.

(I and J) Quantification of relative mRNA expression by quantitative PCR of ferritin heavy chain (FTH) and Divalent metal transporter 1 (DMT-1). Graph shows mean ± SD. Points represent independent differentiations. No significant main effect of genetic variant on FTH expression; significant main effect of iPSC line on FTH expression, $*p < 0.05$; and significant interaction between iPSC line and genetic variant, $*p < 0.05$; no significant main effects of genetic variant or iPSC line on DMT-1 expression; no interaction between iPSC line and genetic variant by two-way ANOVA with $n = 3$ independent differentiations with three technical replicates per experiment.

the phenotypes described in iCE-BECs requires confirmation in patients.

METHODS

hiPSC lines

Unless indicated differently in each figure, all experiments were performed differentiating endothelial cells from hiPS_SFC086_03_03 line. hiPS_SFC086_03_03 were established by reprogramming skin fibroblasts with Sendai virus (CytoTune v II kit) at STEMBancc from a female donor. Karyotype analysis was performed by WiCell, and no abnormalities were detected. To assess the reproducibility of the protocol, BIONi010-C13 (European Bank for induced pluripotent stem cells, ebisc.org), Alstem iPS26 (see below), and BIONi037-A (see below) were used. For ApoE experiments, Bioni037-A (16423, homozygous ApoE3 gene variant) and Bioni037-A4 (I40-53, homozygous ApoE4 gene variant), referred as isogenic pair 1, and Alstem line iPS16 (homozygous ApoE4 gene variant) and iPS26 (homozygous ApoE3 gene variant), referred as isogenic pair 2, were used. All cell lines were tested for mycoplasma contamination.

hiPSC differentiation into iCE-BECs

Human induced pluripotent stem cells (hiPSCs) were maintained under standard culturing conditions using plates coated with Geltrex (A1413301, Thermo Fisher) and mTeSR Plus Media (100-0276, Stemcell). Passaging was performed using Gentle Cell Dissociation Reagent (100-0485, Stemcell). To differentiate iCE-BECs, 2 million hiPSCs were seeded in a 10-cm dish coated with Geltrex in 10 mL of mTeSR Plus Media supplemented with ROCK inhibitor, Y-27632 10 μM (SCM075, EMD Millipore). Twenty-four hours later, the media was replaced with mesodermal induction media composed of DMEM/F12 (31331-028, Gibco) and Neurobasal medium (21103-049, Life Technologies) 1:1, 2-Mercaptoethanol (31350-10, Thermo Fisher), B27 (17504044, Gibco), N2 (17502048, Gibco) supplemented with fresh CHIR-99021 8 μM (13122, Cayman), and BMP4 25 ng/mL (120-05ET, PeproTech). On days 4 and 5, media was replaced with endothelial differentiation medium consisting of StemPro-34 SFM Media (10639011, Life Technologies) with StemPro-34 supplement, Glutamax (35050061, Gibco), and Penicillin-Streptomycin (15070063, Gibco) and freshly supplemented with VEGF165 50 ng/mL (293-VE-010, R&D) and Forskolin 2 μM (ab120058, Abcam). On day 6 of culture, cells were replated in 10 cm dishes (1.2 million per dish) coated with vitronectin 2.5 μg/mL (SRP3186, Sigma) and fibronectin 7.5 μg/mL (F0895, Sigma) in BBB Identity Acquisition media consisting of VascuLife VEGF Endothelial Medium (LL-0003, Lifeline Cell Technology) supplemented with iCell endothelial cells medium supplement (M1019, FujiFilm Cellular Dynamics) instead of the FBS included in the LL-0003 kit and 10 mL of L-Glutamine LifeFactor instead of the 25 mL included in the kit. The media was freshly supplemented with CHIR-99021 4 μM (13122, Cayman), SB-431542 5 μM (72234, Stemcell), and cAMP 50 nM (ab120424, Abcam).

Cells were cultured in these conditions until day 11, changing media every 2 or 3 days. On day 11 of culture, PECAM1-positive cells were MACS-sorted according to the manufacturer's protocol using CD31 MicroBead Kit from MACS Miltenyi Biotec (130-091-935, Miltenyi Biotec). After MACS, cells were either frozen in liquid nitrogen or replated in the same conditions with BBB identity maintenance media (same composition of BBB Identity Acquisition media). For all experiments performed with iCE-BECs, plates and dishes were coated with vitronectin and fibronectin prior to cell seeding, and cells were cultured in BBB identity maintenance media.

For iEC and iEC-Rep comparison (schematic in Figure 1A), on day 6, progenitor cells were replated on fibronectin-coated dishes and cultured in expansion media. Expansion media consisted of VascuLife VEGF Endothelial Medium (LL-0003, Lifeline Cell Technology) supplemented with iCell endothelial cells medium supplement (M1019, FujiFilm Cellular Dynamics) instead of the FBS included in the kit and 10 mL of L-Glutamine LifeFactor instead of the 25 mL included in the kit (without CHIR-99021, SB-431542, and cAMP). After MACS on day 11, iECs were cultured in the same conditions (maintenance media). For the iEC-Rep condition, the media was supplemented with RepSox 10 μM (73794, Stemcell) 48 h before performing the analysis.

Experiments were carried out on day 14 (3 days after MACS sorting) and with iCE-BECs differentiated from the hiPS_SFC086_03_03 parental line, if not indicated differently.

Statistical analysis

Statistical analyses were performed using GraphPad Prism 10.2.2 software. Normality of the data was evaluated by the Shapiro-Wilk test. For normally distributed numeric data, two-way ANOVA was used to evaluate the effects of genotype and parental cell line in experimental readouts, one-way ANOVA to compare across multiple groups, and Student's t test for comparisons between two groups. For datasets not distributed normally, non-parametric Kruskal-Wallis test was used to compare between multiple groups or Mann-Whitney U test was used for comparisons between two groups. The details for each statistical test are indicated in each figure legend. Data are reported as mean ± SD.

RESOURCE AVAILABILITY

Lead contact

Requests for further information or more detailed protocols should be directed to and will be fulfilled by the corresponding author, Roberto Villaseñor (roberto.villasenor_solorio@roche.com).

Material availability

This study did not generate new unique reagents.

Data and code availability

- The mass spectrometry proteomics data have been deposited to the ProteomeXchange Consortium via the PRIDE partner repository with the dataset identifier PXD062808.
- The data discussed in this publication have been deposited in NCBI's Gene Expression Omnibus and are accessible through GEO Series accession number GSE280214 (scRNA seq across conditions), GSE296358 (bulk RNA-seq across conditions), and GSE296377 (bulk RNA-seq across parental lines and passages).

ACKNOWLEDGMENTS

We thank Dr. Sybille Seiler, Dr. Urs Langen, and Dr. Colette Bichsel for scientific discussions and suggestions. We would like to thank Giacomo Valsecchi, Lena Jutz, Petra Stäuble, Telma Lopes, Lorena Fabella, Dr. Heloise Ragelle, Alena Spielmann, and Pamela Strassburger for excellent technical support. We would like to thank Dr. Udo Hetzel and Barbara Prähauser for their support with the transmission electron microscopy. This collaboration project is co-funded by the PPP Allowance made available by Health-Holland and Top Sector Life Sciences & Health through the PPP program Brains (by Alzheimer Nederland, EpilepsieNL, Hersenstichting Nederland, and MS Research), to stimulate public-private partnerships (IMM-BBB, PPS-BR-2023-01).

AUTHOR CONTRIBUTIONS

L.B. contributed to project administration, conceptualization, data curation, methodology, investigation, visualization, writing—original draft, and writing—review & editing.

N.S.-R. contributed to conceptualization, supervision, and writing—review & editing.

C.S. contributed to conceptualization, supervision, data curation, methodology, investigation, and writing—review & editing. S.C., S.R., A.R., T.M., A.A., B.H., L.D.A., K.S., D.V.T., J.F.-P., N.R.W., X.M.S., C.Zanini., C.Zundel., and S.G. contributed to data curation, methodology, investigation, and writing—review & editing.

L.F. contributed to conceptualization, writing—review & editing, A.O. contributed to writing—review & editing, M.P. contributed to project administration, supervision, conceptualization, data curation, methodology, investigation, visualization, writing—original draft, and writing—review & editing and decision to submit, R.V. contributed to project administration, supervision, conceptualization, data curation, methodology, visualization, writing—original draft, and writing—review & editing and decision to submit.

DECLARATION OF INTERESTS

L.B., N.S.-R., C.S., A.A., S.C., S.R., A.R., T.M., B.H., L.D.A., J.F.-P., K.S., D.V.T., C.Zanini., C.Zundel., L.F., S.G., M.P., and R.V. were employees and shareholders of F. Hoffmann-La Roche Ltd at the time the work was completed. X.M.S. and N.R.W. are employees of MIMETAS BV. OrganoPlate, OrganoReady, OrganoFlow, and OrganoTEER are registered trademarks of MIMETAS BV.

SUPPLEMENTAL INFORMATION

Supplemental information can be found online at https://doi.org/10.1016/j.stemcr.2025.102607.

Received: October 26, 2024
Revised: July 28, 2025
Accepted: July 28, 2025
Published: August 21, 2025

REFERENCES

Abbott, N.J. (2013). Blood-brain barrier structure and function and the challenges for CNS drug delivery. J. Inherit. Metab. Dis. *36*, 437–449.

Anderson, G.J., and Frazer, D.M. (2017). Current understanding of iron homeostasis. Am. J. Clin. Nutr. *106*, 1559S–1566S.

Armstrong, A.R. (2019). Risk factors for Alzheimer's disease. Folia Neuropathol. *57*, 87–105.

Ayloo, S., Lazo, C.G., Sun, S., Zhang, W., Cui, B., and Gu, C. (2022). Pericyte-to-endothelial cell signaling via vitronectin-integrin regulates blood-CNS barrier. Neuron *110*, 1641–1655.e6.

Barisano, G., Kisler, K., Wilkinson, B., Nikolakopoulou, A.M., Sagare, A.P., Wang, Y., Gilliam, W., Huuskonen, M.T., Hung, S.-T., Ichida, J.K., et al. (2022). A "multi-omics" analysis of blood–brain barrier and synaptic dysfunction in APOE4 mice. J. Exp. Med. *219*, e20221137.

Bien-Ly, N., Yu, Y.J., Bumbaca, D., Elstrott, J., Boswell, C.A., Zhang, Y., Luk, W., Lu, Y., Dennis, M.S., Weimer, R.M., et al. (2014). Transferrin receptor (TfR) trafficking determines brain uptake of TfR antibody affinity variants. J. Exp. Med. *211*, 233–244.

Blanchard, J.W., Bula, M., Davila-Velderrain, J., Akay, L.A., Zhu, L., Frank, A., Victor, M.B., Bonner, J.M., Mathys, H., Lin, Y.-T., et al. (2020). Reconstruction of the human blood–brain barrier in vitro reveals a pathogenic mechanism of APOE4 in pericytes. Nat. Med. *26*, 952–963.

Blumenfeld, J., Yip, O., Kim, M.J., and Huang, Y. (2024). Cell type-specific roles of APOE4 in Alzheimer disease. Nat. Rev. Neurosci. *25*, 91–110.

Ding, Y., Palecek, S.P., and Shusta, E.V. (2024). iPSC-derived blood-brain barrier modeling reveals APOE isoform-dependent interactions with amyloid beta. Fluids Barriers CNS *21*, 79.

Grimm, H.P., Schumacher, V., Schäfer, M., Imhof-Jung, S., Freskgård, P.O., Brady, K., Hofmann, C., Rüger, P., Schlothauer, T., Göpfert, U., et al. (2023). Delivery of the Brainshuttle™ amyloid-beta antibody fusion trontinemab to non-human primate brain and projected efficacious dose regimens in humans. mAbs *15*, 2261509.

Halliday, M.R., Rege, S.V., Ma, Q., Zhao, Z., Miller, C.A., Winkler, E. A., and Zlokovic, B.V. (2016). Accelerated pericyte degeneration and blood–brain barrier breakdown in apolipoprotein E4 carriers with Alzheimer's disease. J. Cereb. Blood Flow Metab. *36*, 216–227.

Lu, T.M., Houghton, S., Magdeldin, T., Durán, J.G.B., Minotti, A.P., Snead, A., Sproul, A., Nguyen, D.-H.T., Xiang, J., Fine, H.A., et al. (2021). Pluripotent stem cell-derived epithelium misidentified as brain microvascular endothelium requires ETS factors to acquire vascular fate. Proc. Natl. Acad. Sci. USA *118*, e2016950118.

Montagne, A., Nation, D.A., Sagare, A.P., Barisano, G., Sweeney, M. D., Chakhoyan, A., Pachicano, M., Joe, E., Nelson, A.R., D'Orazio, L.M., et al. (2020). APOE4 leads to blood-brain barrier dysfunction predicting cognitive decline. Nature *581*, 71–76.

Montagne, A., Nikolakopoulou, A.M., Huuskonen, M.T., Sagare, A. P., Lawson, E.J., Lazic, D., Rege, S.V., Grond, A., Zuniga, E., Barnes, S.R., et al. (2021). APOE4 accelerates advanced-stage vascular and neurodegenerative disorder in old Alzheimer's mice via cyclophilin A independently of amyloid-β. Nat. Aging *1*, 506–520.

Nuriel, T., Peng, K.Y., Ashok, A., Dillman, A.A., Figueroa, H.Y., Apuzzo, J., Ambat, J., Levy, E., Cookson, M.R., Mathews, P.M., and Duff, K.E. (2017). The Endosomal–Lysosomal Pathway Is Dysregulated by APOE4 Expression in Vivo. Front. Neurosci. *11*, 702.

Patel, R., Cui, A., Bosco, P., Akcan, U., Richters, E., Delgado, P.B., Agalliu, D., and Sproul, A.A. (2024). Generation of hiPSC-derived brain microvascular endothelial cells using a combination of directed differentiation and transcriptional reprogramming strategies. Preprint at bioRxiv. https://doi.org/10.1101/2024.04.03.588012.

Patsch, C., Challet-Meylan, L., Thoma, E.C., Urich, E., Heckel, T., O'Sullivan, J.F., Grainger, S.J., Kapp, F.G., Sun, L., Christensen, K., et al. (2015). Generation of vascular endothelial and smooth muscle cells from human pluripotent stem cells. Nat. Cell Biol. *17*, 994–1003.

Porkoláb, G., Mészáros, M., Szecskó, A., Vigh, J.P., Walter, F.R., Figueiredo, R., Kálomista, I., Hoyk, Z., Vizsnyiczai, G., Gróf, I., et al. (2024). Synergistic induction of blood–brain barrier properties. Proc. Natl. Acad. Sci. USA *121*, e2316006121.

Prasad, H., and Rao, R. (2018). Amyloid clearance defect in ApoE4 astrocytes is reversed by epigenetic correction of endosomal pH. Proc. Natl. Acad. Sci. USA *115*, E6640–E6649.

Roudnicky, F., Zhang, J.D., Kim, B.K., Pandya, N.J., Lan, Y., Sach-Peltason, L., Ragelle, H., Strassburger, P., Gruener, S., Lazendic, M., et al. (2020). Inducers of the endothelial cell barrier identified through chemogenomic screening in genome-edited hPSC-endothelial cells. Proc. Natl. Acad. Sci. USA *117*, 19854–19865.

Sabbagh, M.F., Heng, J.S., Luo, C., Castanon, R.G., Nery, J.R., Rattner, A., Goff, L.A., Ecker, J.R., and Nathans, J. (2018). Transcriptional and epigenomic landscapes of CNS and non-CNS vascular endothelial cells. eLife *7*, e36187.

Simonneau, C., Duschmalé, M., Gavrilov, A., Brandenberg, N., Hoehnel, S., Ceroni, C., Lassalle, E., Kassianidou, E., Knoetgen, H., Niewoehner, J., and Villaseñor, R. (2021). Investigating receptor-mediated antibody transcytosis using blood-brain barrier organoid arrays. Fluids Barriers CNS *18*, 43.

Villaseñor, R., Lampe, J., Schwaninger, M., and Collin, L. (2019). Intracellular transport and regulation of transcytosis across the blood–brain barrier. Cell. Mol. Life Sci. *76*, 1081–1092.

Villaseñor, R., Schilling, M., Sundaresan, J., Lutz, Y., and Collin, L. (2017). Sorting Tubules Regulate Blood-Brain Barrier Transcytosis. Cell Rep. *21*, 3256–3270.

Wallander, M.L., Leibold, E.A., and Eisenstein, R.S. (2006). Molecular control of vertebrate iron homeostasis by iron regulatory proteins. Biochim. Biophys. Acta *1763*, 668–689.

Wevers, N.R., Kasi, D.G., Gray, T., Wilschut, K.J., Smith, B., van Vught, R., Shimizu, F., Sano, Y., Kanda, T., Marsh, G., et al. (2018). A perfused human blood-brain barrier on-a-chip for high-throughput assessment of barrier function and antibody transport. Fluids Barriers CNS *15*, 23.

Yamazaki, Y., Shinohara, M., Yamazaki, A., Ren, Y., Asmann, Y.W., Kanekiyo, T., and Bu, G. (2020). ApoE (Apolipoprotein E) in Brain Pericytes Regulates Endothelial Function in an Isoform-Dependent Manner by Modulating Basement Membrane Components. Arterioscler. Thromb. Vasc. Biol. *40*, 128–144.

Yang, A.C., Vest, R.T., Kern, F., Lee, D.P., Agam, M., Maat, C.A., Losada, P.M., Chen, M.B., Schaum, N., Khoury, N., et al. (2022). A human brain vascular atlas reveals diverse mediators of Alzheimer's risk. Nature *603*, 885–892.

Stem Cell Reports, Volume *20*

Supplemental Information

ApoE4 disrupts intracellular trafficking and iron homeostasis in a reproducible iPSC-based model of human brain endothelial cells

Luisa Bell, Shane Clerkin, Sila Rizalar, Antoine Rizkallah, Nadine Stokar-Regenscheit, Xandor M. Spijkers, Nienke R. Wevers, Claire Simonneau, Angélique Augustin, Barbara Höllbacher, Lia D'Abate, Joanna Ficek-Pascual, Kim Schneider, Desiree Von Tell, Thomas Maurissen, Chiara Zanini, Christelle Zundel, Sabrina Golling, Christine Becker, Alex Odermatt, Lynette C. Foo, Martina Pigoni, and Roberto Villaseñor

Supplementary Material

Supplementary Table 1. List of genes for benchmarking iCE-BECs towards endothelial transcriptomic signature. List adapted from Lu, Houghton, Magdeldin, Durán, Minotti, Snead, Sproul, Nguyen, Xiang, Fine, Rosenwaks, Studer, Rafii, Agalliu, Redmond, Lis [1].

High PC1 Loading								
**PECAM1**	FABP4	MYRIP	WDFY4	IL15RA	STK32B	SYNE3	TSPO	PGF
**CD93**	PALMD	CXCL8	FAM78A	CRIP2	MEOX2	EFEMP1	AHNAK2	NID1
**MMP1**	ZEB1	ITGB3	NPAS2	PDE2A	SPOCK1	WSCD1	NRG3	PARP12
**MMRN1**	MMRN2	MANCR	GBP4	IRAK3	RNASE1	EDN1	SLC9A3R2	PMP22
**CLEC14A**	GIMAP1	CXCR4	NRGN	ITGA10	HOXA10	PKD1L1	ALDH1A2	ST6GALNAC4
**ROBO4**	CNRIP1	DLL4	ADAMTSL1	GAB3	BACE2	PALD1	ULBP2	LINC01197
**SRGN**	ESM1	PDE4B	C2CD4B	ADCY4	TCTEX1D1	IL18R1	PAPSS2	TEK
**GIMAP4**	TM4SF18	FAM43A	MAPK11	LINC01358	TNS2	VIM	IFIT2	SP100
**GIMAP6**	GIMAP2	PDE7B	HLX	ACE	LMO2	PROCR	TNFRSF1B	PLXND1
**DIPK2B**	HHIP	RASGRP3	HOXD8	CD163L1	NEGR1	COX7A1	MTSS1	IFIT3
**ERG**	PLVAP	PIK3CG	GRAP	FRMD3	SERPINE1	IL3RA	VASH1	NRIP3
**VWF**	ADGRL4	CLDN11	NFIB	SEMA3G	SNED1	CLEC1A	SORBS2	HRH1
**LAMA4**	ABI3	LNCOG	CPNE5	LONRF3	RGCC	PCSK1	A2M	ECE1
**CLDN5**	TNFRSF14	NRN1	GRASP	CFAP54	HOXA11	NAV3	FAM124A	SH3TC2
**CAVIN2**	APOL3	IL33	ARHGEF28	MYCT1	TNFSF4	INKA1	KLF9	DYRK3
**STAB1**	LAPTM5	IFI44	MALL	THBD	FAM241A	CCL2	NRG1	SYNM
**GIMAP8**	PCDH12	KLF2	LY96	CARD8-AS1	CNTNAP3B	HIC1	SSTR1	LTBP2
**EMCN**	ECSCR	CARD16	GJA4	CHCHD2	LHX6	RAMP2	TLE2	DPYD
**CD34**	ENG	MGP	DKK1	CXCL1	AFAP1L1	NT5E	MEDAG	CDKN2C
**ICAM2**	SOX17	INSYN2B	STEAP1B	SHANK3	MPP4	TMEM156	MIR155HG	HOXD1
**BCL6B**	TNFSF10	SGIP1	THSD1	FLT1	MLIP	HHIP-AS1	SLC17A9	SPESP1
**BMX**	GNG11	FERMT3	LIX1L	NLRC5	SENCR	TM6SF1	PLA2G4C	STEAP1
**FAM124B**	CALCRL	HCLS1	CDYL2	CASP4	GNGT2	MIR137HG	GIPC3	CLDN14
**LYVE1**	TM4SF1	TMEM204	FLT4	NOS3	ZFYVE28	NPR1	IL7R	ADGRA2
**ACVRL1**	S1PR1	BMP6	GGT5	EGFL7	NFIC	HOXD9	SLC43A1	EBF1
**ESAM**	LYL1	SPAAR	C22orf34	SAMD9	EMP1	DDR2	PCDH10	ITGA11
**TIE1**	BGN	GMFG	NFIA	VAMP5	PARVB	STX11	APOLD1	MSRB3
**APLN**	TMEM173	LDB2	PREX1	PLXNA4	NTSR1	PTPRE	SYT11	CCNA1
**PPP1R16B**	CLEC2B	NOTCH4	HOXA9	MILR1	IFI27	SH3RF3	ZNF469	RFTN2
**SHE**	ANGPT2	TMEM255B	MLKL	CPT1A	HAGLR	MEF2C	TLL1	ARHGEF6
**ANPEP**	SH2D3C	CDH5	COL8A1	CAV1	ANKRD55	DMTN	LINC01094	MAMLD1
**ARHGEF15**	DOCK10	RASIP1	SEMA6B	KANK3	SH2B3	STC1	FOXF1	TBXA2R
**HHEX**	LINC01013	CHST1	SYNPO	ZNF366	CD109	MCTP1	ST8SIA4	IL4I1
**TAL1**	PTPRB	SCARF1	TFEC	LRRC70	CEACAM21	LGALS9	UAP1L1	GAPLINC
**PTX3**	KLHL6	CYTL1	COL13A1	SLFN11	ZEB2	GBP1	ENTPD1	HSD17B2
**RHOJ**	FAM107A	FGD5	PLCL1	SERPIND1	NTN4	LRRC8C	FAM155A	RAPGEF5
**LINC01235**	TLR4	FOXC2	CASP1	SAMSN1	EVI2B	MIR217HG	USHBP1	IL6R
**SOX18**	MEG3	TMEM273	HTR2B	KCTD12	PREX2	ARHGAP20	ARHGAP22	OAF
**MFNG**	EVA1C	P4HA3	ERAP2	ANXA2R	LINC00520	KIAA1549L	ITGA5	ARHGAP24
**GPR4**	SELP	FLI1	CCRL2	VEPH1	TNFRSF11A	GBP2	RFLNB	NOX4
**ADGRF5**	TNFAIP8L3	SH3TC1	TOX2	LINC02454	NFIA-AS2	MT2A	LPAR6	
**PCAT19**	NOVA2	LGALS1	LINC01116	CTSS	ABLIM3	UBA7	CUBN	
**GIMAP7**	KDR	TDRD10	THBS1	LOX		TGFBR2	ARAP3	
**IL1RL1**	CARD6	RAC2	PLSCR4	TBX18				

					KCNN3<br>DYSF	F2RL2	CSGALNACT1	
**Low PC1 loading**								
**CLDN6**<br>**GPC3**<br>**LIN28A**<br>**PTN**<br>**DSG2**<br>**IGDCC3**<br>**CRABP2**<br>**DSC2**<br>**AP1M2**<br>**EPCAM**<br>**DMKN**<br>**RBM47**<br>**ACTC1**<br>**QPRT**<br>**SPP1**<br>**GPC6**<br>**SLC1A3**<br>**TRIM71**<br>**LIN28B**<br>**MSX2**<br>**ENPEP**<br>**COL1A1**	SERPING1<br>ZFP42<br>MMP9<br>NKAIN4<br>WFDC2<br>LCP1<br>ROR2<br>ID4<br>CRYBG2<br>HPGD<br>WDR86<br>SOX9<br>LYPD6B<br>DSP<br>APOE<br>DPPA4<br>EFS<br>H2AFY2<br>CD24<br>H19<br>FBLN1<br>EMB	NPPB<br>GPC4<br>MARVELD3<br>TINCR<br>TNC<br>SYTL1<br>KRT8<br>PDPN<br>NLGN4X<br>IGFBP5<br>SHANK2<br>GPR87<br>MFAP5<br>ARSI<br>TRIM55<br>LRRN4<br>RUBCNL<br>PKIB<br>PRSS16<br>GYG2<br>PURPL<br>CHPF	ACSS3<br>ADAMTS19<br>CTSV<br>RARRES2<br>SPINT2<br>CDH8<br>TFAP2A<br>ERVH48-1<br>SLC27A6<br>IGF2<br>MLPH<br>MYO5B<br>WDR86-AS1<br>TENM3-AS1<br>NSUN7<br>MEIS3<br>MYL7<br>PARP8<br>HAPLN1<br>WNT5B<br>OVOL2<br>CCDC144NL-AS1	NEO1<br>PCDH11X<br>SORL1<br>CPVL<br>MXRA8<br>C4orf19<br>LINC02381<br>HAS2<br>PROM1<br>LEF1<br>CADM1<br>GPRC5C<br>SALL4<br>FAM169A<br>MOB3B<br>CCDC8<br>UNC5C<br>SH2D4A<br>GRIP1<br>RIPOR2<br>DACT1<br>ALPK2	SEMA5A<br>RAMP1<br>GLI2<br>NRK<br>CAMK1G<br>CDX2<br>LUM<br>ALPL<br>PCDHA12<br>JPH2<br>IGFBP3<br>SOX11<br>PCSK1N<br>PKP2<br>TMEM92<br>XKR4<br>PPP2R2B<br>LINC01224<br>CNN1<br>SLC4A4<br>TEAD3<br>TRMT9B	DSC3<br>GALNT17<br>MPPED2<br>IGF2-AS<br>ADGRV1<br>CA2<br>DIO3OS<br>TNFRSF19<br>GAS7<br>GALNT3<br>ERP27<br>KRT19<br>EPS8L2<br>NR6A1<br>NTRK2<br>WWC1<br>PLBD1<br>PDGFRB<br>LMOD1<br>RGS16<br>RIMS2<br>SSC4D	PRKCZ<br>FERMT1<br>ST6GAL2<br>OXTR<br>NSG1<br>PCAT14<br>LRRTM4<br>DCDC2<br>STXBP6<br>CA3<br>SERPINF1<br>PLD5<br>IRS1<br>SRGAP3<br>CHMP4C<br>LSR<br>AFAP1L2<br>ADAMTS2<br>CLDN10<br>BOC<br>LINGO1<br>SCG3	COL4A6<br>COL1A2<br>FUT9<br>ADCY10<br>NPFFR2<br>L1CAM<br>PKDCC<br>TBX3<br>FLRT3<br>LINC00648<br>IGDCC4<br>DLK1<br>CORO2A<br>EDNRA<br>PLPPR3<br>PATJ<br>SALL1

Extraction of the top 500 genes with strongest positive and 500 genes with strongest negative contribution to PC1 from sheet "Fig.1C PC1 Loading Genes" from dataset S02 of the meta-analysis from Lu, Houghton, Magdeldin, Durán, Minotti, Snead, Sproul, Nguyen, Xiang, Fine, Rosenwaks, Studer, Rafii, Agalliu, Redmond, Lis [1]. Subset of extracted genes that were expressed in our cells (392 for high PC1 loading, 193 for low PC1 loading) are listed below. Genes with high PC1 loading are associated with endothelial signature, while low PC1 loading are related to epithelial identity, see Figure 1D.

Supplementary Table 2. The top 100 most variable genes contributing to each Principal Component in the analysis shown in Figure 2H. iCE-BECs generated from three different iPS lines (SFC086_03_03 line, Bioni037A, Bioni10C) and across passages were compared.

The top 100 most variable genes contributing to each Principal Component in the analysis shown in Figure 2H. iCE-BECs generated from three different iPS lines (SFC086_03_03 line, Bioni037A, Bioni10C) and across passages were compared.

Supplementary Materials and Methods

Generation details for ApoE4 lines

For the Bioni037 line, the parental homozygous ApoE3 line was generated from the fibroblasts of a healthy individual with homozygous ApoE3 genetic variant .The isogenic ApoE4 homozygous line was derived from the parental line by editing of the ApoE allele from T/T to C/C at rs429358, which changes aa from Cys112 to Arg112; the genetic variant of base position described by rs7412 in both parent and subclone is C/C, which is an Arg. Together, these define the ApoE4/E4 genetic variant in this subclone. Editing was confirmed and cells were characterized (sequencing, expression of pluripotency markers, morphology) by the European Bank for induced pluripotent Stem Cells (ebisc.org). The parental Alstem line iPS16 was reprogrammed from one single iPSC clone of human bone marrow CD34-positive mononuclear cells. The isogenic control line carrying ApoE3 (iPS26, Alstem) was derived from the parental Alstem line by changing Arg112 to Cys112 in the ApoE allele. Editing was confirmed and cells were characterized (sequencing, expression of pluripotency markers, morphology) by Alstem (alstembio.com).

Single cell RNAseq sequencing and analysis

300,000 cells differentiated with the iEC, iEC-Rep and iCE-BEC protocol (see Figure 1A) were plated in 6 well plates at day 11 and grown at confluence to day 14. On day 14, cells were detached and resuspended into a single cell suspension concentrated $1x10^6$ cells per mL. Cells were then processed following the Chromium Next GEM Single Cell 3' v3.1 protocol for GEM generation and barcoding followed by gene expression library construction according to the manufacturer's instructions. Targeted cell recovery of 10'000 per sample was performed. Dual indexed libraries were sequenced on the Novaseq 6000 with a target sequencing depth of 50'000 single reads per cell. Raw reads were processed with Cell Ranger software (version 7.1.0) and aligned to hg38 reference transcriptome. Count matrices generated by Cell Ranger were corrected for ambient RNA using CellBender 0.2.0 with the expected cells parsed from the Cell Ranger web summary. The number of total droplets to

include was set to 25000, the false positive rate set to 0.01 and the algorithm was trained for 150 epochs. CellBender corrected count matrices were then concatenated and further processed with scanpy (v1.9.3). Barcodes were filtered for observations that CellBender had assigned a latent cell probability greater than 0.5. Furthermore, we excluded low quality cells and potential duplicates by retaining cells with percentage of mitochondrial counts < 5% and with a number of genes between 200 and 2500. Genes detected in less than 30 cells were removed from downstream analysis. Counts were normalized by library size and log1p transformed before determining highly variable genes and regressing out the effect of library size and percentage of mitochondrial counts. Top 10 principal components were used as input for the neighborhood graph which in turn was used to create the UMAP representation. We utilized the meta-analysis published by Liu and colleagues [1] to generate gene sets associated with an endothelial transcriptomic signature (high PC1 loading) or an epithelial transcriptomic identity (low PC1 loading). In detail, we extracted the top 500 genes with strongest positive and 500 genes with strongest negative contribution to PC1 from sheet "Fig.2C PC1 Loading Genes" from dataset S02 provided as supporting information to their publication (see Supplementary Table 1). We subset their genes to those expressed in our data (392 for high PC1 loading, 193 for low PC1 loading) and visualized the score for the two gene sets through the Seurat (v5.0.1) function AddModuleScore.

We used the scanpy score_genes function to compute per cell scores of the brain endothelial gene set [*CLDN5, MFSD2A, SLC16A1, SLC3A2, SLC38A5, SLC7A5, SLC2A1*]. We then focused on endothelial cells (defined as transcript expression > 0 for at least one of the general endothelial markers [*KDR, VWF, PECAM1, ENG, CDH5, FLT4, FCGR*] and investigated the brain endothelial scores grouped by differentiation protocol.

Bulk RNA sequencing

300,000 cells differentiated with the iEC, iEC-Rep or iCE-BEC protocol (see Figure 1A) were plated at day 11 and grown at confluence to day 14. Differentiation protocols were tested in triplicate, yielding a total of 9 samples (three independent differentiations per condition) for

sequencing. On day 14, cells were collected, and pellets were snap frozen. RNA was extracted and preparation of Illumina stranded TruSeq RNA libraries, including poly(A) enrichment, was performed (2*100 bp paired-end reads). Samples were sequenced on the Illumina NovaSeq and NextSeq by Microsynth. Base calling was conducted using the BCL to FASTQ file converter bcl2fastq2 version 2.20.0 (Illumina). Quality assessment of FASTQ files was performed with FastQC version 0.12.1 (Andrews et al. 2010). Paired-end reads were aligned to the human genome (build "hg38") using the STAR read aligner version 2.7.11b with default mapping parameters (Dobin et al. 2013). Alignment metrics were determined using Picard version 3.1.1 (Broad Institute). Quality of read sequences and alignments was assessed with MultiQC version 1.21 [2]. The number of reads mapped to all RefSeq transcript variants of a gene were combined into a single count value (i.e., read count) assuming a reverse-stranded library, using featureCounts version 2.0.6 [3]. Read counts for 43,294 RefSeq transcripts were generated for all 9 samples. All samples passed quality control checks. Read count normalization, principal component analysis (PCA) and gene ontology (GO) term enrichment were subsequently conducted in R (version 4.3.0). Read count normalization was performed using the edgeR package (version 4.0.5; [4]). Library size and composition were adjusted for using Trimmed mean of M (TMM)-normalized CPM values. Only samples with >=1 CPM and >10 read counts in at least 3 samples were retained. Following gene filtering, a total of 13,536 of 43,294 transcripts were identified as expressed (31%). The PCA was computed based on the top 500 most variable expressed genes using TMM-normalized log2(CPM+1) values. Eigenvalues were extracted and the cumulative percentage of each principal component’s (PC) variance explained. PC1 contributed most to sample variance (82.65%), with PC2-4 individually contributing <7.3% each. GO term enrichment was performed on the top 100 loading genes for PC1 using the clusterProfiler package in R (version 4.10.0). Multiple testing correction was performed using the Benjamini-Hochberg method.

Bulk RNA sequencing across lines and passages

iCE-BECs were differentiated using the protocol aforehand described (Figure 1A) starting from 3 parental iPSC lines (hiPS_SFC086_03_03, BIONi010-C13 and Bioni037-A). At least 300,000 cells were collected on day 14 (“Passage 0”). To evaluate the effect of cell passages on cell identity, hiPS_SFC086_03_03 at day 14 were frozen, thawed and subsequently passaged for 3 consecutive times (“Passage 1-3”). The preprocessing was performed in the same way as described above, for the 8 samples. Following gene filtering, a total of 12,887 of 43,294 transcripts were identified as expressed (30%). The PCA was computed based on the top 500 most variable expressed genes using TMM-normalized log2(CPM+1) values. Eigenvalues were extracted and the cumulative percentage of each principal component’s (PC) variance explained. PC1 contributed most to sample variance (67.65%), with PC2-4 individually contributing <15% each. Loadings for all genes were extracted, and the top 100 genes based on absolute loading scores contributing to PC1 and PC2 were examined for the presence of general and brain endothelial markers.

FITC-dextran Permeability in Transwell

50,000 cells were plated at day 11 on the insert of transwell chambers (734-4072, Avantor). Media was changed at day 12 and FITC-dextran permeability experiments were run at day 14 in culture. Briefly, the inserts were moved to a new Receiver Tray supplemented with 600 µL of fresh media. A FITC-dextran (3.3 kDa, D3305; 40 kDa, D1845; 70 kDa D1822, all purchased from ThermoFisher) dilution of 50 µg/ml was added in the insert compartment and plates were incubated for 30 minutes at 37 °C. After incubation, 100 µL of the media from each Receiver Tray were transferred to wells of a black 96-well opaque plate (PBK96G-1.5-F, MatTek USA) for fluorescence measurement. Fluorescence was read at 485 nm and 535 nm excitation and emission, respectively and apparent permeability (P_{app}) calculated using the formula:

$$P\ app = (dQ/dt) \times 1/AC$$

where dQ/dt is change in concentration / change in time, A is the growth area in the insert and C is the initial concentration in the insert chamber.

Histology of transwell

iCE-BECs were seeded on a transwell (734-4072, Avantor) as described above and fixed at day 14 in 4% PFA for

30 min, subsequently washed with PBS for three times. Cells including the transwell mesh were embedded in 2% Agarose (V3121, Promega), dehydrated overnight (TissueTek VIP5, Sakura) and ultimately embedded vertically in paraffin using an embedding console (Tissue-Tek® TEC™5, Sakura). Sagittal microtome sections at 4 um were prepared on Superforst Plus glass slides (J1800AMNZ, ThermoFisher) and an automated Haematoxylin & Eosin staining was performed (Ventana HE600, Roche). Slides were imaged with a whole slide scanner at 40 × (Hamamatsu, NanoZoomer S360, standard HE).

Transendothelial electrical resistance (TEER) assessment

iCE-BECs and iECs were seeded into transwells (3470, Corning) on day 11 at a density of 120,000 cells per 6.5 mm insert and maintained in their respective maintenance media to form monolayers. On day 14, inserts were transferred to the wells of a cellZscope+ device (nanoAnalytics) in 1:5 diluted media (1:5 BBB Identity Maintenance media : basal Vasculife without media supplements for iCE-BECs, or 1:5 Vasculife Maintenance media : basal Vasculife without media supplements for iECs). Diluted media was used to reduce the concentration of VEGF which is known to alter cell permeability. Impedance was continuously measured every 1 h, and TEER and capacitance (Ccl) were automatically calculated as readout parameters. TEER values for iCE-BECs at 18 hours were normalized to the iEC condition and data was presented as a fold change comparison.

Permeability assessment in microphysiological system

Barrier function of iCE-BECs and HBMVECs cultured in a microphysiological system were compared by assessing leakage of fluorescent dextran dye. iCE-BECs were generated according to the protocol described above and seeded in an OrganoPlate® 2-lane (9605-400-B, MIMETAS) according to the manufacturer's protocol [5]. Briefly, one day before the cell

seeding, ECM (3447-020-01, Cultrex 3D Collagen I, R&D Systems in 1M Hepes, Gibco and 37 mg/mL $NaHCO_3$) was prepared. Per tube, 35.000 cells were seeded and supplied with complete BBB identity maintenance media. HBMVEC cultures (OrganoReady® BBB HBMEC, MI-OR-HB-01, MIMETAS) were cultured according to the manufacturer's instructions. iCE-BECs and HBMVECs formed tubular structures against the ECM gel upon medium perfusion by placing the OrganoPlates on the OrganoFlow® perfusion rocker (MI-OFPR-S, MIMETAS). After 72 hours, media was replaced with 1:5 diluted media (1:5 BBB Identity Maintenance media : basal Vasculife without media supplements) for iCE-BECs. At day 4 of culture, 65-85 kDa TRITC dextran (0.25 mg/mL, Sigma-Aldrich, T1162) was added to the lumen of iCE-BECs and HBMVEC cultures. Images were taken every 2 minutes for a duration of 12 minutes, and at t=40 min after dye addition using an ImageXpress XLS Micro HCI System (Molecular Devices).

To test the barrier function of iCE-BECs in response to VEGF and across different lines (SFC086_03_03, BIONi010-C13, Alstem iPS26, and BIONi037-A) in a microphysiological system, cells were seeded in an OrganoPlate® 2-lane (9605-400-B, MIMETAS) as described above. After 72 h, media was replaced with 1:5 diluted media (1:5 BBB Identity Maintenance media : basal Vasculife without media supplements for iCE-BECs) for basal conditions or additionally supplemented with 0.2 µg/mL VEGF165 (293-VE-010, R&D). After 24 h incubation, differently sized FITC-dextrans (3.3 kDa, D3305; 40 kDa, D1845; 70 kDa D1822, all purchased from ThermoFisher) were applied at 10 µg/mL and immediately imaged using Opera Phenix High Content Imaging System (PerkinElmer) at 5× magnification, every five minutes for two hours. The ratio between mean intensity of FITC-dextran in the cell channel and the gel channel was calculated for each perfusable tube at each time point.
To calculate the apparent permeability, the slope of the linear regression was multiplied by the volume of the gel (0.0004136 cm^3) and subsequently divided by the surface area (0.01218153 cm^2) adapted from [6] according to the manufacture's protocol.

Brainshuttle™ transcytosis assessment in microphysiological system

To assess transcytosis of Brainshuttle™ molecules across iCE-BECs, cells were seeded in an OrganoPlate® 2-lane (9605-400-B, MIMETAS) as described above. After 72 h, media was replaced with 1:5 diluted media (1:5 BBB Identity Maintenance media : basal Vasculife without media supplements for iCE-BECs). After 24 h, fluorescently labeled Brainshuttle™ antibody or a non-targeting IgG control were applied at 200 nM, and immediately imaged using Opera Phenix High Content Imaging System (PerkinElmer) at 5× magnification, every 15 min for twelve hours. The ratio between mean intensity in the donor cell channel and the gel channel was calculated for each perfusable tube at each time point for both Brainshuttle™ and non-targeting IgG. To assess total transcytosis of Brainshuttle™, the slope of the linear regression was calculated within the time interval 0 to 12 h. Before each experiment, channels with matrix overflow or incomplete filling of matrix channel were excluded from the analysis.

Electron microscopy

To assess the ultrastructural morphology of endosomes, cell pellets of iCE-BECs were generated and fixed in 2.5% Glutaraldehyde (pH 7.4) overnight. After lipid fixation with 1% Osmium-Tetroxid for 1 h, samples were dehydrated with ascending ethanols and finally infiltrated with resin by incubating two times with Propylenoxid for 15 min each and Propylenoxid/epon (1:1 ratio) overnight at RT. Samples were transferred to epon blocks and polymerized at 60°C for 60 hours. Ultrathin sections (98 nm) were prepared on 200 mesh copper grids (EMS, Fort Washington, PA, USA) and afterwards contrasted with lead citrate and uranyl acetate. The sections were examined with a Philips CM10 transmission electron microscope equipped with a charge-coupled-device camera (Ultrascan 1000; Gatan) at an acceleration voltage of 80 kV.

Quantitative PCR

mRNA was extracted from iCE-BECs cell cultures with APOE genetic variants using Total RNA Miniprep Kit (T2010, Monarch) following the manufacturer's instructions. Complementary DNA was synthesized using iScript cDNA Synthesis kit (1708890, BioRad). Quantitative real-time PCR analysis was performed with Lightcycler 480 SYBR Green I Master mix (04887352001, Roche; LightCycler® 96 System, Roche). Ready-to-use primers from Origene for ApoE (HP200028), TFR1 (HP206788), FTH (HP205786), FTL (HP200131), FPN (HP210988), DMT-1 (HP200584), and GAPDH (HP205798) were used. Primer efficiency was determined by titration of cDNA from iCE-BECs ApoE3; all tested primers had an efficiency between 80%-110%. The cycle threshold (Ct) values were used for all experiments and were first normalized to endogenous control (GAPDH) levels by calculating the ΔCt for each sample. Values were then analyzed relative to control, to generate a ΔΔCt value. Fold change was obtained using the equation, expression fold change=$2^{-\Delta\Delta Ct}$.

Immunoblot

Cells were lysed with RIPA buffer (89900, LifeTech) and incubated for 30 min on a rotary shaker at 4°C. Cells were then centrifuged at 12,000 ×g at 4°C for 12 minutes, and the supernatant were retained for protein quantification using the Pierce bicinchoninic acid assay method (23225, ThermoFisher). 5-20 µg of protein was typically loaded per sample in NuPAGE LDS Sample Buffer (4x) (NP0007, Thermo Fisher) containing NuPAGE Sample Reducing Agent (NP0004, Thermo Fisher) as per manufacturer’s instructions. Samples were denatured at 95°C for 5 min. Immunoblots were resolved using 4-15% Mini-Protean TGX Stain-Free gels (4568085, Bio-Rad) and transferred on 0.2 µm nitrocellulose membranes (1704159, Bio-Rad) using a Trans-Blot Turbo Transfer System, (BioRad). Membranes were subsequently blocked with 5% Milk in Tris-buffered saline with 0.1% Tween® 20 Detergent (TBS-T) for 1h followed by primary antibody incubation in 5% Bovine Serum Albumin (BSA) in TBS-T overnight (TfR1, 13-6800, ThermoFisher, 1:250; ApoE, Ab947, abcam, 1:500; FTH, 3998S, Cell Signaling Technology, 1:250; DMT-1, 20507-1-AP, ThermoFisher, 1:500; GAPDH-HRP, 8884, 1:25000, Cell Signaling Technology). After three times washing in TBS-

T, appropriate HRP-conjugated secondary antibodies in 5% BSA in TBS-T were applied (donkey-anti-rabbit-HRP, A16035, ThermoFisher, 1:2000; donkey-anti-goat-HRP, A16005, ThermoFisher, 1:2000; donkey-anti-mouse-HRP, A32788, ThermoFisher, 1:2000) for 1h at RT. Immunoblots were washed three times in TBS-T for five minutes each, and protein bands were then visualised using SuperSignal West Pico Plus Chemiluminescent Substrate (34580, ThermoFisher). Image acquisition was performed using the ChemiDoc MP (BioRad). Densitometric quantification of immunoblot bands was performed by measuring the optical density of the signal for each protein of interest and normalizing it to the optical density of the corresponding GAPDH band, followed by further normalization to the ApoE3 signal (ImageJ 1.54).

Whole cell proteomics

To benchmark iCE-BECs, primary brain endothelial cells (HBMVEC, n = 6 from three different batches), immortalized endothelial cells (HCMEC/D3, n = 3 different passages), and induced ECM-supported brain endothelial cells (iCE-BECs, n = 4 differentiations) were analyzed. HCMEC/D3 (SCC066, Merck) cells were cultured in EGM-2 Endothelial Cell Growth Medium-2 BulletKit (CC-3162, Lonza) for three sequential passages and collected when at confluence. Three independent batches of HBMVECs were purchased from AngioProteomie (cAP-0002, AngioProteomie) and cultured in EGM-2 Endothelial Cell Growth Medium-2 BulletKit (CC-3162, Lonza) in flasks coated with quick coating solution (cAP-01, Angioproteomie). Each cell batch was cultured for two subsequent passages and collected when at confluence. iCE-BECs were differentiated as previously described and collected on day 14 in culture. For whole proteomics analysis, culture media were removed and cells were washed with phosphate buffered saline (PBS). Cells were detached using trypsin 0.25%-EDTA (ThermoFisher, 25200056) for HCMEC/D3 or TrypLE™ (12563011, ThermoFisher) for HBMVECs and iCE-BECs, resuspended in culture medium and centrifuged at 180 g at 4°C for 5 min. Cell pellets were then washed with cold PBS and centrifuged. This washing step

was repeated again once before snap freezing the cell pellets on dry ice for about 15 min. Cell pellets were then stored at -80°C.

Samples were reduced, alkylated, digested with trypsin and peptides purified using the PreOmics iST kit according to the supplier's specifications. Peptide samples were resuspended in 2% (v/v) acetonitrile and 0.5% (v/v) formic acid solution and 1 ug of peptides were analyzed by liquid chromatography (nano capillary system, EASY-nLC™ 1200 system, Thermo Scientific) on a C18 reverse-phase nano-high-performance liquid chromatography column connected to a mass spectrometer (Orbitrap Exploris™ 480, Thermo Scientific) via electrospray ionization. The DIA method consisted of one full range MS1 from 340 to 1210 m/z at 120k resolution, with a custom AGC target and 20ms max injection time. Then 28 DIA segments were acquired at 15k resolution with a standard AGC target and 20 ms max injection time. HCD fragmentation was set to normalized collision energy optimized for each segment. The spectra were recorded in profile mode. The default charge state for the MS2 was set to 3. Raw files have been processed with Spectronaut 18, with experiment settings based on BGS Default SNE for a DIA library free search, using global imputation to deal with missing values. Default settings included peptide and protein level false discovery rate control at 1 %. Measurements were normalized separately using local regression normalization. The mass spectrometric data were analyzed using Pulsar search engine as implemented in Spectronaut software, the false discovery rate on peptide and protein level was set to 1 %. A human UniProt fasta database (Homo Sapiens, 2022 07 01) was used for the search engine, allowing for 2 missed cleavages and variable modifications (N term acetylation and methionine oxidation).

Distributions of both raw and normalized data at the protein level were assessed to evaluate sample consistency. Principal Component Analysis (PCA) was conducted to reveal the overall data structure and to identify potential experimental artifacts. Outlier detection was implemented using the Mahalanobis distance, calculated from the first three principal components. No outliers were removed. The differential abundance analysis was performed to identify proteins with significant changes in expression levels between the different

conditions. For this statistical analysis, we utilized the R package “limma”. Unlike standard t-tests, which compare proteins individually, the “limma”; approach accounts for the overall variance observed across all proteins. This typically results in adjusted P-values, especially at the tails of the distribution, and is particularly well-suited for studies with small sample sizes.

Proliferation assay

To assess the proliferation rate between iPSC with ApoE3 and ApoE4 genetic variant, iPSCs were seeded on geltrex (A1413301, ThermoFisher) coated 96-well plate (PBK96G-1.5-F, MatTek USA, 12.000 cells/well) in ROCK inhibitor Y-27632 containing (SCM075, EMD Millipore) mTeSR Plus media (100-0276, Stemcell). Media was changed every 24 hours. Confluency of iPSCs was assessed every 24h for 8 consecutive days using Live/dead staining cell imaging kit (R37601, ThermoFisher) according to manufacturer's protocol. Live imaging of whole wells was performed with Opera Phenix High Content Imaging System (PerkinElmer) at 20× with three wells per condition and time point. Live cell area was measured by absolute threshold and expressed as percentage of total well area (confluency). Non-linear regression (logistic growth) was performed (GraphPad Prism 10.2.2.).

Flow cytometry

iCE-BECs with ApoE3 or ApoE4 genetic variant (80.000/well in 24-well plate) were treated with fluorescently labeled transferrin (T13342, ThermoFisher) at 25 µg/mL for 20 min at 37°C or left untreated, followed by incubation of CD31-AF700 (NB600-562AF700, Novus Biological, 1:100) for 30 min at 4°C. For testing marker expression in cells generated with the iEC or iCE-BEC protocol (100.000/well in a 24-well plate), cells were collected and incubated with primary antibodies in FACS buffer (PBS + 0.5% BSA + 2 mM EDTA) for 30 minutes at 4°C (see Table 3), The LIVE/DEAD™ Fixable Aqua Dead Cell Stain Kit (L34957, ThermoFisher) was used to determine cell viability for FACS analysis. For intracellular staining, cells were

stained with cell viability dye, fixed in 4% PFA for 30 min at RT, and then incubated with anti-vWF or anti-ERG in FACS buffer for 30 min at 4°C (see Table 3). Cells were subsequently washed with FACS buffer, and fluorescence was immediately acquired using a Flow Cytometer (CytoFLEX LX, CytExpert software, Beckman Coulter) in duplicates. Unstained negative controls were used to adjust gain for FITC, PE, or AF700, respectively. Per sample, at least 10,000 live cells (singlets) were acquired. Data was exported as in fcs format and analyzed in FlowJo v10.0.0 (BD Biosciences). After initial cleanup to remove debris, doublets and to select for live cells, we employed a two-step gating strategy for PECAM1-positive cells. The first gate identified PECAM1-positive cells using unstained controls as a baseline. Subsequently, within that positive population, a second more stringent gate was set at a considerably higher fluorescence level of PECAM1 in a way that isolate the iCE-BEC population, aiming for close to 100% positivity within this homogeneous group. Both gates, termed low and high PECAM1-expressing cells were consistently applied to all iEC and iCE-BEC samples. Frequencies of total PECAM1-positive cells of live cells and high PECAM1-positive expression cells of PECAM1-positive cells were extracted. For comparison of marker expressions in cells generated with the iEC or iCE-BEC protocol, cells were gated based on PECAM1-positive signal. Median fluorescence intensities (MFI) of VE-Cadherin, vWF, ERG, Claudin-5, GLUT1 and LDL-R were extracted in PECAM1-positive population and divided by the MFI of the unstained control for each condition and differentiation.

Live imaging of FerroOrange to assess labile iron pool

Intracellular labile iron was measured using BioTracker™ FerroOrange Live Cell Dye, a fluorescent probe that specifically detects labile iron (II) ions (Fe^{2+}) only. Briefly, cells were washed and incubated with 1 µM BioTracker™ FerroOrange Live Cell Dye (SCT210, Sigma) and cell marker (CytoTrace Green, 22017, AAT Bioquest) in HBSS at 37°C for 30min. Live cell imaging was performed using Opera Phenix High Content Imaging System (PerkinElmer) with a 63×/1.2 NA objective, 4 wells per condition, 10 fields per well, 8 z-stacks with 1 µm step size. Sum intensity of FerroOrange was normalized to the total area of the image

covered by cells (calculated using an absolute intensity threshold of CytoTrace Green). As controls, to artificially increase LIP, cells were treated with an iron donor, ferrous ammonium sulfate at 100 µM (FAS, 203505, Sigma), while treatment with iron chelator PIH at 10 µM (ab145871) was used to deplete LIP before FerroOrange Live cell dye was applied.

Calcein-AM assay to assess labile iron pool

Intracellular labile iron was measured using the metal-sensitive probe Calcein acetoxymethyl ester (Calcein-AM), as previously described [7]. This is a non-fluorescent dye that becomes fluorescent after enzymatic modification once it permeates the cell membrane [7-9]. This fluorophore binds iron stoichiometrically, which quenches its green fluorescence. In short, cells were washed, incubated with 0.5 µM of Calcein-AM (C1430, ThermoFisher) in HBSS and whole cell marker (HCS CellMask™ Stain Deep Red, H32721, ThermoFisher) for 20 min at 37°C. Iron chelator PIH at 10 µM (ab145871, abcam) was applied to half of the wells for 10 min. Cellular calcein fluorescence was measured in live cells using Opera Phenix High Content Imaging System (PerkinElmer) at 20×, 5 wells per condition, 16 fields per well, 3 z-stacks à 1 µm. The ratio between the sum intensity of calcein within the cell area (absolute threshold of CellMask) in untreated cells and iron chelator-treated cells was calculated, reflecting the amount of the labile iron pool. Fold changes were calculated by normalization to ApoE3 per each experiment.

Supplementary Table 3. Primary antibodies used for immunostainings

Target	Concentration	Catalog No.	Vendor
Early Endosome Antigen 1 (EEA1)	1:200	3288S	Cell signaling
Transferrin Receptor 1 (TfR1)	1:200	13-6800	ThermoFisher
VE-Cadherin	1:200	2500S	Cell signaling
VE-Cadherin (microphysiological system)	1:1000	ab33168	abcam

PECAM1	1:200	NB600-562	Novus Biologicals
PECAM1 (microphysiological system)	1:20	M0823	Dako
Ferritin Heavy chain (FTH)	1:200	ab65080	Abcam
Zonula occludens-1 (ZO-1)	1:200	33-9100	ThermoFisher
Divalent metal transporter 1 (DMT-1)	1:200	ab55735	Abcam
Claudin-5 (required methanol fixation)	1:200	352588	ThermoFisher
SLC2A1 (GLUT-1)	1:100	07-1401-AF1488	SigmaAldrich
ABCB1 (PgP)	1:50	919403	BioLegend
ABCC1 (MRP1)	1:100	PA5-88082	ThermoFisher
ABCA1 (CERP)	1:100	AF7207	R&D Systems

Supplementary Table 4. Secondary antibodies and fluorescent probes for immunostainings

**Class**	**Concentration**	**Origin specie**	**Catalog No**	**Vendor**
anti-rabbit-Alexa488	1:200	Donkey	711-545-152	Jackson ImmunoResearch
anti-mouse-Alexa488	1:200	Donkey	715-545-150	Jackson ImmunoResearch
anti-rabbit-Alexa647	1:200	Donkey	711-605-152	Jackson ImmunoResearch
anti-mouse-Alexa647	1:200	Donkey	715-605-150	Jackson ImmunoResearch
anti-sheep-Alexa647	1:200	Donkey	713-605-147	Jackson ImmunoResearch
Phalloidin-647	1:400	n.a.	65906	Sigma

Supplementary Table 5. Primary antibodies used for flow cytometry

Target	Conjugate	Concentration	Catalog No.	Vendor
Von Willebrand Factor	AF488	1:100	ab195028	abcam
ERG	AF488	1:50	ab196374	abcam
GLUT1	AF488	1:50	ab195359	abcam
PECAM1	AF700	1:100	NB600-562AF700	Novus Biological
PECAM1	PE	1:100	NB600-562PE	Novus Biological
Claudin-5	AF488	1:100	352588	ThermoFisher
VE-Cadherin	PE	1:50	AF938	R&D Systems
LDL Receptor	PE	1:50	LS-C130473	LSBio

Transferrin kinetics (uptake and pulse-chase)

To assess the uptake amount and rate of transferrin (pulse assay) and recycling rate of transferrin (pulse-chase assay), 25.000 cells were seeded in a 96-well glass bottom imaging plate (PBK96G-1.5-F, MatTek USA) in BBB Identity Maintenance media on day 11. On day 14, cells were washed once with PBS and incubated with the assay medium (EGM, cAP-02, Angioproteomie) containing 1% Bovine serum albumin for at least 10 min. Labeled transferrin (T13342, ThermoFisher) at 25 µg/mL was then applied between 2 and 45 min to assess the time course of transferrin uptake. To assess the recycling rate, cells were treated with labeled transferrin (T13342, ThermoFisher) at 25 µg/mL for 20 min, followed by application of 10-fold higher concentration of unlabeled Holo-transferrin (T0665, 250 µg/mL) for different time points (0 min - 60 min). Cells were fixed in 4% PFA for 20 min and washed three times with PBS

before counterstaining with Phalloidin-Atto-647 (65906, Sigma) and DAPI (D9542, Sigma). Images were acquired with Opera Phenix High Content Imaging System (PerkinElmer) using the 40×/1.1 NA water long WD confocal objective, Binning 2x2, Camera ROI 2160x2160, 20 planes à 1 µm. Per time point, minimum 50 images were acquired.

Live imaging of endosomal pH

To estimate endosomal pH, 15.000 cells were seeded in each well of a 96-well glass bottom imaging plate (PBK96G-1.5-F, MatTek USA) in BBB Identity Maintenance media 72 h before the experiment. After washing with PBS, cells were incubated with 25 µg/ml of pHrodo™ Red Transferrin Conjugate (P35376, ThermoFisher) and 25 µg/ml of AF647-conjugated Transferrin (T23366, ThermoFisher) for 10 minutes at 37°C 5% CO_2. Cells were washed twice with BBB Identity Maintenance media, and were subsequently imaged with a DMI8 fluorescence microscope (Leica Microsystems) equipped with a stage incubator controlling at 37 ºC and 5% CO2. Full frame 1024 x 1024 images were acquired with HCX PL APO 100X/1.4 NA oil objectives with a resolution of 0.288 µm, three z-stacks with 0.75 µm step size. Using maximum projection images, vesicles containing both AlexaFluor647-conjugated transferrin and pHrodo™ Red Transferrin were detected (colocalization threshold 0.35). Per endosome, the ratio of integrated vesicular intensity of pHrodo™ Red Transferrin and AlexaFluor647- conjugated transferrin was calculated (MotionTracking 8.97, http://motiontracking.mpi-cbg.de/get/) as previously described [76].

Live imaging of sorting tubule biogenesis

To assess the formation of sorting tubules, 15.000 cells were seeded in a 96-well glass bottom imaging plate (PBK96G-1.5-F, MatTek USA) in BBB Identity Maintenance media 48 h before the experiment. After washing with PBS, cells were incubated with 25 µg/ml of fluorescently labeled transferrin (T13342, ThermoFisher) for at least three hours at 37°C 5% CO_2. After washing with BBB Identity maintenance media, cells were imaged with a DMI-8 TIRF microscope (Leica Microsystems) equipped with a stage incubator controlling at 37 ºC and 5% CO_2.

Full frame 1024 x 1024 images were acquired with HCX PL APO 100X/1.4 NA oil objectives with a resolution of 0.288 µm, two z-stacks with 1 µm step size. Single color images were acquired with a 488 nm TIRF laser for a final rate of 2 frames per second for 1 min each. The number of tubules per cell occurring within 1 min acquisition was quantified manually in the maximum intensity projection of the z-sections for each movie by two independent blinded raters.

Immunostainings

After fixation of the cells with 4% PFA (15710, Electron Microscopy Science), cells were quickly washed with PBS for three times, before permeabilized with 4% gelatin (G7041, Sigma) + 0.1% Saponin (84510, Sigma) in PBS for 10 min at RT. Primary antibodies were incubated overnight at 4 °C (see Table 1). After washing three times with PBS, fluorescently labeled secondary antibodies and Phalloidin-Atto-647 (65906, Sigma) were applied for one hour at room temperature (RT, see Table 2). Nuclei were counterstained with DAPI (Sigma, D9542) for 10 min at RT. For representative images of PECAM1, VE-Cadherin, Claudin-5, SLC2A1 (GLUT-1), ABCB1 (PgP), ABCC1 (MRP1), ABCA1 (CERP), confocal images at SP-8 using 63×/1.2 NA objective or a DMI-8 TIRF microscope at 20×/0.55 NA magnification (Leica Microsystems) were acquired. For quantification of iron-related proteins (FTH and DMT-1), Opera Phenix High Content Imaging System (PerkinElmer) was used at 40× magnification. Bright field images were acquired with an IX83 microscope (Olympus) with a 10×/0.3 NA objective.

Immunostainings (microphysiological system)

The OrganoPlate cultures were fixed with 3.7% formaldehyde (252,549, Sigma) or 100% methanol (494,437, Sigma). Immunostaining was performed as previously reported [5,10]. Briefly, cultures were permeabilized using Triton X-100 (T8787, Sigma) for 10 min followed by a blocking step with a buffer containing FBS (A5670801, Thermo Fisher), bovine serum albumin (BSA, 5217, R&D Systems) and Tween-20 (P9416, Sigma) for 45 min. Primary antibodies (see Table 1) were incubated in the blocking buffer overnight at 4°C after which

secondary antibodies (see Table 2) were incubated for 1 h at RT. Nuclei were stained with Hoechst (H3570, ThermoFisher). Representative images were acquired at 10×/0.45 magnification using ImageXpress Micro XLS and Micro XLS-C HCI Systems (Molecular Devices).

High content imaging and image analysis for transferrin kinetics and immunostainings

The Perkin Elmer's Harmony high-content analysis software 5.1 (HH17000001) was used to set up the plate dimensions enabling the fast and efficient imaging with the Opera Phenix High Content Imaging System (PerkinElmer). For transferrin kinetics and immunostainings for iron-related protein experiments, we used the 40× long WD confocal objective. Per well, 50 images were acquired, 20 planes per image with a section thickness of 1 µm per plane. Appropriate channels were selected (DAPI, Alexa-488, Alexa-647) and exposure time as well as focus height was set accordingly and kept the same between wells. Perkin Elmer's Harmony high-content analysis software 5.1 was used to analyze the images. In brief, basic flat field correction and maximum projection was applied. Cell area was determined by a binary threshold mask using the Phalloidin signal. Sum intensity of the respective markers (transferrin-488, FTH, FPN, DMT-1) was calculated within the cell area and normalized to its area. Linear background reduction was applied. For assessment of the transferrin uptake rate, intensity values were normalized to the 10 min time point, while for transferrin recycling rate, intensity values were normalized to the time point of 20 min pulse, no chase.

Confocal microscopy and image analysis of TfR1 and EEA1

For quantification of TfR1 or EEA1 in iCE-BECs, ten images per condition were acquired using the confocal microscope with a 63×/1.2 NA objective. Pixel size was adjusted to 100 nm at 1024x1024 frame size. Per image, five z-stacks with a step size of 0.5 µm were used. Pinhole was adjusted to an optical thickness of 1 um and sequential acquisition between stacks was selected. Laser power was adjusted according to the marker expression and not changed between experimental groups. Vesicles containing EEA1 or TfR1 were detected

and mean integrated vesicular intensity within cells (absolute threshold for Phalloidin) was calculated, normalized to the cell area (MotionTracking 8.97, http://motiontracking.mpi-cbg.de/get/) as previously described[11].

Supplementary Video S1 - related to Figure 5.

Representative video of transferrin (green) intracellular transport in live iCE-BECs (isogenic pair 1) with ApoE3 gene variant. Cells were incubated with fluorescently labeled transferrin for three hours and then videos of one minute were acquired at 100× using a Widefield microscope. Representative image frames of those videos are shown in Figure 5G.

Supplementary Video S2 - related to Figure 5.

Representative video of transferrin (green) intracellular transport in live iCE-BECs (isogenic pair 1) with ApoE4 gene variant. Cells were incubated with fluorescently labeled transferrin for three hours and then videos of one minute were acquired at 100× using a Widefield microscope. Representative image frames of those videos are shown in Figure 5G.

1. Lu TM, Houghton S, Magdeldin T, et al. Pluripotent stem cell-derived epithelium misidentified as brain microvascular endothelium requires ETS factors to acquire vascular fate. *Proceedings of the National Academy of Sciences.* 2021;118(8):e2016950118.
2. Ewels P, Magnusson M, Lundin S, Käller M. MultiQC: summarize analysis results for multiple tools and samples in a single report. *Bioinformatics.* 2016;32(19):3047-3048.
3. Liao Y, Smyth GK, Shi W. featureCounts: an efficient general purpose program for assigning sequence reads to genomic features. *Bioinformatics.* 2013;30(7):923-930.
4. Robinson MD, McCarthy DJ, Smyth GK. edgeR: a Bioconductor package for differential expression analysis of digital gene expression data. *Bioinformatics.* 2010;26(1):139-140.
5. Wevers NR, Kasi DG, Gray T, et al. A perfused human blood-brain barrier on-a-chip for high-throughput assessment of barrier function and antibody transport. *Fluids Barriers CNS.* 2018;15(1):23.
6. Ragelle H, Dernick K, Khemais S, et al. Human Retinal Microvasculature-on-a-Chip for Drug Discovery. *Advanced Healthcare Materials.* 2020;9(21):2001531.
7. Breuer W, Epsztejn S, Millgram P, Cabantchik IZ. Transport of iron and other transition metals into cells as revealed by a fluorescent probe. *Am J Physiol.* 1995;268(6 Pt 1):C1354-1361.
8. Tenopoulou M, Kurz T, Doulias P-T, Galaris D, Brunk UT. Does the calcein-AM method assay the total cellular 'labile iron pool' or only a fraction of it? *The Biochemical journal.* 2007;403(2):261-266.
9. Thomas F, Serratrice G, Béguin C, et al. Calcein as a Fluorescent Probe for Ferric Iron: APPLICATION TO IRON NUTRITION IN PLANT CELLS *. *J Biol Chem.* 1999;274(19):13375-13383.
10. Nair AL, Groenendijk L, Overdevest R, et al. Human BBB-on-a-chip reveals barrier disruption, endothelial inflammation, and T cell migration under neuroinflammatory conditions. *Front Mol Neurosci.* 2023;16:1250123.
11. Rink J, Ghigo E, Kalaidzidis Y, Zerial M. Rab conversion as a mechanism of progression from early to late endosomes. *Cell.* 2005;122(5):735-749.

Supplementary Figures

Supplementary Figure 1 - related to Figure 1

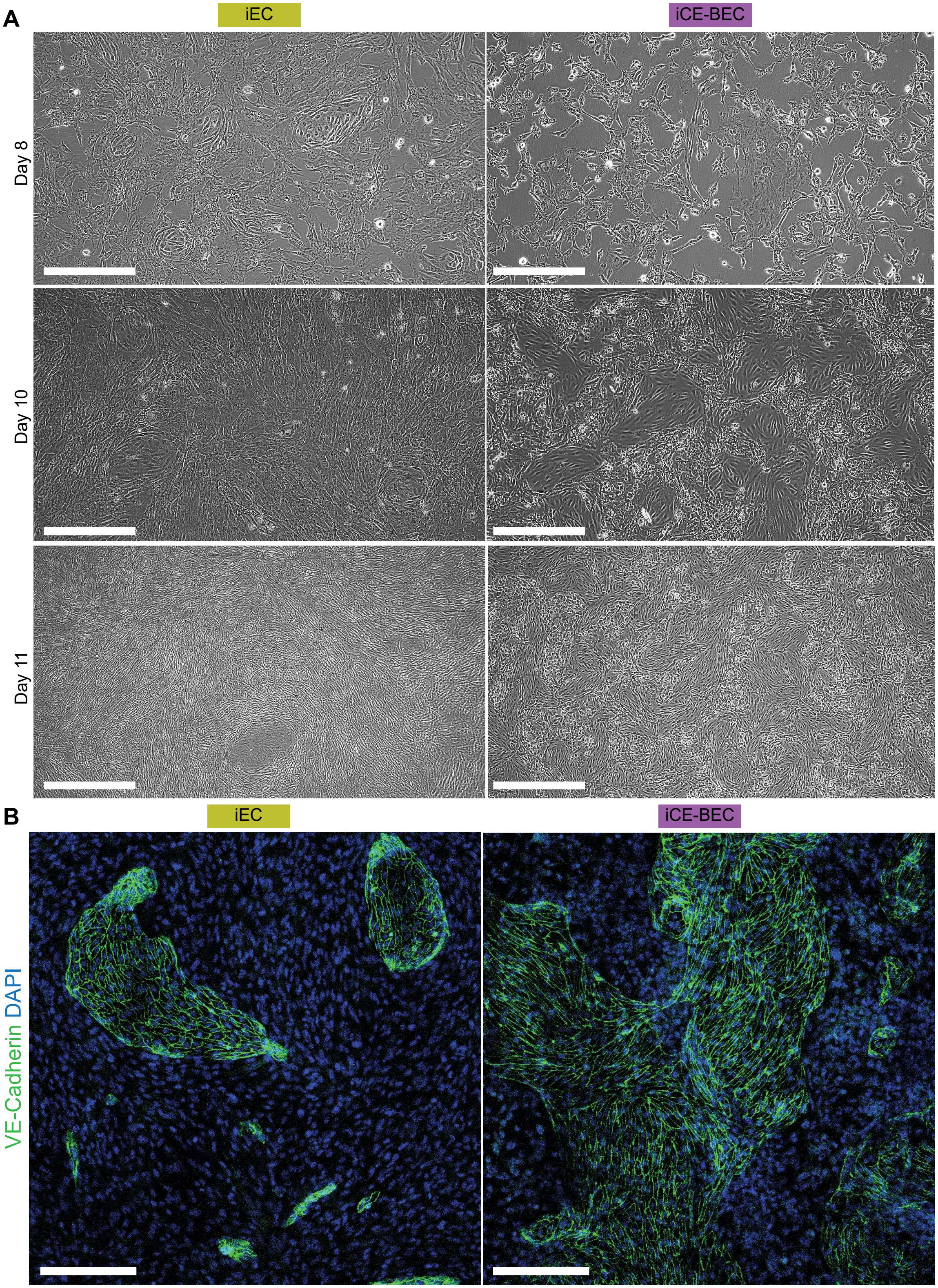

Supplementary Figure 1 - related to Figure 1. A, Representative brightfield images of iEC and iCE-BECs during the differentiation at day 8, day 10, and day 11. Scale bar, 500 µm. **B,** Representative fluorescence images of iEC and iCE-BECs after immunostaining for VE-Cadherin (green) at day 11 before MACS sorting. DAPI-stained nuclei are shown in blue. Scale bar, 250 µm.

Supplementary Figure 2 - related to Figure 2 and 3

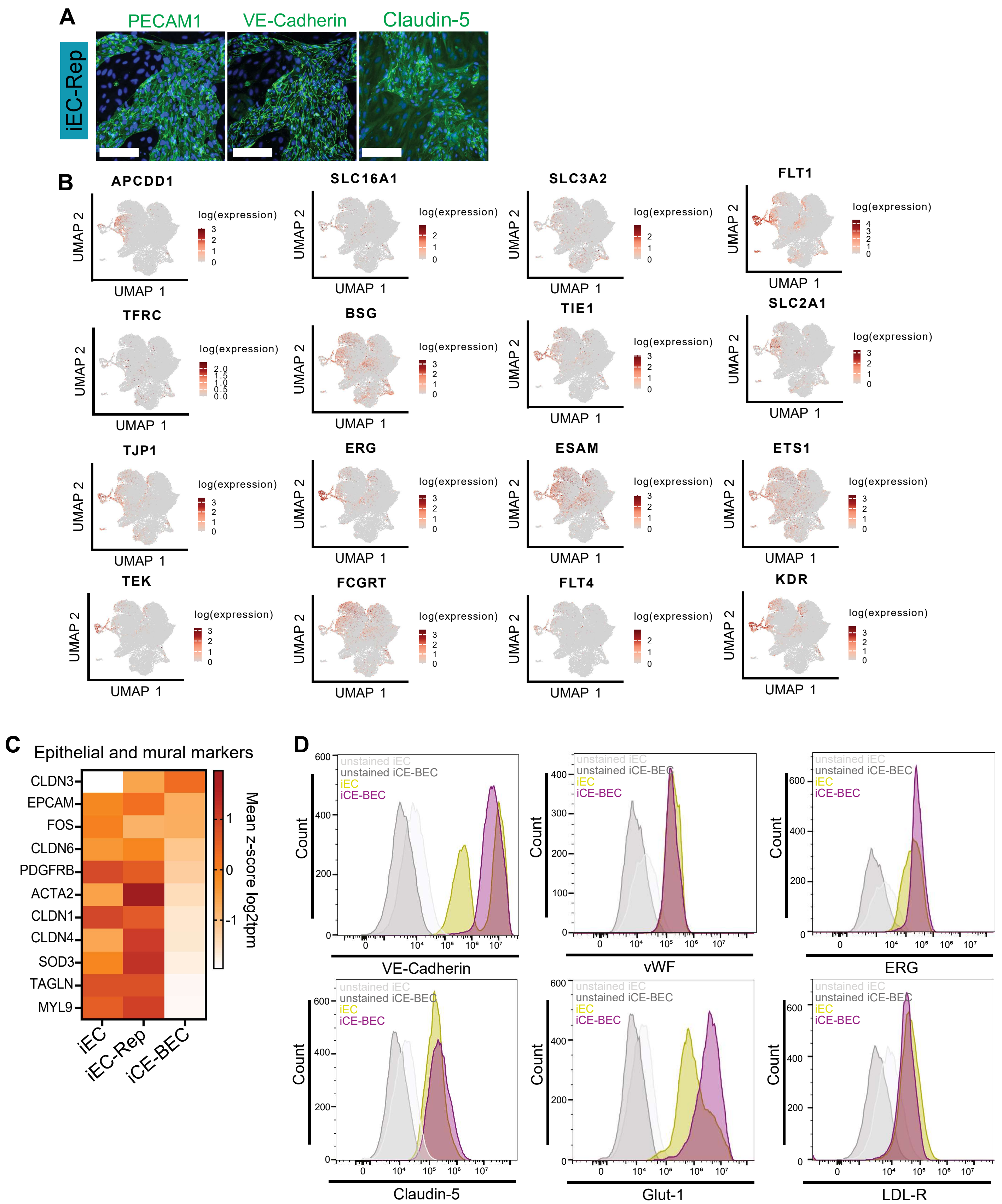

Supplementary Figure 2 - related to Figures 2 and 3.

A, Representative fluorescence images after immunostaining with endothelial-specific markers of iEC-rep at day 14. Cells are pseudo-colored showing PECAM1, VE-Cadherin or Claudin-5 in green, and DAPI-stained nuclei in blue. Scale bar, 200 µm. **B,** Feature plots showing normalized log expression of marker genes of endothelial and mural markers, plotted on the UMAP from Figure 2A. **C,** Bulk RNA-Seq heatmap showing expression of epithelial and mural markers across the three differentiation protocols, iEC, iEC-rep, and iCE-BECs. Values are expressed as mean z-score log_2tpm, with three independent differentiations per condition. **D,** Representative flow cytometry histograms showing fluorescence intensities of endothelial cell marker (ERG, vWF), tight junction proteins (VE-Cadherin, Claudin-5), transporters (GLUT1) and receptors (LDL-R) in PECAM1-positive single (live), iEC (yellow), iCE-BEC (purple) and their respective unstained control (light gray for iEC, dark grey for iCE-BEC). Intensity values are shown on the x-axis, while the y-axis displays the number of cells.

Supplementary Figure 3 - related to Figure 4

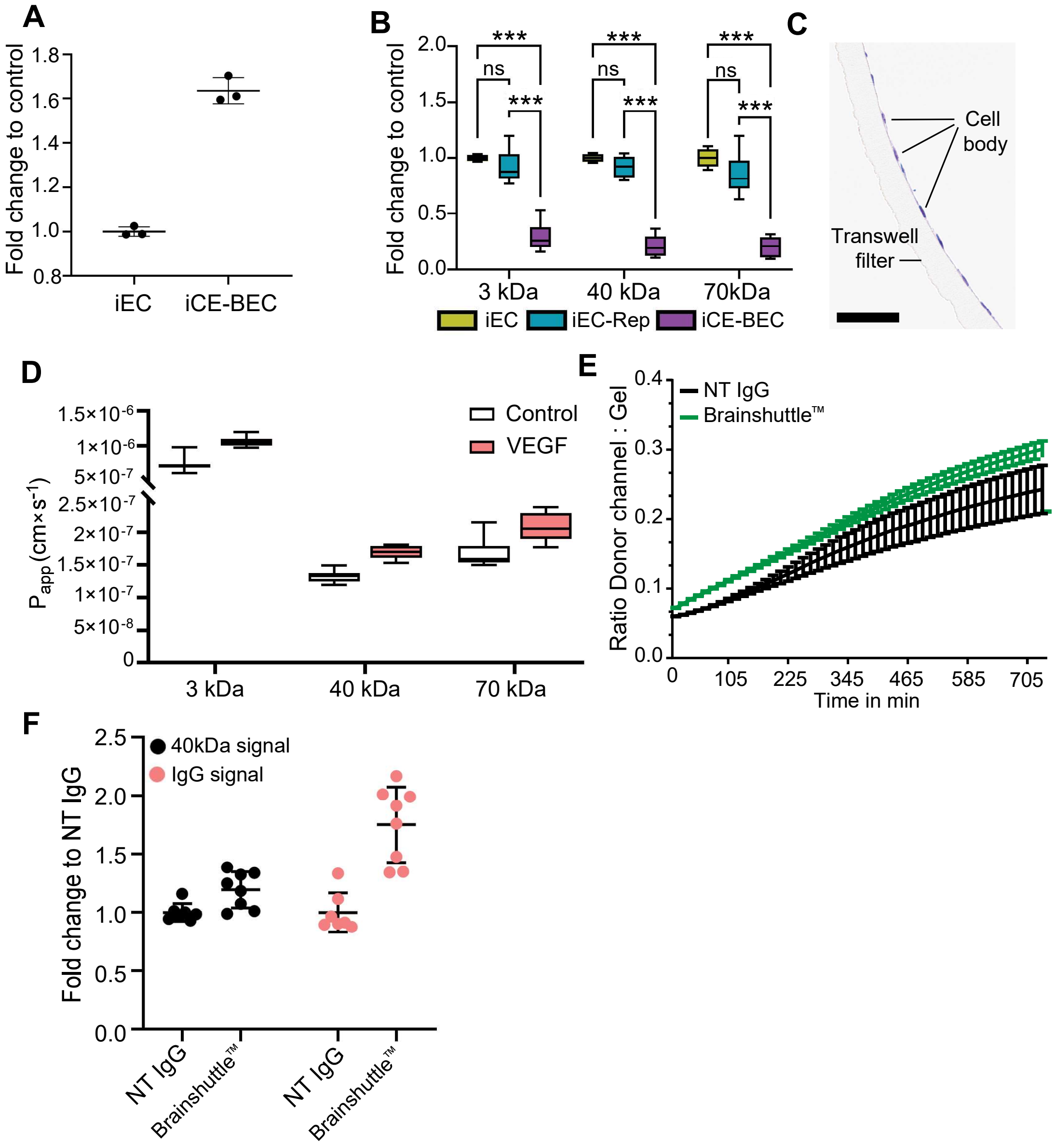

Supplementary Figure 3 - related to figure 4.
A, Normalized TEER values for iCE-BEC and iECs at 18 hours.

Data from 3 technical replicates per condition. TEER values were normalized to iECs for fold change comparison. **B,** Quantification of relative apparent permeability (P_{app}) for dextrans of different molecular weights of cells generated with the protocols described in Fig. 1A using a transwell system. Values were normalized to the apparent permeability of iECs. Graph shows boxplots with interquartile ranges and median. Lines show the 5th and 95th percentiles, data from n = 4 independent differentiations with 8 technical replicates per condition. Differences in apparent permeability are statistically significant as evaluated by Two-way ANOVA with Sidak multiple comparisons. ns, not statistically significant; ***, p <0.001. **C,** Haematoxylin and Eosin staining of iCE-BECs grown on a transwell filter showing a cell monolayer. Scale bar, 50 µm. **D,** Quantification of apparent permeability (Papp) of iCE-BECs to 3, 40 or 70 kDa dextran in basal conditions or after stimulation with 200 ng/mL VEGF-A for 24 hours. Graph shows boxplots with interquartile ranges and median. Lines show the 5th and 95th percentiles, data from one differentiation with at least 7 technical replicates per condition. **E,** Representative antibody transcytosis curves across iCE-BECs after incubation with 200 nM non-targeting IgG (NT IgG) or Brainshuttle™ antibody. Images were acquired immediately after incubation for 12 hours and ratio between donor channel and gel channel signals were plotted against time (see methods for details). Each curve shows mean ± SEM data from 8 chambers. **F,** Quantification of relative IgG transcytosis (IgG signal) and apparent permeability to 40 kDa dextran (40 kDa signal) across iCE-BECs after incubation with 200 nM non-targeting IgG (NT IgG) or a Brainshuttle™ antibody. Antibody and dextran values are measured in the same channels and are normalized relative to the NT IgG condition. Graph shows mean ± SD of one differentiation with 8 technical replicates per condition.

Supplementary Figure 4 - related to Figure 5

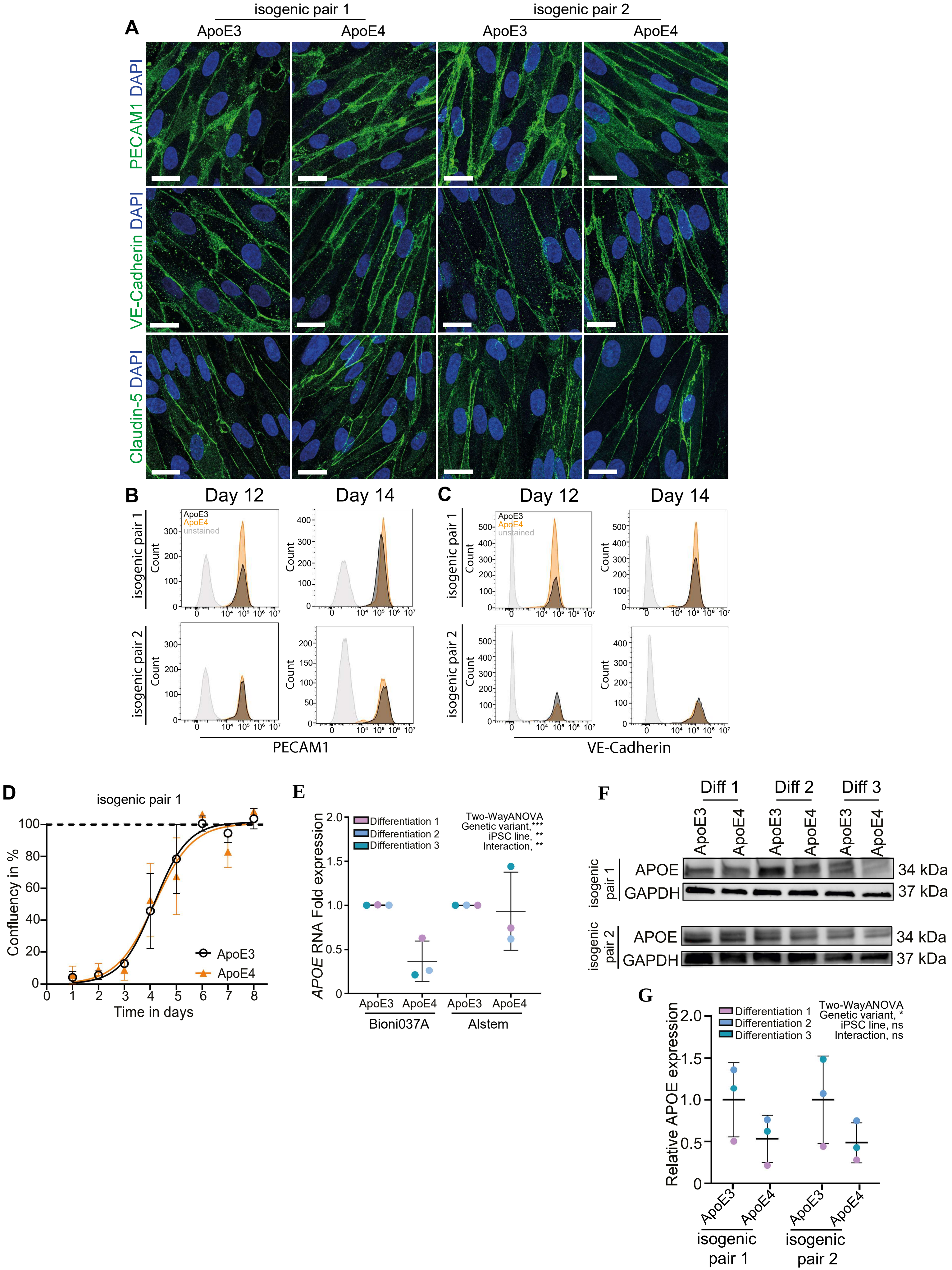

Supplementary Figure 4 - related to Figure 5.

A, Representative fluorescence images after immunostaining with endothelial-specific markers in iCE-BECs of two isogenic pairs (Bion037A, isogenic pair 1; Alstem, isogenic pair 2) with ApoE3 or ApoE4 genetic variant. Cells are pseudo-colored showing PECAM1, VE-Cadherin, ZO-1 or Claudin-5 in green, and DAPI-stained nuclei in blue. Scale bar, 20 µm. **B-C,** Representative flow cytometry histograms showing fluorescence intensities of PECAM1 (B), VE-Cadherin (C) in iCE-BECs with ApoE3 (black) or ApoE4 (orange) genetic variants or unstained control (grey). Intensity values are shown on the x-axis, while the y-axis displays the number of cells at day 12 and day 14.

D, Assessment of proliferation rate of iPSC line (isogenic pair 1) with ApoE3 and ApoE4 genetic variants over eight consecutive days. Live/dead staining and live imaging of whole wells was performed with Opera Phenix High Content Imaging System (PerkinElmer) at 20× magnification with three wells per condition and time point. Live cell area was measured by absolute threshold and expressed as percentage of total well area (confluency) ± SD. Non-linear regression (logistic growth) was performed. **E,** Quantification of relative *ApoE* mRNA expression by quantitative PCR. Graph shows mean ± SD of both isogenic pairs with each $n = 3$ independent differentiations with 3 technical replicates per experiment. Points represent independent differentiations of both isogenic pairs. Differences in *ApoE* mRNA expression were evaluated by a Two-Way ANOVA: significant main effect of genetic variant on *ApoE* expression, $p < 0.001$, significant main effect of iPS line on *ApoE* expression, $p < 0.01$, and significant interaction between iPS line and genetic variant, $p < 0.01$. **F,** Representative Western Blot image showing ApoE protein expression in iCE-BECs with ApoE3 or ApoE4 genetic variants. Data come both isogenic pairs from three independent differentiations (Diff). **G,** Quantification of relative APOE protein expression from Western Blot in (**F**). Graphs show mean ± SD. Points represent independent differentiations. *, $p < 0.05$, significant main effect of genetic variant on APOE expression, no significant effect of iPSC line on APOE expression and no significant interaction between iPSC line and genetic variant by Two-Way ANOVA with $n = 3$ independent differentiations.

Supplementary Figure 5 - related to Figure 6

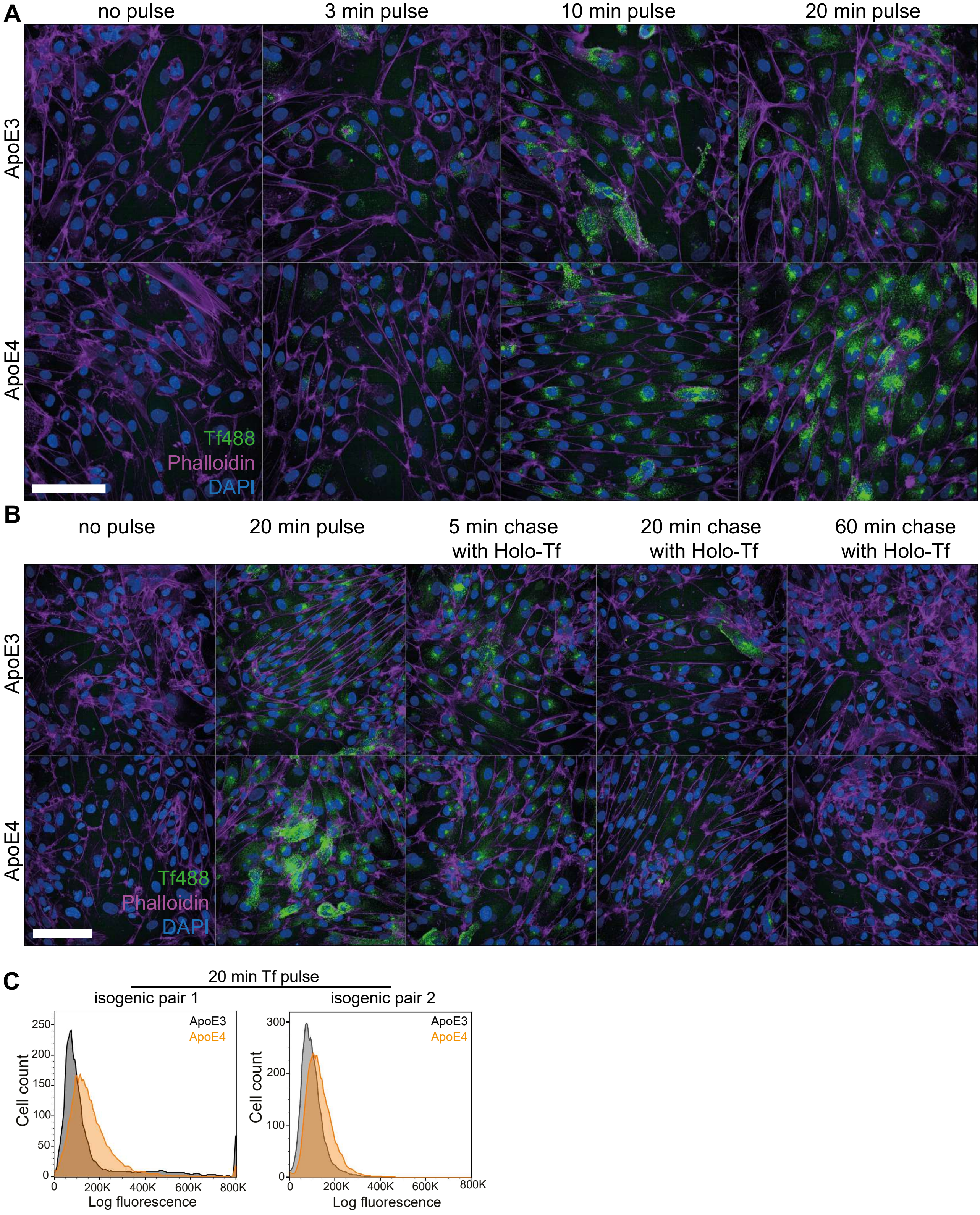

Supplementary Figure 5 - related to Figure 6.

A, Representative maximum projection confocal images of iCE-BECs with ApoE gene variants from pulse assay and **B,** pulse-chase assay assessing transferrin trafficking kinetics. Briefly, cells were incubated with fluorescently labeled transferrin and fixed after different time points for continuous uptake (pulse assay) while for recycling assessment (pulse-chase assays), cells were incubated for 20 min with fluorescently labeled transferrin followed by incubation of 10-fold higher concentration of unlabeled holo-Transferrin for different time points. Cells are pseudo-colored showing Transferrin in green, Phalloidin in magenta, and DAPI-stained nuclei in blue. Scale bar, 100 µm. For each time point, 50 images were acquired at 40× using a high content screening system, maximal projections were used to quantify sum intensity of transferrin in Phalloidin-positive area. **C,** Flow cytometry assessment of transferrin uptake after 20 min incubation in live iCE-BECs of both isogenic pairs with ApoE3 and ApoE4 genetic variants. Histograms show log fluorescence intensities of fluorescently labeled transferrin on the x-axis and the number of cells on the y-axis. ApoE3 iCE-BECs are shown in black, ApoE4 iCE-BECs in orange.

Supplementary Figure 6 - related to Figure 7

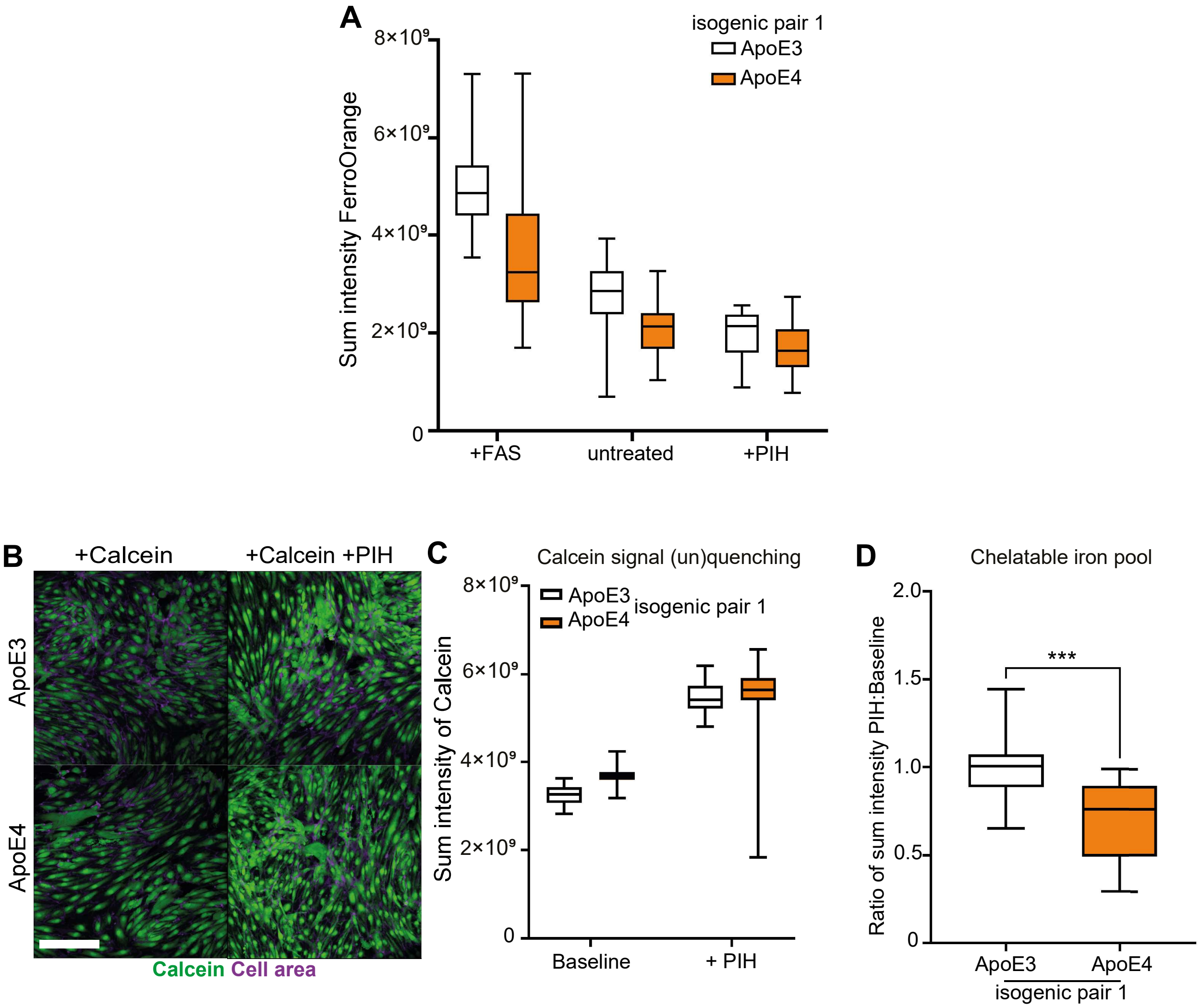

Supplementary Figure 6 - related to Figure 7.

A, Mean intensity of FerroOrange in iCE-BECs with ApoE3 or

ApoE4 gene variant (isogenic pair 1). As controls, cells have been treated with an iron donor ferrous ammonium sulfate (FAS) or iron chelator pyridoxal isonicotinoyl hydrazone (PIH) for 30 min before incubating with FerroOrange, a fluorescent probe that specifically detects labile iron (II) ions (Fe^{2+}) in live cells. Graph shows boxplots with interquartile ranges and median. Lines show the 5th and 95th percentiles, data from one differentiation, 40 images per condition have been acquired at 63× using a high content screening system, maximal projections were used to quantify sum intensity of FerroOrange within cells. **B,** Representative images of iCE-BECs with ApoE gene variants (isogenic pair 1) incubated with the metal-sensitive probe calcein acetoxymethyl ester (calcein-AM), which quenches its green fluorescence when binding to iron and unquenches upon iron chelator treatment with iron chelator pyridoxal isonicotinoyl hydrazone (PIH). Cells were treated with an iron chelator PIH or left untreated. Cellular calcein fluorescence was measured in live cells using high content screening system at 20×. Cells are pseudo-colored showing Calcein in green, plasma membrane in magenta. Scale bar, 200 µm.**C,** Sum intensity of calcein was normalized per cell area shown in a representative experiment illustrating the calcein signal (un)quenching upon iron chelator (+PIH) treatment. **D,** The ratio between the mean intensity of Calcein within the cell area in untreated cells (baseline) and iron chelator-treated cells (+PIH) was calculated, reflecting the amount of the labile iron pool. Graph shows boxplots with interquartile ranges and median. Lines show the 5th and 95th percentiles, data from isogenic pair 1 with n = 3 independent differentiations with 120 images per experiment. Differences in the FerroOrange intensity between ApoE genetic variants are statistically significant as evaluated by the Mann-Whitney-U test ($p < 0.001$).

Supplementary Figure 7 - related to Discussion

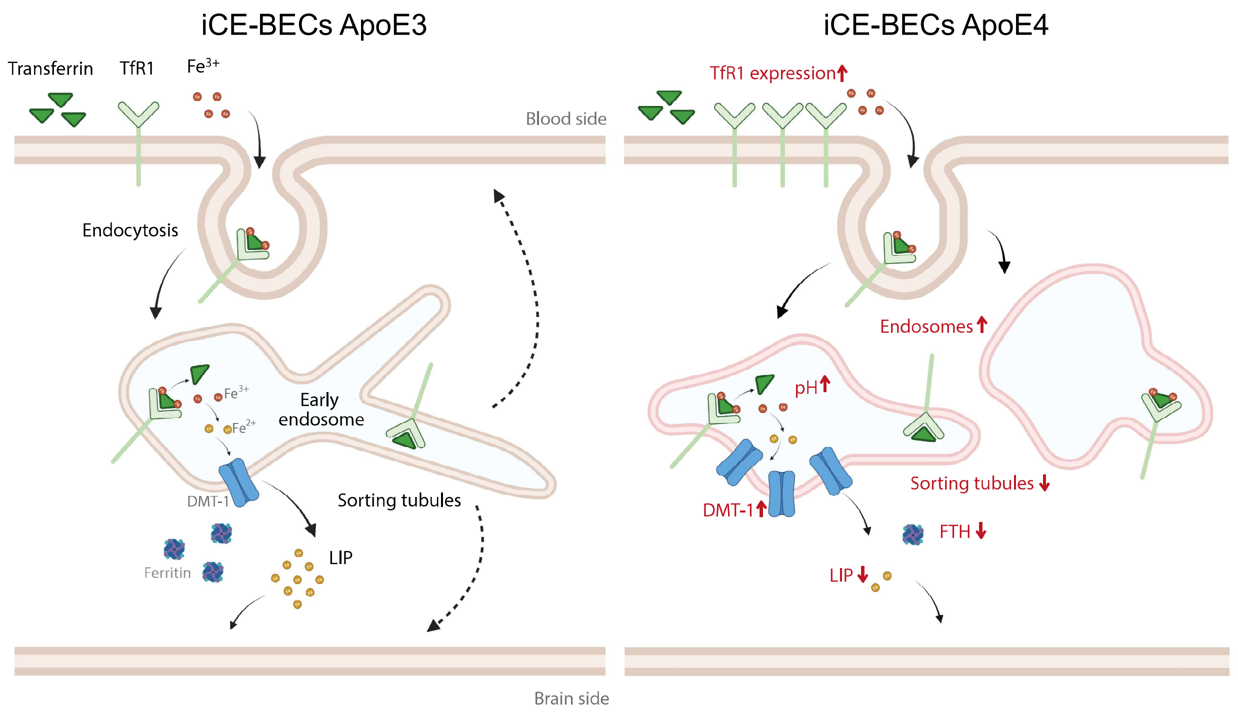

Supplementary Figure 7 - related to discussion.
Schematic summarizing intracellular transport of transferrin and

iron in BECs with ApoE3 genetic variant and the key changes occurring in ApoE4 iCE-BECs: a) early endosome enlargement, increased pH and reduced sorting tubule biogenesis. b) Reduced intracellular labile iron pool (LIP), potentially caused by defects in endosomal maturation. c) Changes in expression of proteins regulated by iron-responsive elements, including Divalent metal transporter 1 (DMT-1), transferrin receptor TfR1 (both increased) and Ferritin (FTH, also reduced). d) Increased transferrin uptake driven by higher TfR1 expression. Figure created in biorender.com.
